# SCL15 Regulates the Release of Seed Dormancy in *Arabidopsis thaliana* by Integrating the Circadian Clock, Hormonal Signals and Cell Wall Remodelling

**DOI:** 10.1111/ppl.70467

**Published:** 2025-09-08

**Authors:** Ming‐Jun Gao, Qi Chen, Cathy Coutu, Fuyou Fu, Bianyun Yu, Xiang Li, Z. Jeffrey Chen, Dwayne Hegedus

**Affiliations:** ^1^ Agriculture and Agri‐Food Canada, Saskatoon Research and Development Centre Saskatoon Saskatchewan Canada; ^2^ State Key Laboratory of Tea Plant Biology and Utilization Anhui Agricultural University Hefei Anhui China; ^3^ Department of Molecular Biosciences The University of Texas at Austin Austin Texas USA

**Keywords:** abscisic acid, auxin, cell wall, circadian clock, endosperm, seed dormancy

## Abstract

Dormancy release and germination of the seed are two separate, but continuous phases controlled by both external (e.g., light and temperature) and internal (e.g., circadian clock and hormones) cues. In eudicot seeds, the endosperm tissues play a key role in dormancy release and germination through dynamic modulation of wall components and biomechanics. However, the mode of action by which the circadian oscillator influences dormancy release by modulation of endosperm wall biomechanics remains elusive. SCARECROW‐LIKE15 (SCL15) represses embryonic gene expression in seedlings through interaction with HISTONE DEACETYLASE19 (HDA19) in *Arabidopsis thaliana* . Here, we report that SCL15 plays a positive role in primary dormancy release, which is associated with gene expression changes in circadian, abscisic acid, auxin and cell wall (CW) remodelling pathways, based on studies using *SCL15* mutant and *Napin* promoter‐driven *SCL15* expression lines. SCL15 was found to affect the expression of genes whose products modify endosperm wall biomechanical features, possibly through regulation of local auxin accumulation and evening‐phased clock components. RNA‐seq analysis supported the notion that dormancy release is associated with changes in the expression of genes associated with circadian and hormone‐mediated pathways, which in turn affect CW structure.

## 1 Introduction

Seed development is a sophisticated process that begins with double fertilization, followed by embryogenesis and seed maturation. The initiation of the seed maturation program depends on the precise spatio‐temporal regulation of gene expression and is largely controlled by the LAFL family transcription factors (TFs) named after three representative B3 domain TFs: LEAFY COTYLEDON 2 (LEC2), ABSCISIC ACID INSENSITIVE 3 (ABI3) and FUSCA3 (FUS3), as well as the HEME ACTIVATOR PROTEIN 3 (HAP3) Family CCAAT‐box binding factor, LEC1 (Gazzarrini and Song 2024). These TFs work in concert with phytohormones, such as abscisic acid (ABA), gibberellin (GA) and auxin, along with other factors. The seed maturation process consists of two developmental phases: reserve accumulation and desiccation tolerance. Molecular determinants for desiccation tolerance, dormancy competence and germination potential are crucial for seed‐to‐seedling phase transition, a process that initiates at the later maturation stage and develops throughout the desiccation stage. This developmental progression enables orthodox seeds to survive dehydration and after‐ripening in a state of metabolic quiescence, and to reactivate cellular functions upon water imbibition (Shu et al. 2016; Matilla 2022).

The phase transition from seed dormancy to germination depends on interactions between seed tissues, hormonal signals and cell wall (CW) remodelling mechanisms that either weaken or stiffen mechanical tissues (Nonogaki 2019; Cosgrove 2022). The coordinated development and differentiation of the three seed compartments (the testa, endosperm and embryo) underline the seed maturation program. Endosperm tissues play a key role in dormancy release and germination in many eudicot seeds (Sato and Kohler 2022). Micropylar endosperm (ME) weakening is a prerequisite for radicle emergence and the completion of seed germination (Bewley 1997; Müller et al. 2006; Nonogaki 2019; Chandrasekaran et al. 2022). ME weakening is directed by environmentally‐mediated, hormonal mechanisms involving auxin, ABA, GAs and ethylene through the activity of CW remodelling proteins (CWRPs), such as expansins (EXPs) and mannanases (MANs), that disrupt CW polysaccharide bonds (Lee et al. 2012; Graeber et al. 2014; Cosgrove 2022). ABA inhibits seed germination by suppressing ME weakening and embryo growth (Müller et al. 2006; Graeber et al. 2014). In dormant brassicaceae seeds, ABA is primarily produced in the endosperm and then exported into the embryo to inhibit germination (Kang et al. 2015; Sano and Marion‐Poll 2021; Sajeev et al. 2024), indicating the essential role of the endosperm for dormancy at both physical and biochemical levels. Auxin levels and gradients modulate the local apoplastic pH environment by regulating plasma membrane (PM)‐localized H^+^‐ATPases; this alters the activities of CW‐bound remodelling enzymes and proteins, such as MANs (Hocq et al. 2017; Iglesias‐Fernández et al. 2020; Lin et al. 2021). In addition, the ME has been proposed to mediate communication in environmental sensing and responses (Yan et al. 2014). These phase transition processes are complex and our understanding of how these cues are coordinated and integrated to result in endosperm rupture and dormancy release remains limited (Shu et al. 2016; Penfield 2017; Klupczyńska and Pawłowski 2021; Sajeev et al. 2024; Xu et al. 2024).

Plant CWs are formed from different polysaccharides, including cellulose, hemicellulose and pectins. In different tissues and cell types, CWs may have different types and proportions of these polysaccharides and establish different physical and chemical interactions with each other; thereby conferring different properties to the walls. In eudicot seeds, endosperm CWs have a composition and architecture distinct from those of embryo CWs, of which the former contain a small amount of cellulose, large amounts of hemicelluloses (e.g., xyloglucans [XyG] and mannan) and unmethylesterified pectin homogalacturonans (HGs), along with abundant pectic arabinans and arabinan‐rich rhamnogalacturonan‐I (RG‐I) (Lee et al. 2012). The fine‐tuning of XyG hydrolysis in CWs is essential for the control of ME rupture in response to exogenous cues (e.g., temperature) and endogenous signals (e.g., hormones) (Sechet et al. 2016; Shigeyama et al. 2016). The degree of methylesterification of HGs constitutes a key element in the control of wall stiffness (Hocq et al. 2017). In *Arabidopsis thaliana* (Arabidopsis), endosperm walls often contain mannan‐rich hemicellulose as a major wall component (Iglesias‐Fernández et al. 2011). Many CW metabolism proteins and enzymes have been identified in the endosperm (Sechet et al. 2016), several of which are involved in endosperm wall modification during germination, such as the positive dormancy regulator alpha‐xylosidase 1 (XYL1) (Shigeyama et al. 2016; Sechet et al. 2016; Nonogaki 2019).

External light and the internal circadian clock are essential for the control of dormancy release and germination processes. ABA, auxin and the biosynthesis and remodelling of the CW are regulated by the circadian clock (Penfield and Hall 2009; Rawat et al. 2009; Lee et al. 2016; Adams et al. 2018). The core of the circadian oscillator is composed of a set of transcription/translation feedback loops connected by repressive feedback (Hsu and Harmer 2014). Over 20 circadian clock‐regulated genes have been identified in Arabidopsis, including the morning‐phased core *MYB‐like TF* *CIRCADIAN* *CLOCK‐ASSOCIATED1* ( *CCA1* ) and *LATE ELONGATED HYPOCOTYL* ( *LHY* ). A subset of evening‐phased genes, such as *TIMING OF CAB EXPRESSION1* ( *TOC1* ), are repressed by CCA1 and LHY (Hsu and Harmer 2014). As major components of the circadian clock network, the morning‐phased CCA1 (Penfield and Hall 2009; Adams et al. 2018) and REVEILLE1 (RVE1) (Rawat et al. 2009; Jiang et al. 2016) positively regulate seed dormancy through modulation of ABA and auxin signalling, respectively. CW synthesis and remodelling are under hormonal and circadian clock control, of which CW synthesis occurs during the day and wall loosening and elongation take place at night (Verbančič et al. 2018). Accordingly, many morning‐phased clock regulators promote seed dormancy, while some evening‐phased circadian regulators promote dormancy release (Zha et al. 2020). However, the molecular and physiological mechanisms by which the circadian oscillator drives rhythmic changes in hormone (e.g., auxin and ABA) levels and influences CW biomechanics and subsequent dormancy release remain elusive. SCARECROW‐LIKE15 (SCL15) represses embryonic gene expression in seedlings through physical interaction with the chromatin remodelling factor histone deacetylase 19 (HDA19) (Gao et al. 2015). SCL15 is also involved in regulating the transition from embryo to seedling (Gao et al. 2015). Here, we report germination studies and genome‐wide analysis of transcription in maturing seeds of the *SCL15* mutant (*scl15‐1*) and *Napin* promoter‐driven *SCL15* expression lines, demonstrating that SCL15 acts as a negative regulator of primary dormancy in Arabidopsis. *SCL15* regulates gene expression changes in circadian‐ and hormone‐mediated (ABA and auxin) pathways, which may in turn affect endosperm CW structural changes leading to dormancy release. A deeper understanding of seed dormancy and germination will help to improve crop yield and resilience to climate change.

## 2 Methods

### 2.1 Plant Materials and Growth Conditions

Arabidopsis T‐DNA insertion lines SALK_110871 (*scl15‐1*) and GK292A11 (*scl15‐2*) (NASC ID: N427947) were obtained from the Arabidopsis Biological Resource Centre (ABRC, Ohio State University) and the Eurasian Arabidopsis Stock Centre (uNASC, University of Nottingham) collections, respectively. These two mutants showed similar plant phenotypes in terms of seedling growth, flowering and silique development (Gao et al. 2015); therefore, only *scl15‐1* was used for further studies. Plant growth conditions are as previously described (Gao et al. 2009, 2015).

### 2.2 Plasmid Construction and Plant Transformation

To generate the *SCL15* over‐expression construct, the full‐length *SCL15* protein coding region was amplified by PCR using primers SCL15ox‐F1 and SCL15ox‐R2 (Table S1). The gene was subcloned into the *Not*I/*Sac*I sites downstream of the *CaMV 35S* promoter in pBI121 (Clontech) and the resultant construct was designated as 35S:SCL15. The *CaMV 35S* promoter was then replaced with the *Napin* promoter (Rask et al. 1998) and the resultant construct was designated as Napin:SCL15. These plasmids were introduced into *Agrobacterium tumefaciens* strain GV3101 followed by transformation into *A. thaliana* cv. Columbia‐0 (Col‐0) (Clough and Bent 1998). Transgenic lines that displayed a 3:1 segregation ratio in T2 populations were selected for further analysis. Homozygous T5 35S:SCL15 and Napin:SCL15 lines were used.

### 2.3 Germination Assays

Surface‐sterilized seeds were plated on 1/2 Murashige and Skoog (MS) medium containing 0.55% agar and 1% sucrose. After imbibition for 3 d in the dark at 4°C, the seeds were grown at 22°C under continuous light. Between 100 and 110 seeds from a single individual plant were placed on each Petri dish, and each germination experiment was conducted with at least three replicates per genotype. Protrusion of the radicle was regarded as the completion of seed germination. Germination rate was determined at different time points for 1 to 7 days of imbibition, and the average germination ratio (%) was determined. For ABA and ABA biosynthesis inhibitor treatments, sterilized and stratified seeds were plated onto 1/2 MS medium supplemented with various concentrations of ABA (A1049; Sigma‐Aldrich) or nordihydroguaiaretic acid (NDGA) (74,540; Sigma‐Aldrich). For auxin and auxin transport inhibitor treatments, seeds were germinated on medium supplemented with various concentrations of 2,4‐dichlorophenoxyacetic acid (2,4‐D) (76,514; Sigma‐Aldrich) or 2,3,5‐triiodobenzoid acid (TIBA) (T5910; Sigma‐Aldrich).

### 2.4 RNA Extraction and Quantitative Reverse Transcription (qRT)‐PCR Analysis

Total RNA was isolated from seedlings using the RNeasy Plant Mini Kit (Qiagen) and from siliques using the method described by Suzuki et al. (2004). qRT–PCR was performed as previously described (Gao et al. 2015). *ACT2* and *Ef‐1α* were used as the reference genes (Gao et al. 2009).

The gene‐specific primers for qRT‐PCR have been described previously (Gao et al. 2009, 2015) and those for other genes are listed in Table S1. All samples were evaluated in triplicate; the results presented are the mean (±SD) of three biological replicates.

### 2.5 RNA Sequencing and Data Analysis

Total RNA was isolated from siliques with three biological replicates of wild type Col‐0, the *scl15‐1* mutant and Napin:SCL15 lines. Silique samples were harvested from Col‐0 and Napin:SCL15 lines at 16–18 days post‐anthesis (DPA), whereas samples from *scl15‐1* were collected at 18–20 DPA due to *scl15‐1* silique development being delayed by 2 days. cDNA libraries were prepared using the TruSeq Stranded mRNA and Total RNA Library Prep kits with TruSeq LT adaptors (Illumina Inc.). The tagged libraries were sequenced using a HiSeq 2500 (Illumina Inc.). The short‐read sequence data from the nine libraries were deposited in the NCBI GEO database (accession GSE160707). The CLC tool ‘Differential Expression for Two Groups’ was used to perform three pairwise statistical comparisons: *scl15‐1* versus Col‐0, Napin:SCL15 versus Col‐0, and *scl15‐1* versus Napin:SCL15. Genes were considered differentially expressed if the log of the fold change was < −1 or > 1 and the false discovery rate (FDR) was < 0.05. CLC also automatically performs optimized normalization for comparison between libraries when statistical comparisons are performed. Expression changes were said to be significant if the FDR was < 0.05 and the absolute value of the relative fold change was > 1.5. Genes expressed in Arabidopsis valves rather than in maturing seeds were filtered out using previously published RNA‐seq datasets with genes expressed at 16 days after flowering (DAF) (Aulakh and Durrett 2019) and validated using Arabidopsis eFP Browser (https://bar.utoronto.ca/efp/) and mRNA localization datasets in mature seeds (Belmonte et al. 2013). The results of RNA‐Seq were validated by qRT‐PCR for several groups of candidate genes.

Gene Ontology (GO) categories that were significantly over‐represented among differentially expressed genes in the *scl15‐1* maturing seeds were determined using both the Biological Network Gene Ontology tool (BiNGO) plugin for Cytoscape v3.10.2 (Smoot et al. 2011) with settings of hypergeometric test and a Benjamini & Hochberg FDR correction with a significance level of 0.05, and agriGO v2.0 analysis (Tian et al. 2017). Cis‐element motif prediction was conducted according to MEME (Bailey et al. 2009). Venn diagrams were generated using the R package Venn Diagram (version 1.6.20) (Chen and Boutros 2011).

## 3 Results and Discussion

### 3.1 *SCL15* Mutation Confers Deeper Dormancy

**FIGURE 1:**
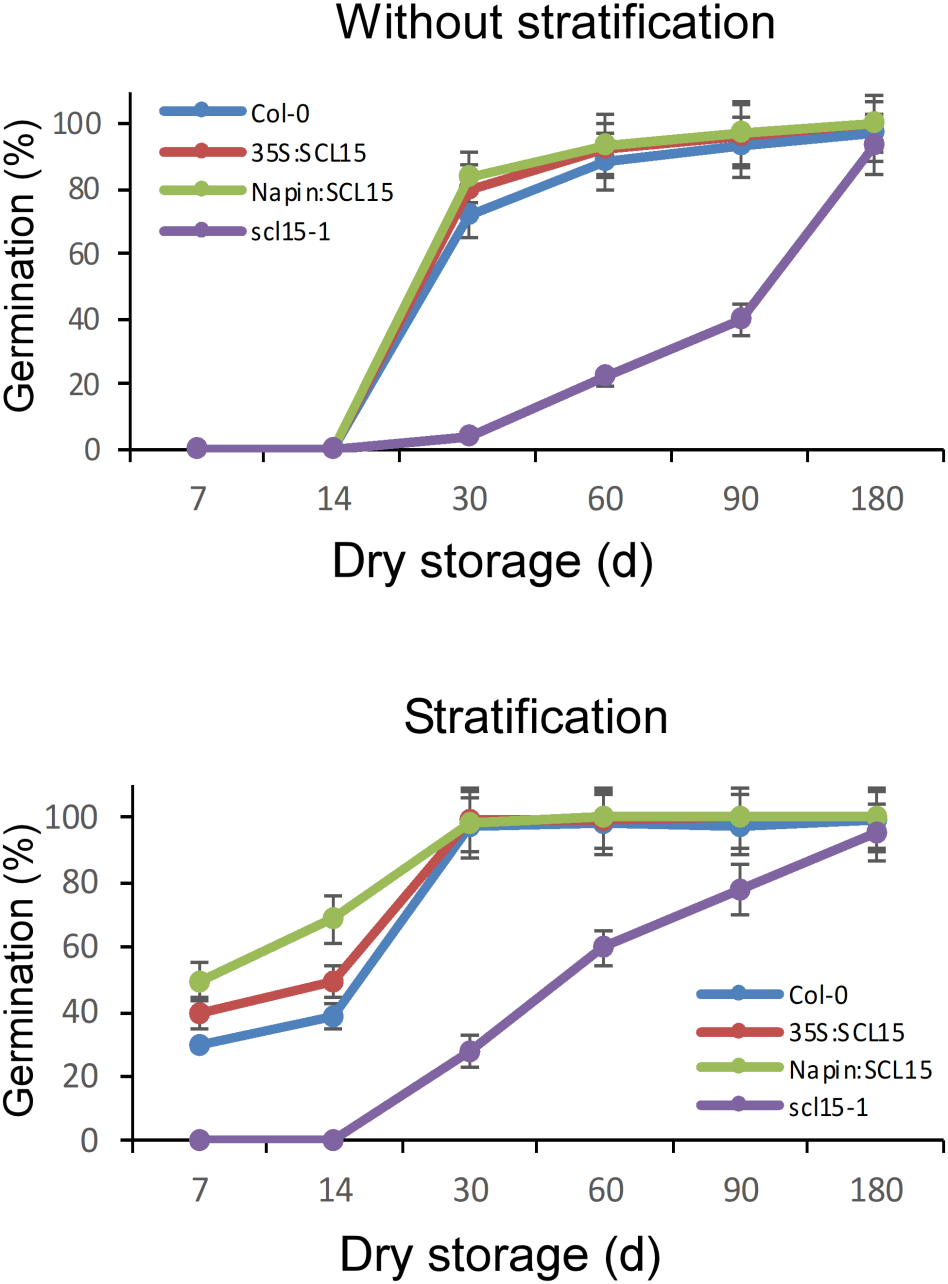
Germination of after‐ripened *scl15‐1* and SCL15‐overexpression lines. Seeds of wild‐type Col‐0, the *scl15‐1* mutant and SCL15 overexpression lines (Napin:SCL15 and 35S:SCL15) were harvested 25 days after flowering from mature dry siliques and subsequently stored dry (after‐ripened) for up to 6 months. Following stratification at 4°C for 3 days, seeds were sown on ½ MS medium. Germination percentages represent the mean (±SD) from at least three biological replicates.

Germination assays were conducted on wild‐type Col‐0, the *SCL15* mutant *scl15‐1*, a constitutive expression line in which the *SCL15* open reading frame was driven by the *CaMV 35S* promoter (35S:SCL15) and a seed‐specific expression line in which *SCL15* was driven by the *Napin* promoter (Napin:SCL15) to investigate whether regulation of seed germination by *SCL15* is associated with dormancy release. qRT‐PCR was performed to validate *SCL15* mRNA abundance. No *SCL15* transcript was detected in leaves or developing siliques of *scl15‐1* plants. In contrast, *SCL15* was expressed in leaves and siliques of 35S:SCL15 plants and was highly expressed in maturing siliques of Napin:SCL15 plants (Figure S1). Dormancy of Col‐0, Napin:SCL15 and 35S:SCL15 lines was largely released after 30 days of dry storage, with germination rates exceeding 70%. In comparison, germination of *scl15‐1* was less than 5% after 30 days, with complete dormancy release occurring after 6 months of dry storage (Figure 1). Dormancy release in Col‐0, 35S:SCL15 and Napin:SCL15 lines was fully completed when seeds were stratified after dry storage for 30 days, whereas germination of *scl15‐1* was less than 30% under these conditions. Germination of *scl15‐1* was less affected by the stratification treatment as dormancy did not break after 14 days of dry storage, whereas the germination rates for Napin:SCL15, 35S:SCL15 and Col‐0 were 68%, 49% and 38%, respectively, under the same conditions. This indicates that *scl15‐1* has deeper seed dormancy than wild‐type Col‐0 and that expression of *SCL15* in the seed reduced primary dormancy. However, the sensitivity of *scl15‐1* seeds to stratification was enhanced after dry storage for more than 30 days, implying that seed dormancy in *scl15‐1* is mainly controlled by after‐ripening (Figure 1). These data demonstrate that dormancy release was inhibited by the mutation of *SCL15* and promoted by the expression of *SCL15* in the seed.

### 3.2 Global Transcriptomic Analysis Identifies Critical Biological Functions for SCL15

**FIGURE 2:**
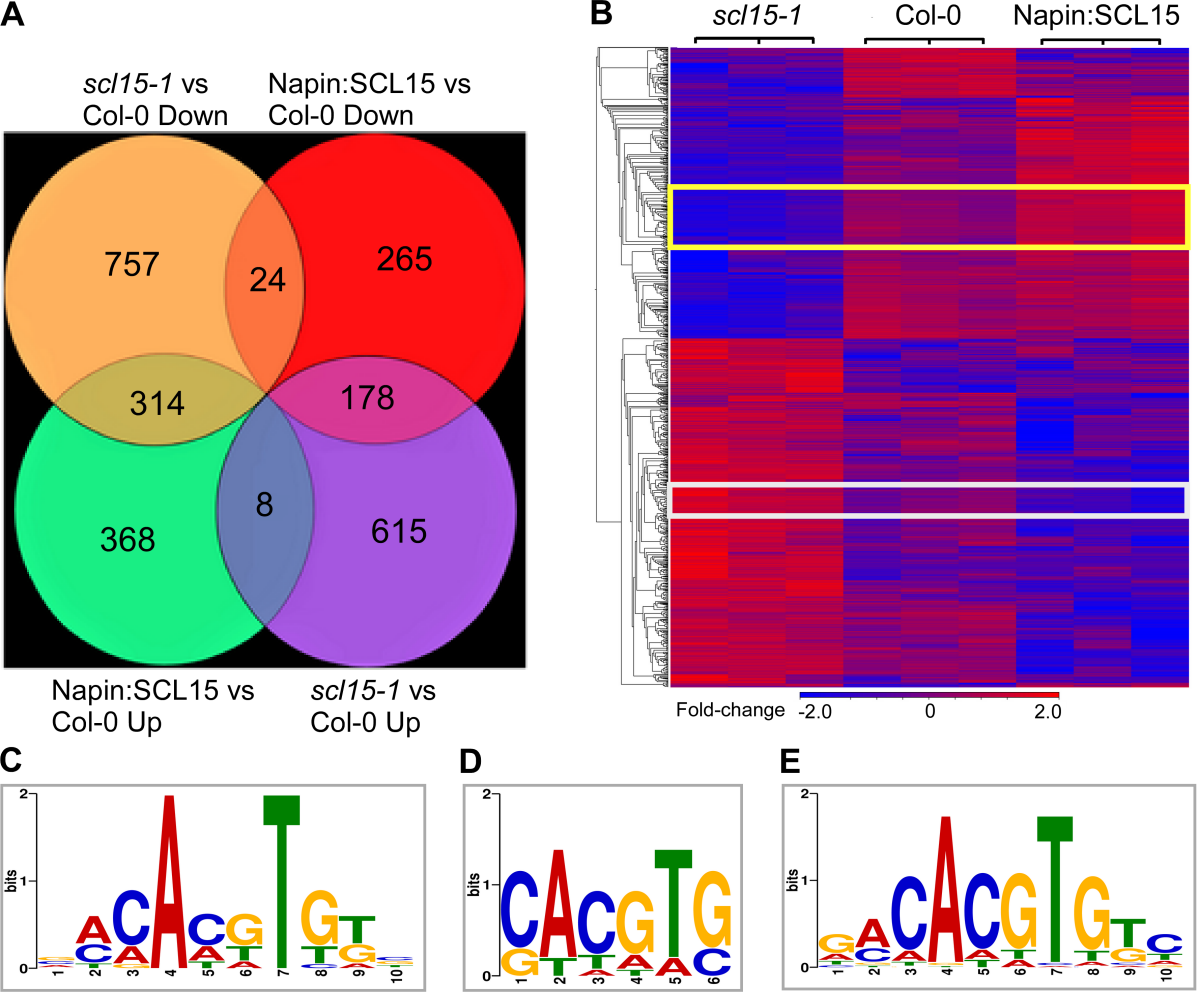
Global gene expression profile in seeds of *scl15‐1* and the seed‐specific Napin:SCL15 line expressing *SCL15* during the mid to late stages of seed development. (A) Venn diagram showing the number and distribution of differentially expressed genes across the comparisons of wild type Col‐0, *scl15‐1* and Napin:SCL15. (B) Heat map showing the expression profile of all differentially expressed genes that appeared in at least one statistical comparison. Mutant *scl15‐1* or the Napin:SCL15 line were compared with wild type Col‐0 (*scl15‐1* vs. Col‐0; Napin:SCL15 vs. Col‐0). Blue indicates downregulated values, red indicates upregulated values. The yellow rectangles indicate genes that were downregulated in *scl15‐1* (*scl15‐1* vs. Col‐0 Down), but upregulated in Napin:SCL15 (Napin:SCL15 vs. Col‐0 Up), most of which were associated with seed dormancy, abiotic stimulus particularly temperature response. The white rectangles indicate the genes that were upregulated in *scl15‐1* (*scl15‐1* vs. Col‐0 Up), but downregulated in Napin:SCL15 (Napin:SCL15 vs. Col‐0 Down). (C) The most represented motif (motif D) present in the regulatory regions for the genes that were downregulated in *scl15‐1* compared with Col‐0. (D) Motif U is present in the regulatory regions of genes that were upregulated in *scl15‐1* compared with Col‐0. (E) The most represented DNA binding motif (motif DU) present in the regulatory regions of the genes that were downregulated in *scl15‐1*, but upregulated in Napin:SCL15.

Gene expression analysis was conducted on siliques at the mid‐to‐late stage of seed development. Genes that were expressed in silique valves rather than maturing seed were filtered out to examine variance in mRNA transcript abundance between Col‐0, *scl15‐1* and Napin:SCL15 lines. An overview of the sequencing results and the distribution of distinct clean reads after low‐quality data were removed is shown in Table S2. Principal component analysis showed that the biological replicates clustered together and that the three lines had distinct gene expression profiles (Figure S2). qRT‐PCR analysis was conducted to confirm the expression patterns of the Differentially Expressed Genes (DEGs) identified from the RNA‐seq study (Figure S3). DEGs that were affected by either *SCL15* mRNA abundance or seed‐specific *SCL15* expression were identified. Among these, 178 genes were upregulated in *scl15‐1*, but downregulated in the Napin:SCL15 line, while 314 genes were downregulated in *scl15‐1*, but upregulated in the Napin:SCL15 line (Figure 2A,B).

The promoter sequences associated with the DEGs were analysed for enriched DNA‐binding motifs. The most over‐represented motif for the 757 genes that were downregulated in *scl15‐1* versus Col‐0 was designated motif D (*E*‐value: 3.4e‐006) (Figure 2C). The most significant Gene Ontology (GO) terms associated with motif D include ABA and auxin response, dehydration and light response (Table S3). Motif comparison analysis was conducted to identify proteins that potentially target motif D, and two groups of TFs were identified: basic leucine zipper (bZIP) (e.g., ABI5) and basic helix–loop–helix (bHLH) regulators (e.g., PIF6). The most enriched DNA‐binding motif for the 314 genes that were downregulated in *scl15‐1* and upregulated in Napin:SCL15 was designated motif DU (*E*‐value: 1.4e‐006) (Figure 2E). GO term association analysis with the motif DU targets revealed enriched GO terms, including response to ABA and light (Table S4). Motif comparison analysis revealed 29 TFs that could bind to motif DU, most of which belong to the bZIP and bHLH families. The most represented motif for the 615 genes that were upregulated in *scl15‐1* versus Col‐0 was designated motif U (*E*‐value: 5.4e‐006), with a consensus sequence of CACGTG (Figure 2D), which is also the core sequence of motif D and motif DU. All three DNA‐binding motifs share the same consensus palindromic G‐box (CACGTG) or E‐box (CANNTG), which is recognized by TFs from the bHLH and bZIP families (Ezer et al. 2017).

**FIGURE 3:**
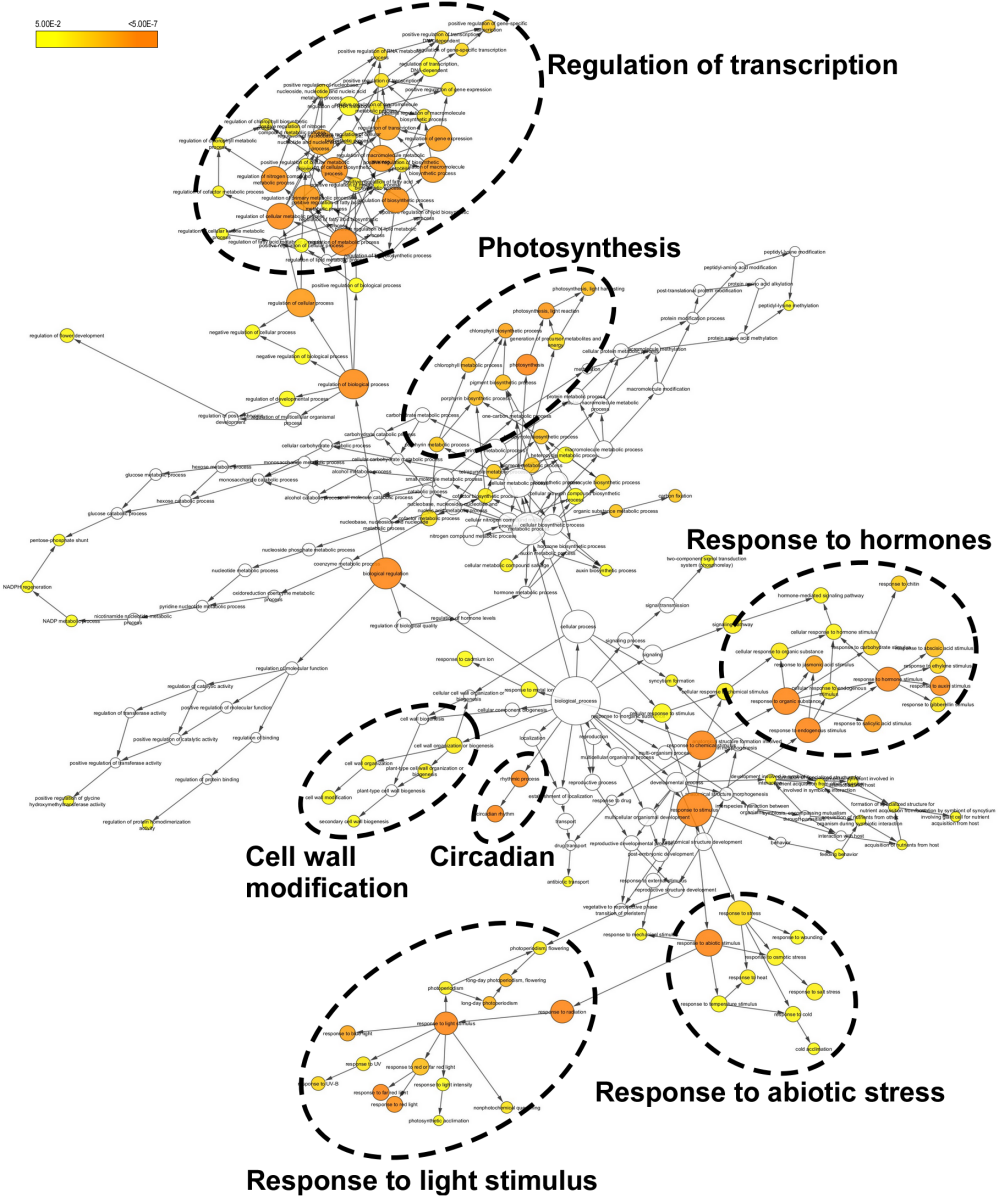
BiNGO analysis showing over‐represented categories within the biological process ontology among the 178 genes that were more than 1.5‐fold (*p* < 0.05 from three biological replicates with a Benjamini‐Hochberg FDR of 0.05) upregulated in *scl15‐1* mutant, but downregulated in Napin:SCL15 plants compared with the wild type Col‐0. The most over‐represented GO categories are shown. Coloured nodes ranging from yellow to dark orange represent the levels of significance of the over‐represented GO terms with *p* values from 5E‐2 to 5E‐7.

**FIGURE 4:**
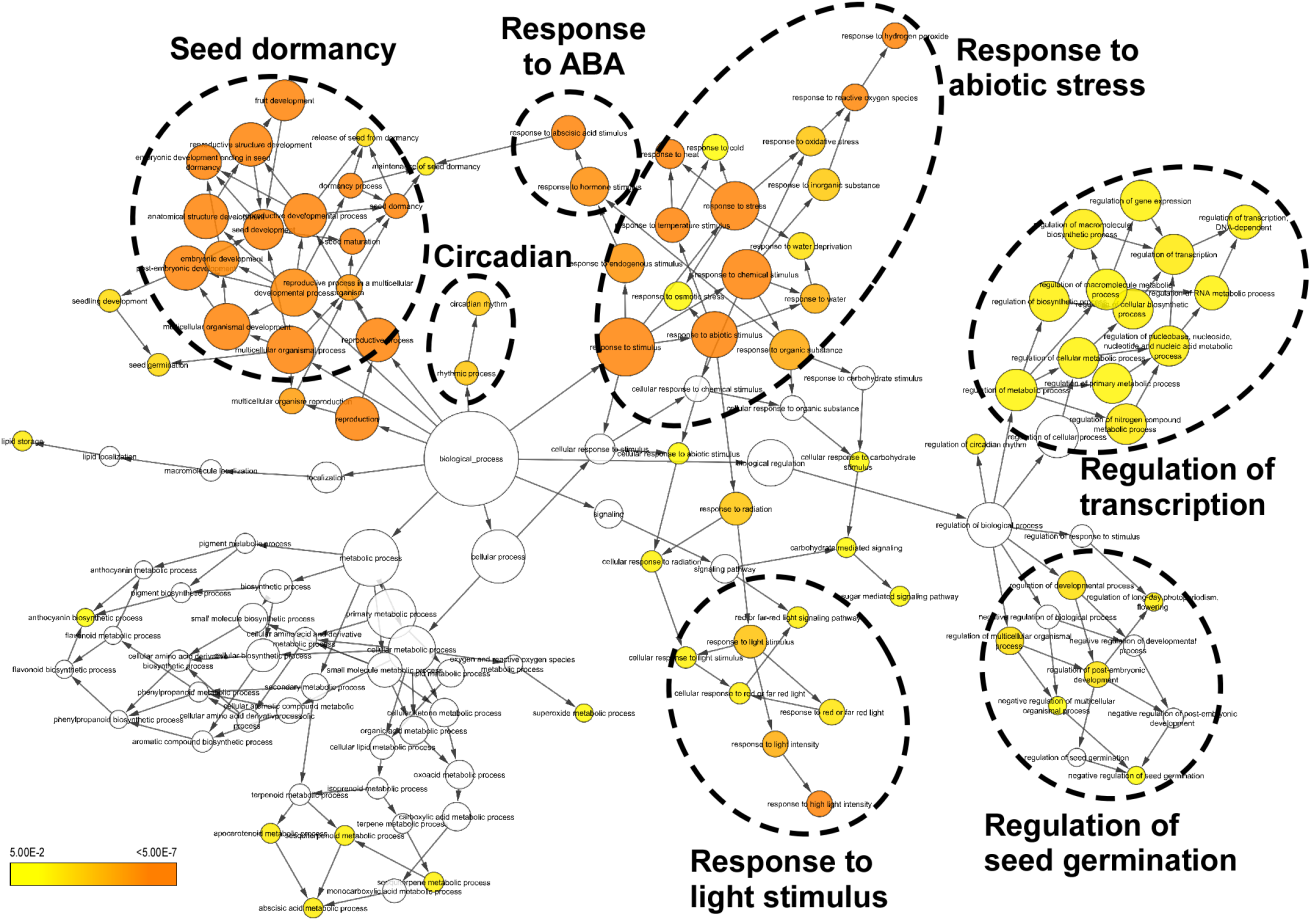
BiNGO analysis representing over‐represented categories of the ontology biological process among the 314 genes that were more than 1.5‐fold (*p* < 0.05 from three biological replicates with a Benjamini‐Hochberg FDR of 0.05) downregulated in *scl15‐1*, but upregulated in Napin:SCL15 plants compared to the wild type Col‐0. The most over‐represented GO categories are shown. Coloured nodes ranging from yellow to dark orange represent the levels of significance of the over‐represented GO terms with *p* values from 5E‐2 to 5E‐7.

The potential biological functions of the subsets of DEGs mentioned above were evaluated by GO analysis using both BiNGO and agriGO v2.0 to elucidate the contribution of SCL15 to seed dormancy and germination. The most represented GO categories in the biological process classification were circadian clock, CW modification, photosynthesis, and response to abiotic stress, light stimulus and response to hormones (mainly auxin and ABA) for the 178 seed genes that were upregulated in *scl15‐1,* but downregulated in Napin:SCL15 (Figure 3; Table S5). The most represented GO categories include seed dormancy, response to ABA, response to abiotic stress, response to light and circadian clock and regulation of seed germination for the 314 genes that were downregulated in *scl15‐1,*  but upregulated in Napin:SCL15 (Figure 4; Table S6). By comparing the two groups of genes affected by SCL15, it was found that most genes involved in the pathways for light response, circadian clock and abiotic stress response were either positively or negatively regulated by SCL15 in maturing seeds. In contrast, most genes controlling auxin metabolism, auxin signalling and CW modification were negatively regulated, while most genes involved in seed dormancy, ABA metabolism, ABA signalling and regulation of germination were positively regulated by SCL15. This analysis suggests that SCL15 plays a role in the regulation of seed dormancy release associated with the circadian clock, hormone signalling (mainly ABA and auxin) and CW organization or biogenesis.

### 3.3 *SCL15* Mutation Leads to Changes in Expression of Endosperm Wall Remodelling Genes

**FIGURE 5:**
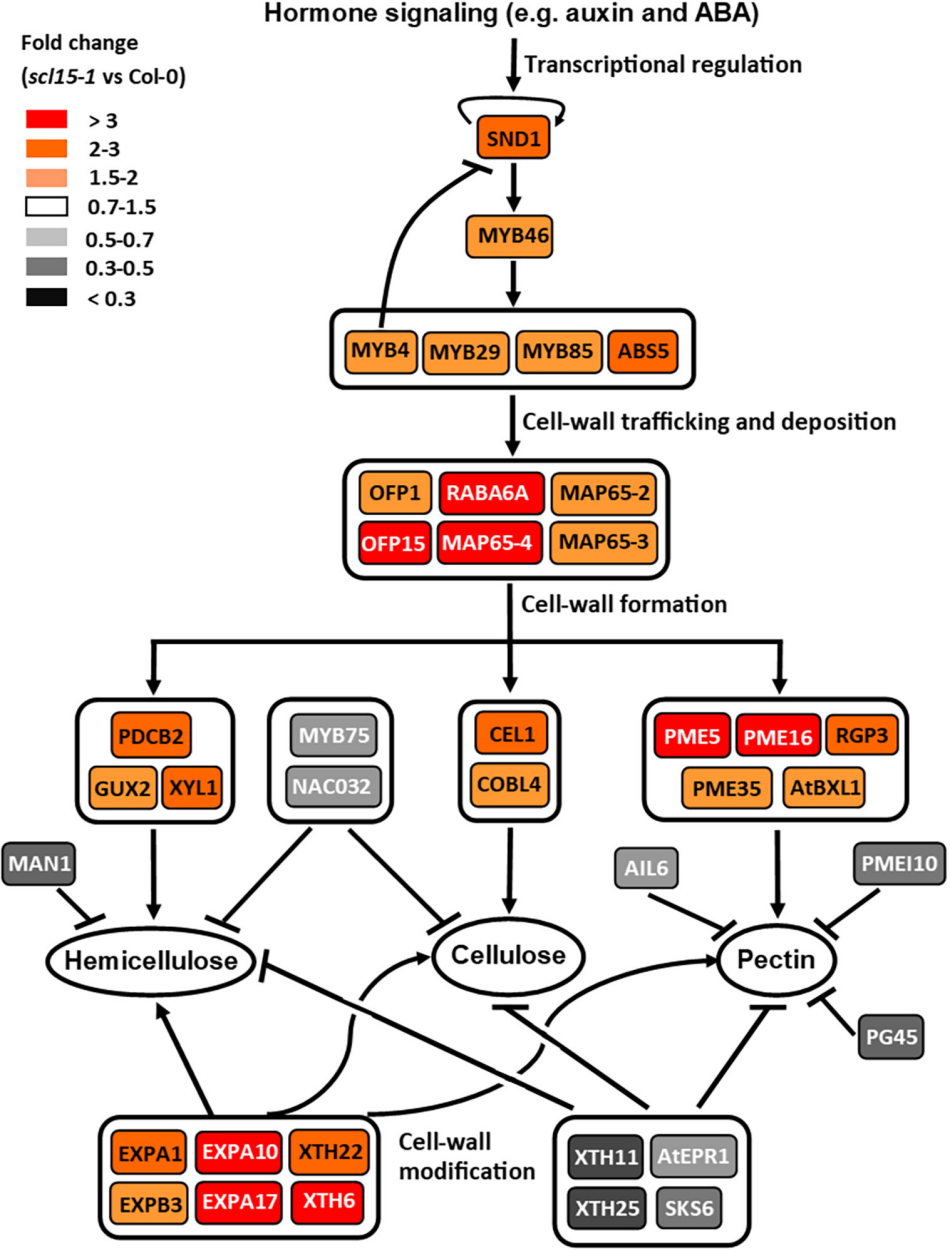
Changes in expression levels of genes involved in cell wall biosynthesis in maturing Arabidopsis seeds after *SCL15* mutation. Cell wall biosynthesis is regulated by transcriptional networks with SND1 at the top level, MYB46 at the second level and other regulators, such as MYB85, at the third level. Pectin and hemicelluloses are synthesized, transported and modified by proteins, such as MAP65‐2. Pectic HGs are selectively demethylesterified via the wall‐bound PMEs, such as PME35. Arabinan‐rich rhamnogalacturonan‐I (RG‐I) is metabolized by enzymes, such as endosperm‐specific RGP3. Biosynthesis and maturation of hemicellulose XyG are catalysed by enzymes, such as α‐xylosidase XYL1. The hemicelluloses xylan and callose are modified by GUX2 and PCDB2, respectively. For wall polymer catabolism, mannan‐rich hemicelluloses are degraded by MAN1 and demethylesterified HGs are degraded by PGs, such as PG45. Cellulose accumulation and thickness are promoted by endo‐1,4‐beta‐glucanase CEL1 and phytochelatin synthetase‐like protein COBL4. CW polymer reinforcement is enhanced by CW‐remodelling proteins, such as XTH22, and weakened by proteins, such as AtEPR1. Arrows and bars indicate positive and negative regulation, respectively. Outline of the gene regulatory networks for CW formation is modified from Zhu and Li (2021). ABS5, ABNORMAL SHOOT 5; AIL6, AINTEGUMENTA‐LIKE 6; ADPG2, ARABIDOPSIS DEHISCENCE ZONE POLYGALACTURONASE 2; ATBXL1, ARABIDOPSIS BETA‐XYLOSIDASE 1; ATEPR1, ARABIDOPSIS EXTENSIN PROLINE‐RICH 1. CEL1, CELLULASE 1; COBL4, COBRA‐LIKE4; EXPA, ALPHA‐EXPANSIN; EXPB, BETA‐EXPANSIN; GUX2, GLUCURONIC ACID SUBSTITUTION OF XYLAN 2; MAN1, ENDO‐Β‐MANNANASE; MAP, MICROTUBULE‐ASSOCIATED PROTEIN; MYB, MYB DOMAIN PROTEIN; NAC032, NAC DOMAIN CONTAINING PROTEIN 32; OFP, OVATE FAMILY PROTEIN; PDCB2, PLASMODESMATA CALLOSE‐BINDING PROTEIN 2; PG45, POLYGALACTURONASE 45; PME, PECTIN METHYLESTERASE; PMEI10, PME INHIBITOR 10; RGP3, REVERSIBLY GLYCOSYLATED POLYPEPTIDE 3; SKS6, RABA6A, RAB GTPASE HOMOLOG A6A; SKEWED5 (SKU5)‐SIMILAR 6; SND1, SECONDARY WALL‐ASSOCIATED NAC DOMAIN 1; XTH, XYLOGLUCAN ENDOTRANSGLYCOSYLASE/HYDROLASE; XYL1, ALPHA‐XYLOSIDASE 1.

Endosperm weakening, accompanied by ME wall loosening and radicle elongation, is a prerequisite for endosperm rupture and the completion of seed germination (Bewley 1997; Müller et al. 2006; Sechet et al. 2016; Steinbrecher and Leubner‐Metzger 2017; Nonogaki 2019; Holloway et al. 2021; Chandrasekaran et al. 2022). The DEGs associated with both transcript abundance and seed‐specific expression of SCL15 were analysed to investigate whether SCL15 regulates dormancy release and germination by modulating endosperm wall biomechanics. More than 40 genes were identified to be involved in CW biosynthesis, regulation and modification, among which a number of key TFs for wall strengthening were repressed by SCL15 in maturing seeds (Figure 5; Table S7). The biosynthesis of both primary and secondary CWs is regulated by a transcriptional network that is hierarchically organized and encompasses secondary wall NAC and MYB TFs (Liu, Yu, et al. 2021; Zhu and Li 2021; Fuertes‐Aguilar and Matilla 2024). Genes encoding the master regulators of cell wall formation and thickening [SECONDARY WALL‐ASSOCIATED NAC DOMAIN 1 (SND1/NST3) and MYB DOMAIN PROTEIN 46 (MYB46)], as well as their downstream regulators (MYB4 and MYB29), were all upregulated in *scl15‐1* and downregulated in Napin:SCL15.

The Arabidopsis endosperm CW is mainly composed of hemicellulose XyG, demethylesterified HGs and arabinan‐rich RG‐I (Lee et al. 2012). The fine‐tuning of XyG hydrolysis in CWs is essential for the control of ME rupture in response to exogenous and endogenous signals (Sechet et al. 2016; Shigeyama et al. 2016). The current study revealed that SCL15 plays a negative role in the expression of a subset of genes essential for the reinforcement of endosperm wall biomechanics. For example, *XYL1*, which encodes a XyG modifying enzyme and is a positive regulator for endosperm wall stiffness and dormancy maintenance (Nonogaki 2019), *GLUCURONIC ACID SUBSTITUTION OF XYLAN 2* (*GUX2*), which encodes a xylan modifying enzyme involved in secondary wall strengthening (Lee et al. 2012) and three pectin methylesterase (PME) genes (*PME5*, *PME16* and *PME35*), which are positive regulators for wall stiffening (Nonogaki 2019; Jobert et al. 2022), were all negatively regulated by SCL15 in maturing seeds. Additionally, *XTH22*, which encodes the endosperm‐abundant xyloglucan endotransglycosylase/hydrolase (XTH), and two EXP genes (*EXPA1* and *EXPA10*) (Antosiewicz et al. 1997; Samalova et al. 2020), were also negatively regulated by SCL15. XYL1 is an alpha‐xylosidase located in the endosperm and generates hemicellulose modifications essential for wall stiffness and dormancy maintenance (Shigeyama et al. 2016; Sechet et al. 2016; Nonogaki 2019). EXPA1 and XTH22 are abundant in the endosperm and positively regulate wall strengthening by modifying XyG during seed maturation (Antosiewicz et al. 1997; Samalova et al. 2020). PME5 and PME35 positively affect CW stiffening through HG demethylesterification (Hongo et al. 2012; Peaucelle et al. 2015).

Dormancy association analysis for SCL15‐repressed, wall‐strengthening genes provided further evidence for their role in dormancy maintenance (Figure S4A). In contrast, genes that were positively regulated by SCL15 (Figure 5) have a positive role in the initiation of germination. For example, the gene encoding the endosperm‐expressed, extension‐like protein ARABIDOPSIS EXTENSIN PROLINE‐RICH 1 (AtEPR1) and three genes encoding wall‐degrading enzymes, the seed‐specific ENDO‐BETA‐MANNANASE (MAN) 1 (AtMan1) (Figure S5), POLYGALACTURONASE 45 (PG45) and ARABIDOPSIS DEHISCENCE ZONE POLYGALACTURONASE 2 (ADPG2), are all involved in wall weakening (Figure S4B). PMEs are most active at alkaline pH (Senechal et al. 2015) and the upregulation of the genes encoding them in *scl15‐1* seeds suggests an alkaline pH environment in the endosperm walls, which would promote endosperm CW stiffening in *scl15‐1* and enhance dormancy. An alkaline pH is also not favourable for the activity of wall‐degrading enzymes required for germination, such as AtMAN1 and PG45, which have optimal activity at pH 4–5 (Wang et al. 2014; Xiao et al. 2017). The local acidic environment, associated with elevated auxin levels and the so‐called acid growth in plants (Hocq et al. 2017; Arsuffi and Braybrook 2018; Lin et al. 2021), is discussed further below in relation to SCL15.

### 3.4 *SCL15* Mutation Affects the Expression of Critical Genes in ABA Metabolism and Signalling

**FIGURE 6:**
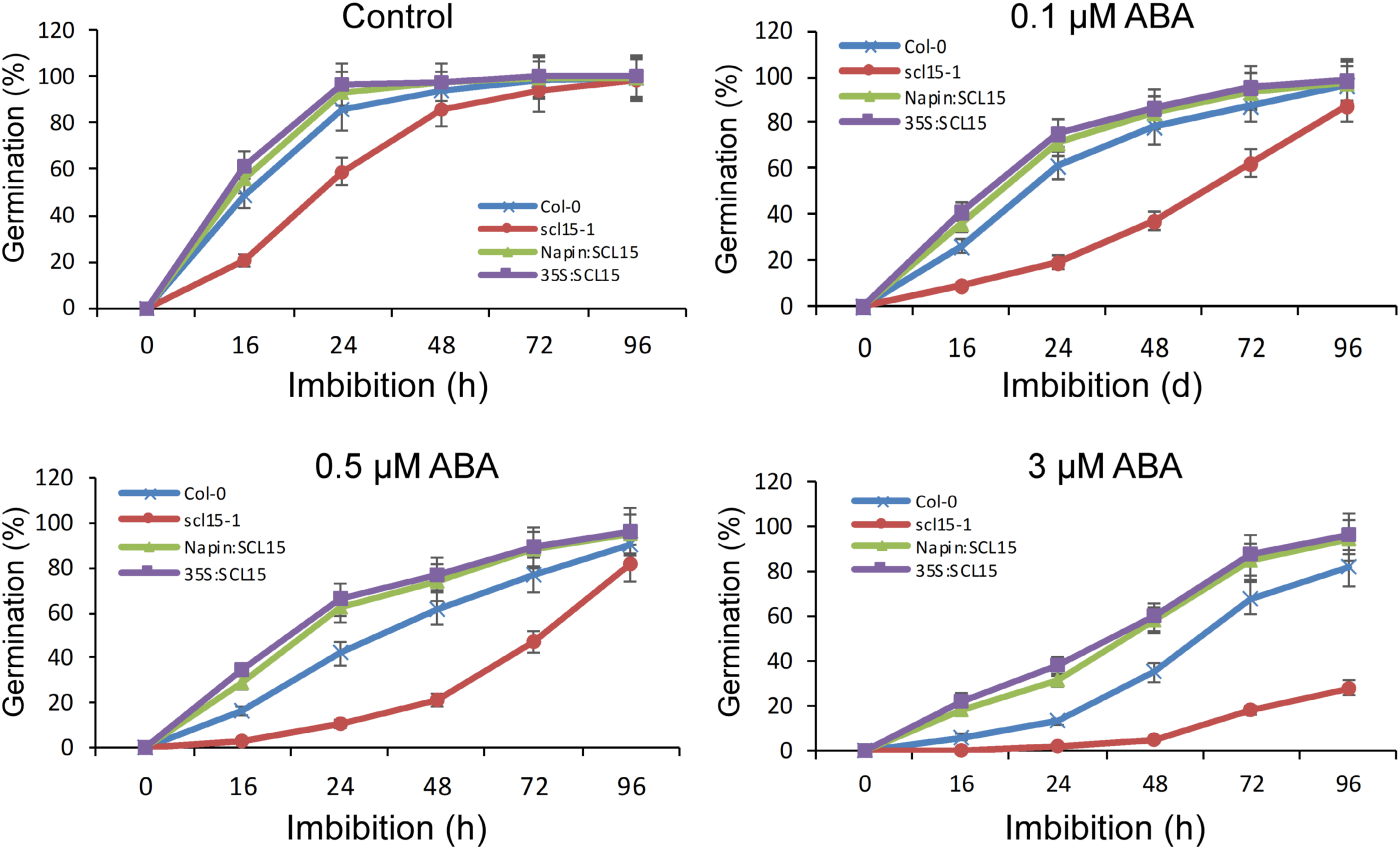
Germination behaviour of *scl15‐1* and *SCL15*‐overexpression lines in response to ABA. Arabidopsis seeds that were partially after‐ripened for 3 months were stratified at 4°C for 3 days and then sown on ½ MS medium. Germination percentages represent the mean (±SD) from at least three biological replicates.

**FIGURE 7:**
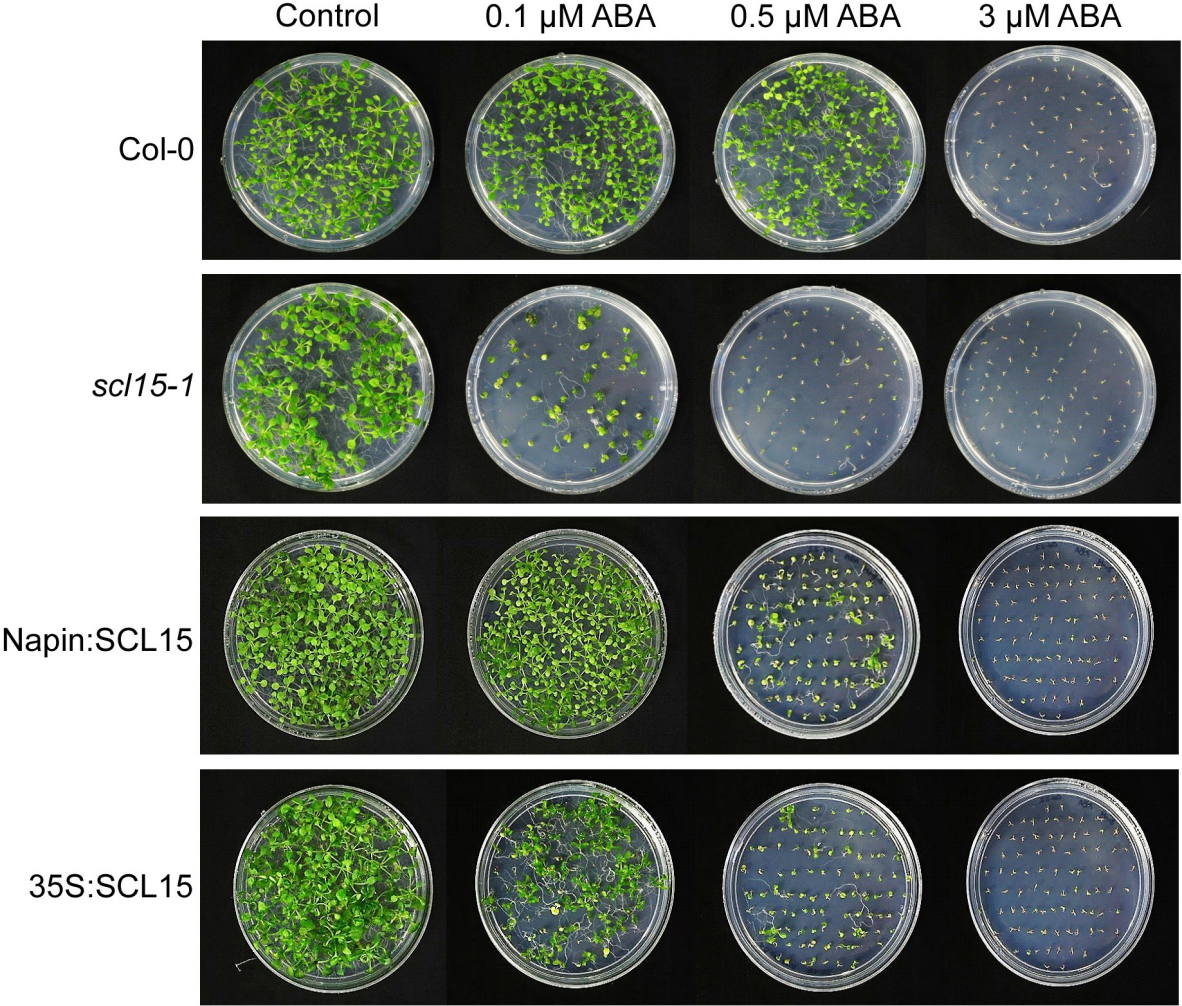
Post‐germination growth of *scl15‐1* and *SCL15*‐overexpression lines and their response to ABA. Seeds of *scl15‐1*, Napin:SCL15, 35S:SCL15 and wild type Col‐0 were stratified for 3 days at 4°C and sown onto ½ MS agar medium supplemented with 0, 0.1, 0.5 or 3 μm ABA. Representative images were shown as the morphology of seedlings 14 days after imbibition. It is noted that *scl15‐1* mutation increases the inhibition of ABA to germination and post‐germination growth.

ABA plays a critical role in the induction and maintenance of seed dormancy (Shu et al. 2016; Nonogaki 2019; Ali et al. 2021). Germination assays were conducted in the presence or absence of ABA to investigate whether SCL15 regulation of dormancy release is associated with the ABA response (Figure 6). *scl15‐1* germination was markedly inhibited at ABA concentrations as low as 0.1 μM; at this concentration, the germination rate of *scl15‐1* was reduced by 68% after imbibition for 24 h when compared with the control, whereas wild‐type Col‐0 germination was reduced only by 29%. Post‐germination growth and seedling establishment for *scl15‐1* was also severely inhibited at 0.1 μM ABA, and it did not grow at all in the presence of 0.5 μM ABA (Figure 7). The germination and post‐germination growth of the Napin:SCL15 and 35S:SCL15 expression lines were similar to wild‐type Col‐0 in the presence of 0.1 μM ABA. However, the post‐germination growth of both Napin:SCL15 and 35S:SCL15 lines was dramatically reduced at an ABA concentration of 0.5 μM (Figure 7). The sensitivity of *scl15‐1* to ABA during germination was further examined using the ABA biosynthesis inhibitor nordihydroguaiaretic acid (NDGA). NDGA significantly increased *scl15‐1* germination compared to the control after 24 h and 48 h of imbibition, whereas no significant difference was observed in wild‐type Col‐0 or the Napin:SCL15 and 35S:SCL15 expression lines (Figure S6). These results indicate that SCL15 negatively regulates primary seed dormancy, at least in part, through interaction with ABA signalling.

**FIGURE 8:**
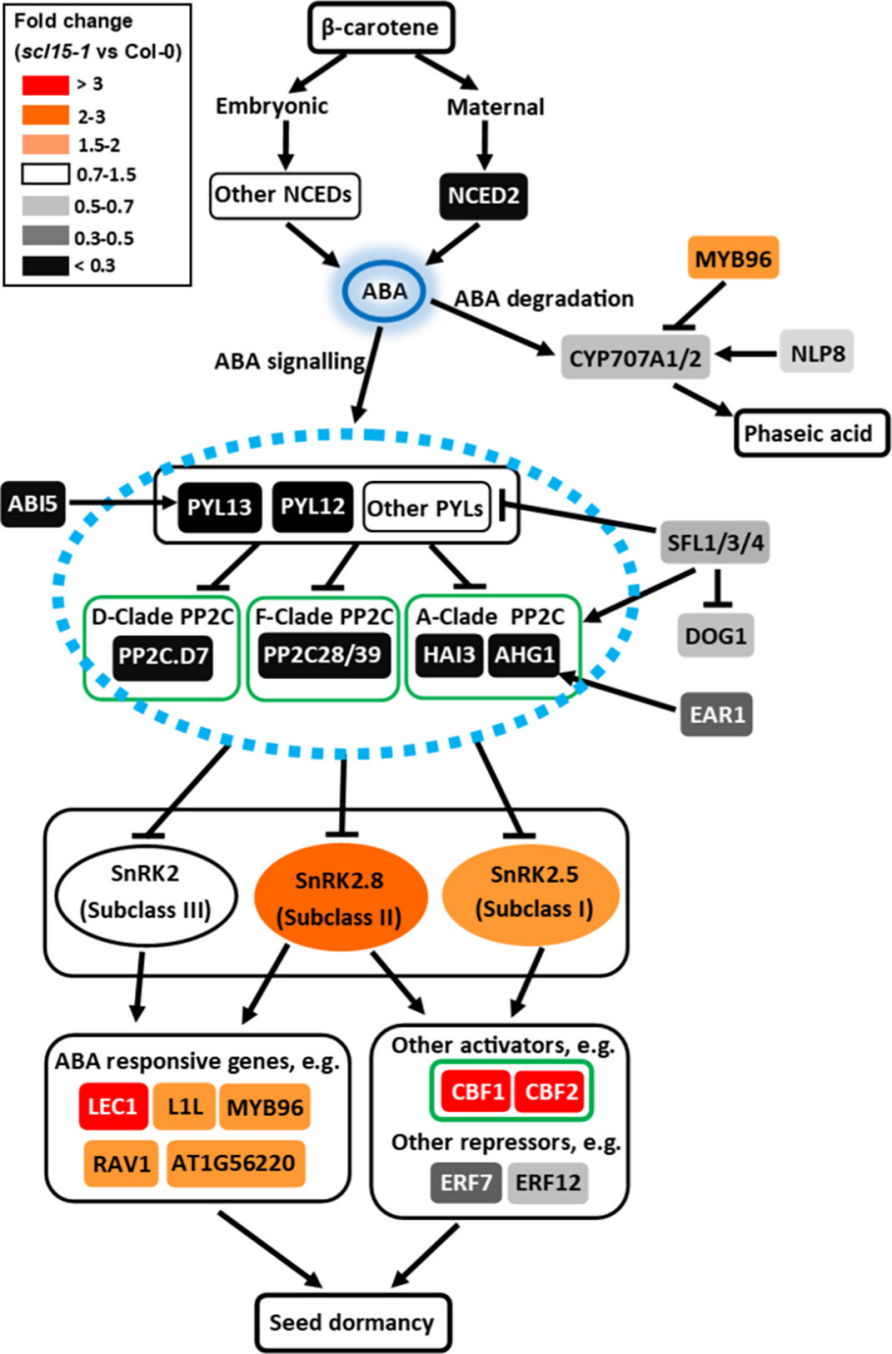
Changes in expression of ABA‐responsive genes in maturing Arabidopsis seeds after *SCL15* mutation. Maternal ABA is derived from NCED2 and NCED3, and embryonic ABA is derived from other NCEDs. The 8ʹ‐hydroxylation by CYP707A enzymes belong to the predominant pathway for ABA catabolism; they catalyse the hydroxylation of ABA to form phaseic acid. PYL, PP2C and SnRK2 form a major ABA signalling complex. The suppression of A‐ and/or D‐clade PP2Cs establishes the dormant state of seeds. Dormancy repressor SLF1 positively regulates A/D‐clade PP2Cs and is epistatic to the regulator of dormancy DOG1. SnRK2 Subclasses I and II are released from A‐and/or D‐clade PP2C‐dependent regulation and activated to regulate seed dormancy by regulation of downstream factors (e.g., ABA sensitivity promoter MYB96). More details are in the text of this article. Outline of ABA biosynthesis and signalling pathways is derived from Chen, Li, et al. (2020). ABI5, ABA INSENSITIVE 5; CWRP, cell‐wall remodelling proteins; CYP, CYTOCHROME P450; DOG1, DELAY OF GERMINATION1; EAR1, ENHANCER OF ABA CO‐RECEPTOR1; ERF, ETHYLENE RESPONSE FACTOR. LEC1, LEAFY COTYLEDON1; L1L, LEC1‐LIKE; NCED, 9‐*CIS*‐EPOXYCAROTENOID DIOXYGENASES; NLP8, NIN‐LIKE PROTEIN 8; PUB, plant U‐box; PYL, PYR1‐LIKE; PYR1, PYRABACTIN RESISTANCE1; RAV1, RELATED TO ABI3/VP1; RCAR, REGULATORY COMPONENTS OF ABA RECEPTORS; SFL, SEED DORMANCY FOUR‐LIKE; SnRK2, SNF1‐RELATED PROTEIN KINASES 2. Other abbreviations are in the legends to Figures 9 and 10.

**FIGURE 9:**
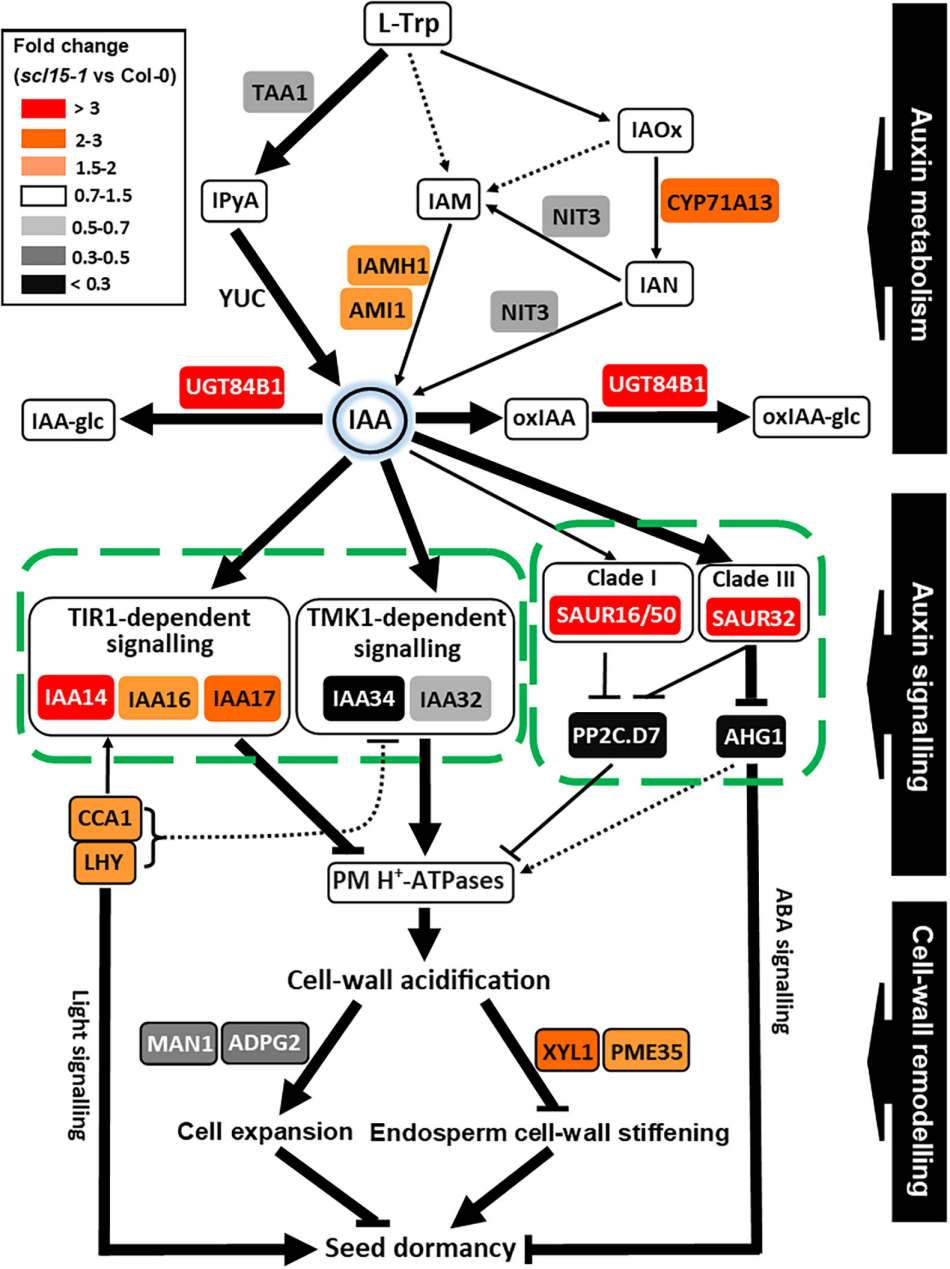
Changes in expression levels of auxin‐responsive genes in maturing Arabidopsis seeds after *SCL15* mutation, showing auxin‐regulated, CW remodelling and dormancy maintenance associated with ABA and clock signalling. Auxin biosynthetic pathways include the major IPyA pathway, the additional IAM pathway and the *Brassica*‐specific IAOx pathway. For the inactivation of IAA, UGT84B1 catalyses the formation of IAA conjugates with sugars, such as IAA‐glc conjugate. IAA is also inactivated by irreversible oxidation to oxIAA, followed by glycosylation to form oxIAA‐glc, which is also catalysed by UGT84B1. PM H^+^‐ATPases are regulated by two distinct, antagonistically‐acting, signalling pathways; the intracellular TIR1‐dependent canonical auxin signalling pathway with components of canonical AUX/IAA proteins, such as IAA16, and the cell surface‐based TMK1‐dependent noncanonical pathway with components of noncanonical AUX/IAA proteins IAA32/34. PM H^+^‐ATPase activity is also regulated by Clade I (e.g., SAUR16) and Clade III SAURs (SAUR32). Enhanced cell‐wall acidification is usually associated with cell expansion and reduced cell‐wall stiffening. SAUR32 promotes seed dormancy by suppression of Clade A PP2C (e.g., AHG1). Key circadian clock regulators CCA1 and LHY positively regulate dormancy and interact with auxin signalling. Thick solid arrows/bars indicate main pathways in which the enzymes, genes or intermediates are known, and dotted arrows indicate pathways that are not well defined. Outline of the pathway for auxin biosynthesis is derived from Morffy and Strader (2020). ABA, ABSCISIC ACID; AHG1, ABA‐HYPERSENSITIVE GERMINATION; AMI1, IAM AMIDOHYDROLASE 1; ARR6, ARABIDOPSIS RESPONSE REGULATORS 6; CCA1, *CIRCADIAN CLOCK ASSOCIATED 1*; CK, CYTOKININ; CYP, CYTOCHROME P450; HAI1, HIGHLY ABA‐INDUCED PP2C GENE1; IAA, INDOLE‐3‐ACETIC ACID; IAA‐GLC, IAA‐GLUCOSE; IAM, INDOLE‐3‐ACETAMIDE; IAMH1, IAM HYDROLASE1; IAN, INDOLE‐3‐ACETONITRILE; IAOX, INDOLE ACETALDOXIME; IPYA, INDOLE‐3‐PYRUVIC ACID; *LHY, LATE ELONGATED HYPOCOTYL*; OXIAA, 2‐OXOINDOLE‐3‐ACETIC ACID; PM, PLASM MEMBRANE; PME35, PECTIN METHYLESTERASE 35; PP2C, PROTEIN PHOSPHATASE 2C; SAUR, SMALL AUXIN UP RNA; TAA1, TRYPTOPHAN AMINOTRANSFERASE 1; TIR1, TRANSPORT INHIBITOR RESPONSE 1; TMK1, TRANSMEMBRANE KINASE1; L‐TRP, L‐TRYPTOPHAN; UGT, URIDINEDIPHOSPHATE (UDP) GLYCOSYLTRANSFERASE (UGT); YUC, YUCCA. Other abbreviations are in the legend to Figure 5.

Mutation *of SCL15* not only promotes dormancy, but also markedly increases ABA sensitivity during germination and post‐germination growth. Consistent with the dormancy phenotypes for *scl15‐1*, a subset of genes essential for ABA metabolism and ABA signalling were found to be regulated by SCL15 (Figure 8; Table S8). For example, genes encoding the endosperm‐abundant ABA catabolic enzymes and negative regulators of dormancy, CYP707A1 and CYP707A2 (Okamoto et al. 2006), as well as NIN‐like protein 8 (NLP8), an activator of CYP707A2 and a master regulator of the primary nitrate response (Yan et al. 2016), were positively regulated by SCL15. Genes encoding the positive regulator of ABA‐mediated seed germination, PYRABACTIN RESISTANCE 1‐LIKE 12 (PYL12) (Zhao et al. 2020), the negative dormancy regulator of CLADE A PROTEIN PHOSPHATASE 2C (PP2C), ABA‐HYPERSENSITIVE GERMINATION 1 (AHG1) and its activator, ENHANCER OF ABA CO‐RECEPTOR1 (EAR1) (Okamoto et al. 2006; Yan et al. 2016; Nishimura et al. 2018), and a group of negative dormancy regulators, the SEED DORMANCY FOUR‐LIKE1 (SFL1) and homologues (Cao et al. 2020; Liu et al. 2020) (Figure S7), were also positively regulated by SCL15. In contrast, genes encoding three groups of positive regulators of seed dormancy were negatively regulated by SCL15, including those encoding endosperm‐abundant LAFL TF LEC1 and its paralog LEC1‐LIKE (L1L) (Figure S8), plant U‐box (PUB) E3 ligases (e.g., PUB18 and PUB23) (Zhao et al. 2017), and the SMALL AUXIN‐UPREGULATED RNAs (SAUR), SAUR16 and SAUR50 (Figures 8 and 9). These results suggest that besides ABA signalling, the nitrate response and ubiquitination pathways are likely involved in the contribution of SCL15 to primary seed dormancy.

A number of critical regulators for seed dormancy maintenance downstream of ABA signalling were also regulated by SCL15. For example, the expression of *LEC1*, a central regulator of seed development and maturation expressed exclusively in the endosperm of mature seeds (Song et al. 2021), was negatively regulated by SCL15 in maturing seeds. In contrast, the genes encoding the highly endosperm‐abundant SFL1 and its homologues SFL3/4, which act as negative dormancy regulators and repress embryonic gene expression during the seed‐to‐seedling transition, were positively regulated by SCL15 during seed maturation. SFL1 (Zheng et al. 2022), also referred to as *Arabidopsis thaliana* SEED DORMANCY 4‐LIKE (AtSDR4L) (Cao et al. 2020), Reversal of RDO5 (ODR1) (Liu et al. 2020) or ABA‐Induced Transcription Repressors Like (AITRL) (Ma et al. 2021), positively regulates genes encoding Clade A PP2C and negatively regulates those encoding intracellular PYL‐like ABA receptors (Ma et al. 2021), indicating that these factors play a negative role in seed dormancy. Interestingly, cross‐regulation occurs between SCL15 and LEC1 or SFL1, as evidenced by the downregulation of *SCL15* in *lec1‐1* (Pelletier et al. 2017) and *Atsdr4l* (Wu et al. 2022) mutants.

### 3.5 *SCL15* Mutation Affects the Expression of Genes Involved in Endosperm‐Localized Auxin Metabolism and Signalling

In addition to ABA, auxin plays an essential role in seed dormancy and germination (Shu et al. 2016; Blakeslee et al. 2019). The role of auxin in the germination of *scl15‐1* and *SCL15*‐expression lines was examined in the presence of 2,4‐D or the auxin transport inhibitor 2,3,5‐triiodobenzoic acid (TIBA). Compared with the control, the germination of Col‐0 was enhanced by 15% and 6% after treatment with 0.2 nM 2,4‐D for 24 h and 48 h, whereas the germination rate of *scl15‐1* increased by 70% and 48%, respectively. Germination of Col‐0 and the *SCL15*‐expression lines was not affected after imbibition for 96 h in the presence of TIBA, whereas germination of *scl15‐1* was reduced by 65% compared to the control (*p* < 0.01) (Figure S9). These results indicate that *SCL15* mutation increases sensitivity to auxin during germination.

Auxin levels are spatially regulated through coordinated biosynthesis, transport, storage, conjugation and degradation to optimize concentration‐dependent growth responses and adaptive responses to environmental cues (Blakeslee et al. 2019; Solanki and Shukla 2024). Seventeen genes that were regulated by *SCL15* are known to play a role in auxin metabolism and signalling (Table S9). The gene encoding the paternally expressed, endosperm‐imprinted TRYPTOPHAN AMINOTRANSFERASE 1 (TAA1) (Klosinska et al. 2016) was positively regulated by SCL15. As a member of the highly conserved indole‐3‐pyruvic acid (IPyA) pathway (Morffy and Strader 2020), downregulation of *TAA1* in *scl15‐1* and upregulation in Napin:SCL15 suggest a low level of IAA in the *scl15‐1* endosperm. Local auxin accumulation is also modulated by the metabolic inactivation of IAA. Glycosylation of both IAA and 2‐oxoindole‐3‐acetic acid (oxIAA) is catalysed by the endosperm‐expressed UDP‐glucosyltransferase UGT84B1 (Porco et al. 2016; Aoi et al. 2020; Mateo‐Bonmatí et al. 2021), which was repressed by SCL15, further suggesting reduced local IAA levels in the endosperm of *scl15‐1*.

Genes encoding a group of Clade I SAURs, including SAUR16/50, and Clade III member SAUR32, were repressed by SCL15 in maturing seeds. *SAUR16/50* are induced by auxin, but repressed by ABA, whereas *SAUR32* is induced by ABA, but repressed by auxin, and is a positive regulator for ABA sensitivity and dormancy (He et al. 2021; Figure 9). Moreover, a group of canonical Auxin/Indole‐3‐Acetic Acid (Aux/IAA) genes (e.g., *IAA14*, *IAA16* and *IAA17*) and two noncanonical Aux/IAA genes (*IAA32* and *IAA34*) were repressed and activated by SCL15, respectively. Therefore, SCL15 regulates auxin signalling through both the intracellular, canonical, TIR1‐dependent Aux/IAA pathway and the cell surface‐based, noncanonical, TMK1‐dependent pathway (Cao et al. 2019; Lin et al. 2021; Figure 9). These results suggest that SCL15 positively affects endosperm‐localized auxin metabolism and signalling during seed maturation.

A major function of auxin in plant growth and development is the regulation of cell expansion through control of CW apoplast acidification, known as the acid growth mechanism (Arsuffi and Braybrook 2018). Relying on both the cell surface‐based, TMK1‐mediated and the intracellular, TIR1‐dependent auxin‐signalling pathways, auxin activates the plasma membrane‐localized P‐type H^+^‐ATPase, resulting in apoplastic acidification (Cao et al. 2019; Lin et al. 2021). Repression of the TMK1 pathway and activation of the TIR1 pathway in *scl15‐1* seeds could lead to disruption of the plasma membrane (PM) H^+^‐ATPase proton pump across the PM and, thus, elevate the pH in the local endosperm wall apoplast. Moreover, CWRPs regulate wall biomechanical properties in a tissue/cell‐specific manner, and their activities are largely dependent on local auxin gradients and associated apoplastic pH (Chen, Jung, et al. 2020; Wang et al. 2020; Chandrasekaran et al. 2022). For example, PME activity is promoted in alkaline environments and tissues with low IAA levels (Wang et al. 2020; Qiu et al. 2021), conditions associated with CW stiffening (Hongo et al. 2012; Peaucelle et al. 2015; Phyo et al. 2017). On the other hand, the activity of MANs is favoured by acidic pH and elevated IAA levels (Chen, Li, et al. 2020), and is correlated with wall weakening (Peaucelle et al. 2015; Arsuffi and Braybrook 2018; Chen, Li, et al. 2020). Consistent with this notion, genes encoding positive regulators for CW stiffening, such as PME35, are negatively regulated by SCL15, whereas those encoding wall weakening proteins, such as AtMAN1, are positively regulated by SCL15. These results indicate that endosperm wall stiffening in *scl15‐1* is increased due to reduced local auxin levels and apoplastic alkalinization, which leads to inhibition of local cellular expansion and delayed endosperm rupture during germination.

### 3.6 The Central Circadian Oscillator Is Affected by Mutation of *SCL15*

**FIGURE 10:**
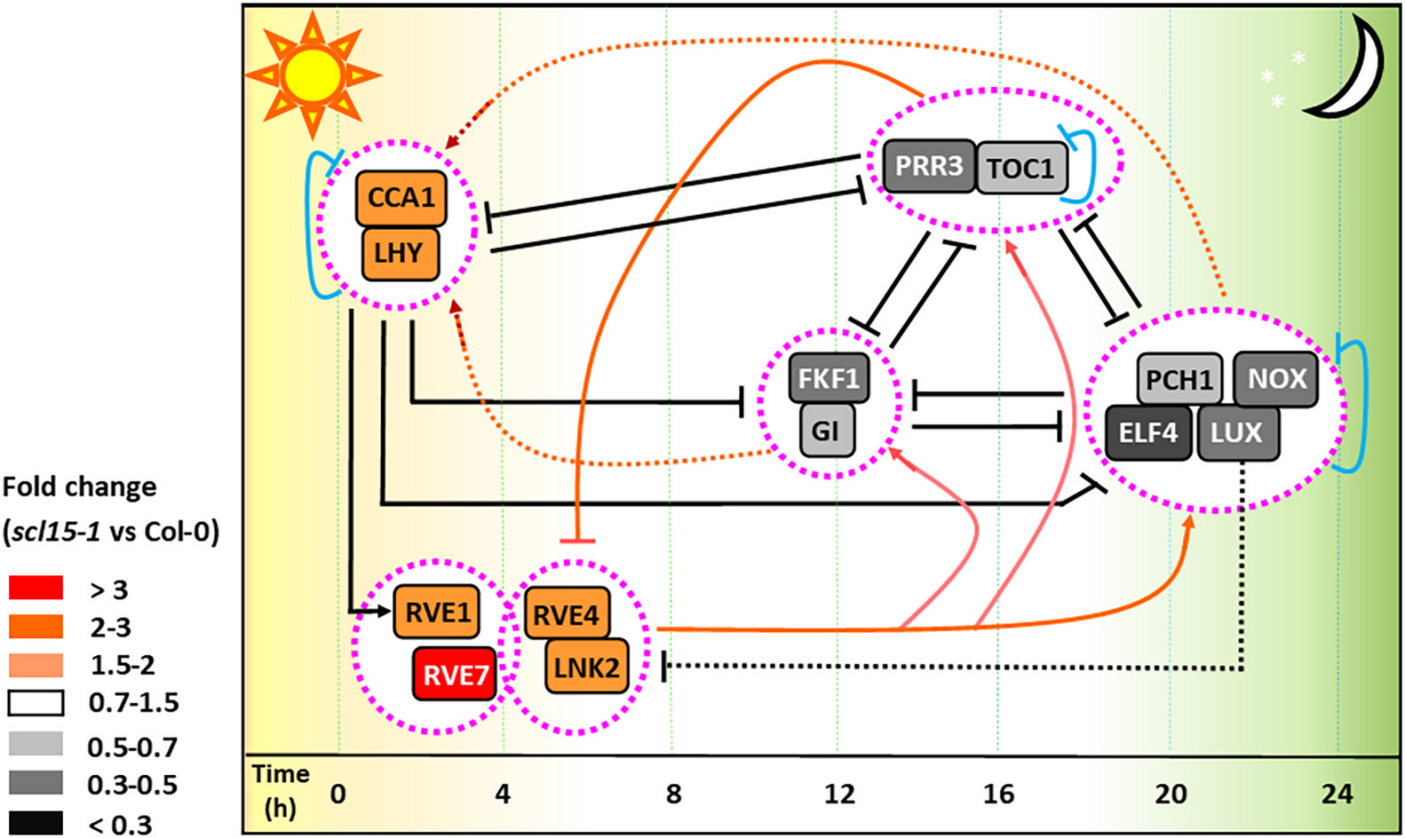
Changes in expression levels of central clock genes in mature *scl15‐1* seeds showing multiple interlocked transcriptional feedback loops at the core of the circadian oscillator. The sequential expression of each circadian component throughout the day is shown from left to right and the time of activity is expressed in hours after dawn. The yellow and dark green areas represent day and night, respectively. Bars indicate repression and arrows indicate activation of transcription. Broken lines indicate relationships that are not well defined. Outline of the core clock gene regulatory networks is derived from Hsu and Harmer (2014) and Nohales and Kay (2016). ELF4, EARLY FLOWERING 4; FKF1, FLAVIN BINDING, KELCH REPEAT, F‐BOX1; GI, GIGANTEA; LNK2, NIGHT LIGHT‐INDUCIBLE AND CLOCK‐REGULATED1; LUX, LUX ARRHYTHMO; NOX, Latin word for ‘night’; PCH1, PHOTOPERIODIC CONTROL OF HYPOCOTYL 1; PRR3, PSEUDO‐RESPONSE REGULATOR 3; RVE, REVEILLE; TOC1, TIMING OF CAB2 EXPRESSION1; Other abbreviations are in the legend to Figure 9.

A close relationship exists between the circadian clock and seed dormancy (Penfield and Hall 2009; Penfield 2017). The seed‐specific photoreceptor phytochromes (PHY) PhyD and PhyE are key entrainment factors of the internal timekeeping circadian oscillator (Figure S10) and play a role in the inhibition of seed dormancy (Penfield et al. 2010; Sánchez‐Lamas et al. 2016). Indeed, *PhyD* and *PhyE* and their activator *PIF6* (Penfield et al. 2010) were positively regulated by SCL15. Expression of most circadian oscillator components was also altered in *scl15‐1* and Napin:SCL15 during seed maturation; morning‐phased clock genes were downregulated, while evening‐phased clock genes were upregulated by SCL15 (Figures 10 and S11; Table S10). For example, genes encoding the morning‐phased, positive‐dormancy regulators CCA1, LHY (Penfield and Hall 2009), RVE1 (Jiang et al. 2016) and RVE7 (Liu, Yang, et al. 2021) were all upregulated in *scl15‐1* and downregulated in Napin:SCL15. In contrast, genes encoding key evening‐phased clock components and negative dormancy regulators, including GIGANTEA (GI), LUX ARRHYTHMO (LUX), TIMING OF CAB2 EXPRESSION1 (TOC1) and Evening Complex (EC) EARLY FLOWERING 4 (ELF4) (Penfield and Hall 2009; Zha et al. 2020), were all downregulated in *scl15‐1* and upregulated in Napin:SCL15. In addition, a subset of circadian‐regulated genes was regulated by SCL15 (Figure S11; Table S10). For example, genes encoding a group of B‐BOX (BBX) domain proteins (e.g., BBX21 and BBX22), which play a positive role in germination and photomorphogenesis (Chang et al. 2011), were positively regulated by SCL15. Conversely, genes encoding BBX proteins that function as negative regulators of germination and inhibition of hypocotyl elongation during photomorphogenesis, such as BBX19 and BBX25 (Bai et al. 2019), as well as two negative regulators of dormancy release, bZIP63 (Mair et al. 2015) and GATA TRANSCRIPTION FACTOR 21 (GATA21) (Richter et al. 2013), were repressed by SCL15 during seed maturation. Interestingly, genes encoding two morning‐phased C‐Repeat Binding Factors (CBFs) were repressed by SCL15, whereas genes encoding two evening‐expressed RNA‐binding proteins, Glycine Rich Protein 7 (GRP7) and GRP8, were positively regulated. CBFs are key TFs in the cold stress response (Park et al. 2015), and GRP7/8, also known as COLD AND CIRCADIAN‐REGULATED 2 (CCR2) and 1 (CCR1), are markers of circadian and cold responses (Schmal et al. 2013).

### 3.7 Integration of SCL15 Regulation of ABA/Auxin Signalling and Endosperm Rupture With the Circadian Clock Network

Seed dormancy induction, maintenance, and release are regulated by many factors, including light, circadian clock, temperature, phytohormones and endosperm wall biomechanics. However, little is known about how circadian rhythms interact with auxin and ABA signalling pathways for dormancy control. A subset of SCL15‐regulated genes involved in ABA and auxin signalling, as well as CW remodelling, was identified as rhythmically expressed (Figures S12–S16). The expression of many ABA‐related genes is controlled by the morning‐phased key clock regulators CCA1 and LHY and follows rhythmic oscillations (Nagel et al. 2015; Atamian and Harmer 2016; Lee et al. 2016; Adams et al. 2018). Genes encoding ABA pathway components (PYL13, NCED2, CYP707A2 and MYB96), downstream ABA signalling factors (ABI5, DREB2C and RBG7) and the auxin pathway component IAA17 were all regulated by SCL15 and are known targets of CCA1 and LHY (Nagel et al. 2015; Adams et al. 2018). Additionally, a number of SCL15‐regulated genes encoding proteins involved in CW biosynthesis, regulation and modification, including SND1, MYB46, XYL1 and XTH22, were identified to be circadian‐regulated, with *XYL1* being a direct target of CCA1 (Nagel et al. 2015).

**FIGURE 11:**
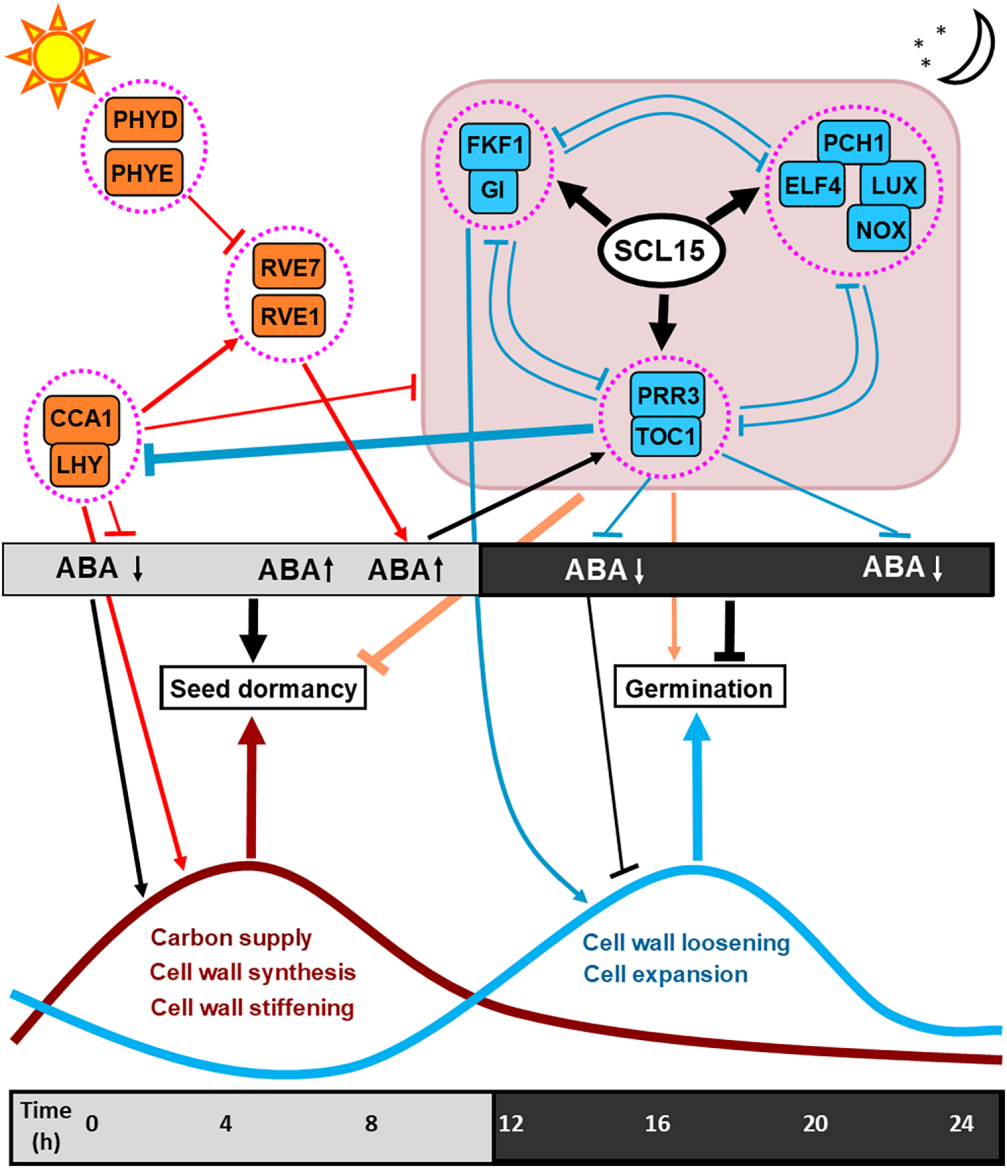
Model for dormancy control by SCL15 and its regulated factors associated with circadian clock, hormone ABA and CW biomechanics. Light signal perception and transduction are achieved by photoreceptors, such as PHYD. Circadian rhythms are translated into rhythmic oscillations of ABA accumulation peaking in the afternoon. ABA is produced primarily in the endosperm, where wall stiffening is promoted. CW synthesis requires carbon and energy and occurs mainly in the light. Starch mobilization occurs throughout the night and is almost exhausted at dawn. Seed dormancy release is regulated by SCL15 through the regulation of evening‐phased core clock genes, such as *TOC1*, *GI* and *ELF4* and hormones ABA and auxin, that, in turn, stimulate CW structural changes. Morning‐phased core clock genes, such as *CCA1*, *LHY* and *RVE1*, are repressed indirectly by SCL15. Arrows indicate transcriptional activation and bars indicate transcriptional repression.

Consistent with the dormancy phenotype of *scl15‐1*, the expression of negative dormancy regulators was positively regulated by SCL15 during seed maturation. Genes encoding circadian‐mediated, positive‐dormancy regulators that are expressed at dawn or in the early morning, such as CCA1 (Penfield and Hall 2009; Jiang et al. 2016; Liu, Yang, et al. 2021), were negatively regulated by SCL15, while those encoding evening‐phased, major clock components that form a feedback loop with CCA1 and serve as negative dormancy regulators, such as TOC1 (Penfield and Hall 2009; Legnaioli et al. 2009; Zha et al. 2020), were positively regulated by SCL15. Evening‐phased *TOC1* is induced by ABA, and this response is gated by the circadian clock via a negative feedback loop in which TOC1 represses the circadian expression of the gene encoding Mg‐chelatase H subunit (CHLH) or putative ABA receptor (ABAR) (Legnaioli et al. 2009). These rhythms are ultimately translated into rhythmic oscillations of ABA accumulation, which elevate in the early afternoon and peak in the evening (Adams et al. 2018) (Figure 11). Our results suggest that SCL15 negatively regulates primary dormancy in part through a positive effect on ABA‐regulated, evening‐phased clock genes, such as *TOC1* (Figure 11). Additionally, a significant fraction of genes encoding circadian‐regulated TFs that are involved in the regulation of dormancy and/or germination was also regulated by SCL15. For example, consistent with the germination phenotype of *scl15‐1*, genes encoding positive regulators for germination, BBX21 and BBX22 (Chang et al. 2011), were positively regulated by SCL15, while those encoding negative germination regulators, BBX19 and BBX25 (Richter et al. 2013; Mair et al. 2015; Bai et al. 2019), were negatively regulated.

In addition to ABA, auxin biosynthesis and signalling are also gated by the circadian clock, as shown by both transcriptional and growth responses to exogenous auxin (Rawat et al. 2009; Atamian and Harmer 2016; Adams et al. 2018). In this work, several genes (e.g., *TAA1* and *UGT84B1*) critical for auxin biosynthesis and signalling were expressed in the endosperm of maturing seeds and regulated by SCL15. Auxin‐induced cell expansion requires dynamic regulation of CW biosynthesis and modifications of wall components. CW biogenesis is a core event downstream of auxin regulation and is regulated by the circadian oscillator (Verbančič et al. 2018). Consistent with the theory that auxin‐induced acid growth occurs mainly at night (Verbančič et al. 2018), expression of the gene encoding the major auxin biosynthetic enzyme TAA1 is promoted by SCL15 and has high levels of expression from midday and peaks at night. Accordingly, genes encoding the master regulator SND1 required for wall thickening, the major endosperm rupture modulator XYL1, and several other positive regulators for CW stiffening, such as XTH22, which were all negatively regulated by SCL15, are expressed at the highest levels during the daytime and lowest levels at night, whereas those encoding wall‐degrading enzymes, such as PG45, have the highest expression in the evening or at night. These results demonstrate that primary seed dormancy is associated with circadian‐regulated changes in the expression of genes involved in the clock, ABA, auxin and CW remodelling pathways. Collectively, our results support the idea that SCL15 contributes to dormancy release probably through promoting expression of the evening‐phased clock genes and increasing local auxin levels, while inhibiting ABA and morning‐phased clock gene expression. These changes could in turn alter endosperm wall structure, ultimately leading to dormancy release (Figure 11).

## Author Contributions

M.‐J.G. and D.H. designed the research and wrote the paper. Q.C. and X.L. conducted germination assays and gene expression analysis. C.C., M.‐J.G. and F.F. conducted informatics analysis and data interpretation. B.Y. contributed to the construction of plant binary expression vectors. Z.J.C. reviewed and discussed the results and edited the manuscript. All authors contributed to the article and approved the submitted version.

## Conflicts of Interest

The authors declare no conflicts of interest.

## Supporting information


**TABLE S1:** Oligonucleotides used in this study.

**Data S1:** ppl70467‐sup‐0002‐Tables.docx. **TABLE S2:** Summary of Illumina transcriptome sequencing results for *scl15‐1*, Napin:SCL15 and Col‐0.
**TABLE S5:** GO terms significantly enriched in 178 genes that were upregulated in *scl15‐1* and downregulated in Napin:SCL15 (FDR < 0.05).
**TABLE S6:** GO terms in 314 genes that were downregulated in the mutant *scl15‐1* and upregulated in Napin:SCL15 (FDR < 0.05).
**TABLE S7:** Selected cell‐wall remodelling genes with altered expression in developing seeds of the *scl15‐1* and Napin:SCL15 as identified by RNA‐seq analysis (FDR < 0.05).
**TABLE S8:** Selected genes with altered expression in mature seeds of *scl15‐1* and Napin:SCL15 and involved in seed dormancy through ABA signalling as identified by RNA‐seq analysis (FDR < 0.05).
**TABLE S9:** Selected genes with altered expression in developing seeds of the *scl15‐1* and *Napin:SCL15* and involved in seed dormancy and germination through auxin signalling as identified by RNA‐seq analysis (FDR < 0.05).
**TABLE S10:** Selected genes functioning at the core clock network and their associated factors with altered expression in maturing seeds of the *scl15‐1* and Napin:SCL15 as identified by RNA‐seq analysis (FDR < 0.05).

**TABLE S3:** GO term association with DNA Motif D discovered from the 757 genes that were downregulated in scl15‐1 compared with wild type Col‐0.

**TABLE S4:** GO term association with DNA Motif DU discovered from the 314 genes that were downregulated in scl15‐1, but upregulated in Napin:SCL15 compared with wild type Col‐0.

**Data S2:** ppl70467‐sup‐0005‐Figures.docx. **Figure S1:** qRT‐PCR analysis of *SCL15* expression in leaves of 4‐week old seedlings (left) or siliques 14 days post‐anthesis (right) of wild type *A. thaliana* Col‐0, *scl15‐1*, Napin:SCL15 and 35S:SCL15 plants. *SCL15* RNA levels in the wild type Col‐0 were designated as one‐fold.
**Figure S2:** Principal component analysis (PCA) of global gene expression data in Arabidopsis seeds of wild‐type Col‐0 (WT), *scl15‐1* mutant and Napin:SCL15 overexpression lines. Three biological replicates for each line are indicated as rep1, rep2 and rep3.
**Figure S3:** Validation of RNAseq data with real‐time qRT‐PCR. Genes that are downregulated in *scl15‐1* and upregulated in the Napin:SCL15 lines are selected for expression analysis. Gene IDs: RAB18, AT5G66400; EM1, AT3G51810; EM6, AT2G40170; LEA18, AT2G35300; M10, AT2G41280; LEA4‐5, AT5G06760; XERO1, AT3G50980; IAA34, AT1G15050; ABI5, AT2G36270; HAI3, AT2G29380; PIF6, AT3G62090; HSP26.5, AT1G52560; HSP70‐5, AT1G16030; HSP17.4A, AT3G46230; HSP70‐8, AT2G32120; HSP17.6C, AT1G53540; PIMT2, AT5G50240; HVA22B, AT5G62490; MSH3, AT4G25540; MSD2, AT3G56350; COR27, AT5G42900; DREB2E, AT2G38340; DREB2G, AT5G18450; MYB90, AT1G66390; MYB21, AT3G27810; ABR1, AT5G64750; DOG1, AT5G45830; ATECP31, AT3G22500; ATRAB28, AT1G03120; OLEO4, AT3G27660; OLEO1, AT4G25140.
**Figure S4:** The pattern of expression across different physiological states for selected CW‐related genes in Arabidopsis seeds. (A) Expression of wall genes that are upregulated in *scl15‐1* and downregulated in Napin:SCL15. Note that these genes showed higher levels of expression in the imbibed, after‐ripened (AR) state without germination (DL) compared with the imbibed and germinated AR state (LIG) or any of the dormant states, indicating a role in dormancy maintenance. (B) Expression patterns of wall‐related genes that are downregulated in *scl15‐1* and upregulated in Napin:SCL15. Note that these genes displayed higher levels of expression during germination induction in imbibed dormant states compared with imbibed AR states, indicating a positive role in the initiation of germination and dormancy release. For the treatment of dormant imbibed state, PD 24 h, primary dormant seeds were imbibed for 24 h (will not complete germination). PD 48 h, primary dormant seeds were imbibed for 24 h (will not complete germination). PD 30 days, primary dormant seeds were imbibed for 30 days in the dark, a dormant imbibed state. For the treatment of imbibed after‐ripened state, DL, dry seeds were after‐ripened for 120 days and then imbibed for 24 h (will germinate if placed in the light). LIG, dry seeds were after‐ripened for 120 days and then imbibed for 20 h in the dark, and then place for 4 h under red light to terminate dormancy and induce germination (will complete germination) (Cadman et al., 2006). Data are derived from the *Arabidopsis* eFP Browser (http://bar.utoronto.ca/efp/cgi‐bin/efpWeb.cgi).
**Figure S5:** Expression of endo‐β‐mannanases (MANs) in developing seeds and during germination showing the unique expression patterns for MAN1/AtMAN1. Data are derived from the Arabidopsis eFP Browser. Noted that compared with the other seven members of the *A. thaliana* MAN protein family, *MAN1/AtMAN5‐1* was the most highly expressed MAN gene in the endosperm and its expression was highest in the ME during stratification and initiation of seed germination, suggesting that MAN1 plays a role in the modulation of seed dormancy maintenance and dormancy release.
**Figure S6:** Seed germination of mutant *scl15‐1* and *SCL15*‐overexpression lines Napin:SCL15 and 35S:SCL15 and their response to ABA biosynthesis inhibitor NDGA. (A) to (C) Germination of wild type Col‐0, *scl15‐1*, Napin:SCL15 and 35S:SCL15 in response to NDGA treatment. Stratified seeds were sown onto ½ MS agar medium supplemented with 0 (control) (A), 25 (B) or 50 μm (C) NDGA. (D) Enhancement of germination speed of *scl15‐1* in response to NDGA after imbibition for 48 h. Percentages of seed germination are means (±SD) from four biological replicates. Asterisks in (D) indicate a statistically significant difference between control and 25 or 50 μm NDGA treatment (*p < 0.05; Student's *t* test).
**Figure S7:** Expression profiles of genes encoding SEED DORMANCY FOUR‐LIKE (SFL) family proteins in developing seeds and during germination. Data are derived from the Arabidopsis eFP Browser (http://bar.utoronto.ca/efp/cgi‐bin/efpWeb.cgi).
**Figure S8:** Expression patterns of *LAFL* genes (*LEC1*, *LEC2*, *ABI3* and *FUS3*) and *L1L* in developing seeds showing specific transcript accumulation of *LEC1* in the endosperm of mature seed. Data are derived from the *Arabidopsis* eFP Browser (http://bar.utoronto.ca/efp/cgi‐bin/efpWeb.cgi).
**Figure S9:** Germination of *scl15‐1*, Napin:SCL15 and 35S:SCL15 seeds in response to 2,4‐D and the auxin biosynthetic inhibitor TIBA. (A) Germination of wild type Col‐0, *scl15‐1*, Napin:SCL15 and 35S:SCL15 in response to 2,4‐D treatment after imbibition for 24 h, 48 h or 72 h. Stratified seeds were sown onto ½ MS agar medium supplemented with 0, 0.2 or 2 nM 2,4‐D. Percentages of seed germination are means (±SD) of four biological replicates. Asterisks indicates a statistically significant difference between the control and 2,4‐D treatment (*p* < 0.05; Student's *t* test). (B) Inhibition of germination of *scl15‐1* in response to TIBA. Seeds were sown on ½ MS medium supplemented with 0 or 50 μM TIBA. Percentages of seed germination are means (±SD) from four biological replicates. Statistically significant differences between the control and 50 μM TIBA treatment were determined using a Student's *t* test (*p* < 0.01).
**Figure S10:** Expression of phytochrome (PHY) *PhyA, PhyB*, *PhyD* and *PhyE* in developing seeds, embryos and during germination. Data are derived from the Arabidopsis eFP Browser (http://bar.utoronto.ca/efp/cgi‐bin/efpWeb.cgi).
**Figure S11:** Changes in expression of circadian‐regulated genes in maturing *scl15‐1* seeds. BBX, B‐BOX DOMAIN PROTEIN; bZIP63; BASIC LEUCINE ZIPPER63; COR27, COLD REGULATED GENE 27; ERD7, EARLY‐RESPONSIVE TO DEHYDRATION 7; GNC, GATA, NITRATE‐INDUCIBLE, CARBON METABOLISM INVOLVED; GRP, GLYCINE‐RICH RNA‐BINDING PROTEIN; JMJD5, JUMONJI DOMAIN CONTAINING 5; PHYD, PHYTOCHROME D; PHYE, PHYTOCHROME D; PIF, PHYTOCHROME INTERACTING FACTOR 3‐LIKE; SOM, SOMNUS; Other abbreviations are in Figure 10.
**Figure S12:** Rhythmic expression of *SCL15* and selected genes that are regulated by SCL15 and involved in ABA and auxin signalling or cell wall remodelling. Changes in expression of circadian‐regulated genes in maturing *scl15‐1* seeds are derived from the *Arabidopsis* eFP Browser (http://bar.utoronto.ca/efp/cgi‐bin/efpWeb.cgi).
**Figure S13:** Rhythmic expression of genes that are regulated by SCL15 and involved in ABA biosynthesis, metabolism and signalling. Data are derived from the *Arabidopsis* eFP Browser (http://bar.utoronto.ca/efp/cgi‐bin/efpWeb.cgi).
**Figure S14:** Rhythmic expression of genes that are regulated by SCL15 and responsive to ABA. Data are derived from the *Arabidopsis* eFP Browser (http://bar.utoronto.ca/efp/cgi‐bin/efpWeb.cgi).
**Figure S15:** Rhythmic expression of genes that are regulated by SCL15 and involved in auxin metabolism and signalling. Data are derived from the *Arabidopsis* eFP Browser (http://bar.utoronto.ca/efp/cgi‐bin/efpWeb.cgi).
**Figure S16:** Rhythmic expression of genes that are regulated by SCL15 and involved in the regulation, biosynthesis and modification of cell wall.

## Data Availability

The data that support the findings of this study are available from the corresponding authors upon reasonable request. The silique RNA‐seq dataset can be accessed in the NCBI GEO database as accession GSE160707.
