# Supplementary material for: SCL15 Regulates the Release of Seed Dormancy in Arabidopsis thaliana by Integrating the Circadian Clock, Hormonal Signals and Cell Wall Remodelling
Source: Physiol Plant. 2025 Sep 8;177(5):e70467. doi: 10.1111/ppl.70467 (PMC12415678; doi:10.1111/ppl.70467)
Supplement: Supplementary file 2 — Data S1: ppl70467‐sup‐0002‐Tables.docx. TABLE S2: Summary of Illumina transcriptome sequencing results for scl15‐1, Napin:SCL15 and Col‐0. TABLE S5: GO terms significantly enriched in 178 genes that were upregulated in scl15‐1 and downregulated in Napin:SCL15 (FDR < 0.05). TABLE S6: GO terms in 314 genes that were downregulated in the mutant scl15‐1 and upregulated in Napin:SCL15 (FDR < 0.05). TABLE S7: Selected cell‐wall remodelling genes with altered expression in developing seeds of the scl15‐1 and Napin:SCL15 as identified by RNA‐seq analysis (FDR < 0.05). TABLE S8: Selected genes with altered expression in mature seeds of scl15‐1 and Napin:SCL15 and involved in seed dormancy through ABA signalling as identified by RNA‐seq analysis (FDR < 0.05). TABLE S9: Selected genes with altered expression in developing seeds of the scl15‐1 and Napin:SCL15 and involved in seed dormancy and germination through auxin signalling as identified by RNA‐seq analysis (FDR < 0.05). TABLE S10: Selected genes functioning at the core clock network and their associated factors with altered expression in maturing seeds of the scl15‐1 and Napin:SCL15 as identified by RNA‐seq analysis (FDR < 0.05). [file PPL-177-e70467-s004.docx]

**TABLE S2.** Summary of Illumina transcriptome sequencing results for *scl15-1*, Napin:SCL15 and Col-0.

| Reads category | *scl15-1* | Napin:SCL15 | Col-0 |
| --- | --- | --- | --- |
| Total clean reads | 72893987 | 82987747 | 67297139 |
| Total mapped reads (% of clean reads) | 68868421 (95%) | 79633760 (96%) | 62371287 (93%) |
| Multiple mapped reads (% of mapped reads) | 7186991 (14%) | 8453485 (14%) | 9313231 (19%) |
| Uniquely mapped reads (% of mapped reads) | 44257885 (86%) | 54081074 (86%) | 40117165 (81%) |

**TABLE S5.** GO terms significantly enriched in 178 genes that were upregulated in *scl15-1* and downregulated in Napin:SCL15 (FDR<0.05)

| **GO term** | **Description** | **Number in input list** | ***p*-value** |
| --- | --- | --- | --- |
| GO:0009416 | response to light stimulus | [37](http://systemsbiology.cpolar.cn/agriGOv2/termDetail.php?session=462771824.1&GO=GO:0009416) | 7.5e-23 |
| GO:0009628 | response to abiotic stimulus | [55](http://systemsbiology.cpolar.cn/agriGOv2/termDetail.php?session=462771824.1&GO=GO:0009628) | 2.5e-22 |
| GO:0015979 | photosynthesis | [4](http://systemsbiology.cpolar.cn/agriGOv2/termDetail.php?session=462771824.1&GO=GO:0015979) | 5.1e-21 |
| GO:0009725 | response to hormone | [39](http://systemsbiology.cpolar.cn/agriGOv2/termDetail.php?session=462771824.1&GO=GO:0009725) | 1.3e-13 |
| GO:2001141 | regulation of transcription, DNA-templated | 47 | 4.6e-13 |
| GO:1901700 | response to oxygen-containing compound | [36](http://systemsbiology.cpolar.cn/agriGOv2/termDetail.php?session=462771824.1&GO=GO:1901700) | 3.5e-12 |
| GO:0009768 | photosynthesis, light harvesting in photosystem I | 8 | 9.4e-12 |
| GO:0009755 | hormone-mediated signaling pathway | [33](http://systemsbiology.cpolar.cn/agriGOv2/termDetail.php?session=462771824.1&GO=GO:0009755) | 4.7e-14 |
| GO:0009637 | response to blue light | [10](http://systemsbiology.cpolar.cn/agriGOv2/termDetail.php?session=462771824.1&GO=GO:0009637) | 1.8e-11 |
| GO:0010218 | response to far red light | [13](http://systemsbiology.cpolar.cn/agriGOv2/termDetail.php?session=462771824.1&GO=GO:0010218) | 1.7e-09 |
| GO:0048511 | rhythmic process | [11](http://systemsbiology.cpolar.cn/agriGOv2/termDetail.php?session=462771824.1&GO=GO:0048511) | 2.9e-09 |
| GO:0007623 | circadian rhythm | [10](http://systemsbiology.cpolar.cn/agriGOv2/termDetail.php?session=462771824.1&GO=GO:0007623) | 1.7e-08 |
| GO:0009755 | hormone-mediated signaling pathway | 21 | 2.6e-07 |
| GO:0009733 | response to auxin | [4](http://systemsbiology.cpolar.cn/agriGOv2/termDetail.php?session=462771824.1&GO=GO:0009733) | 1.5e-09 |
| GO:0009266 | response to temperature stimulus | [15](http://systemsbiology.cpolar.cn/agriGOv2/termDetail.php?session=462771824.1&GO=GO:0009266) | 2e-06 |
| GO:0009737 | response to abscisic acid | [5](http://systemsbiology.cpolar.cn/agriGOv2/termDetail.php?session=462771824.1&GO=GO:0009737) | 2.9e-06 |
| GO:0009734 | auxin-activated signaling pathway | [11](http://systemsbiology.cpolar.cn/agriGOv2/termDetail.php?session=462771824.1&GO=GO:0009734) | 3.3e-06 |
| GO:0009409 | response to cold | [2](http://systemsbiology.cpolar.cn/agriGOv2/termDetail.php?session=462771824.1&GO=GO:0009409) | 4.9e-06 |
| GO:0071365 | cellular response to auxin stimulus | [9](http://systemsbiology.cpolar.cn/agriGOv2/termDetail.php?session=462771824.1&GO=GO:0071365) | 6.1e-06 |
| GO:0009753 | response to jasmonic acid | 9 | 6.8e-06 |
| GO:0009723 | response to ethylene | [10](http://systemsbiology.cpolar.cn/agriGOv2/termDetail.php?session=462771824.1&GO=GO:0009723) | 2.2e-05 |
| GO:0010467 | gene expression | 49 | 6.4e-05 |
| GO:0009893 | positive regulation of metabolic process | 12 | 0.00012 |
| GO:0071669 | plant-type cell wall organization or biogenesis | [8](http://systemsbiology.cpolar.cn/agriGOv2/termDetail.php?session=462771824.1&GO=GO:0071669) | 0.00019 |
| GO:0071554 | cell wall organization or biogenesis | [12](http://systemsbiology.cpolar.cn/agriGOv2/termDetail.php?session=462771824.1&GO=GO:1901698) | 0.0009 |
| GO:0042546 | cell wall biogenesis | 8 | 0.003 |

**TABLE S6.** GO terms in 314 genes that were downregulated in the mutant *scl15-1* and upregulated in Napin:SCL15 (FDR<0.05)

| **GO term** | **Description** | **Number in input list** | ***p*-value** |
| --- | --- | --- | --- |
| GO:0048316 | seed development | [39](http://systemsbiology.cpolar.cn/agriGOv2/termDetail.php?session=345751978.1&GO=GO:0048316) | 5.6e-17 |
| GO:0009791 | post-embryonic development | 59 | 2.4e-16 |
| GO:0009628 | response to abiotic stimulus | [63](http://systemsbiology.cpolar.cn/agriGOv2/termDetail.php?session=345751978.1&GO=GO:0009628) | 7.5e-14 |
| GO:1901700 | response to oxygen-containing compound | [4](http://systemsbiology.cpolar.cn/agriGOv2/termDetail.php?session=345751978.1&GO=GO:1901700) | 1.1e-13 |
| GO:0009266 | response to temperature stimulus | [30](http://systemsbiology.cpolar.cn/agriGOv2/termDetail.php?session=345751978.1&GO=GO:0009266) | 2.7e-12 |
| GO:0010431 | seed maturation | [13](http://systemsbiology.cpolar.cn/agriGOv2/termDetail.php?session=345751978.1&GO=GO:0010431) | 4.9e-12 |
| GO:0009415 | response to water | 22 | 1.9e-10 |
| GO:0009408 | response to heat | [6](http://systemsbiology.cpolar.cn/agriGOv2/termDetail.php?session=345751978.1&GO=GO:0009408) | 1.9e-09 |
| GO:0009737 | response to abscisic acid | [6](http://systemsbiology.cpolar.cn/agriGOv2/termDetail.php?session=345751978.1&GO=GO:0009737) | 2.9e-09 |
| GO:0009793 | embryo development ending in seed dormancy | 23 | 7.4e-09 |
| GO:0007623 | circadian rhythm | [13](http://systemsbiology.cpolar.cn/agriGOv2/termDetail.php?session=345751978.1&GO=GO:0007623) | 8.2e-09 |
| GO:0010162 | seed dormancy process | [8](http://systemsbiology.cpolar.cn/agriGOv2/termDetail.php?session=345751978.1&GO=GO:0010162) | 2.6e-08 |
| GO:0009644 | response to high light intensity | [10](http://systemsbiology.cpolar.cn/agriGOv2/termDetail.php?session=345751978.1&GO=GO:0009644) | 3.2e-08 |
| GO:0055114 | oxidation-reduction process | 43 | 4.3e-08 |
| GO:0009642 | response to light intensity | [12](http://systemsbiology.cpolar.cn/agriGOv2/termDetail.php?session=345751978.1&GO=GO:0009642) | 1.4e-07 |
| GO:0000302 | response to reactive oxygen species | 12 | 6e-07 |
| GO:0009639 | response to red or far red light | [12](http://systemsbiology.cpolar.cn/agriGOv2/termDetail.php?session=345751978.1&GO=GO:0009639) | 1.3e-05 |
| GO:0009409 | response to cold | [15](http://systemsbiology.cpolar.cn/agriGOv2/termDetail.php?session=345751978.1&GO=GO:0009409) | 3.5e-05 |
| GO:0009845 | seed germination | [9](http://systemsbiology.cpolar.cn/agriGOv2/termDetail.php?session=345751978.1&GO=GO:0009845) | 0.0001 |

**TABLE S7.** Selected cell-wall remodeling genes with altered expression in developing seeds of the *scl15-1* and Napin:SCL15 as identified by RNA-seq analysis (FDR < 0.05).

| **Gene Name** | **Locus Identifier (AGI)** | **Fold change (*scl15-1* vs Col-0)** | **Fold Change (Napin:SCL15 vs Col-0)** | **Molecular Function** | **Transcript Localization in Developing Seed*** |
| --- | --- | --- | --- | --- | --- |
| *SND1* (*NST3*) | AT1G32770 | 2.8665 | -1.6066 | Master regulator of secondary cell-wall (SCW) formation (Jeong et al., 2018) | Expressed in all seed regions and subregions (Belmonte et al., 2013) |
| *MYB46* | AT5G12870 | 1.7537 | -2.2415 | Master regulator of SCW biosynthesis (Zhong et al., 2007; Ko et al., 2009) | Expressed in all seed regions and subregions (Belmonte et al., 2013) |
| *MYB4* | AT4G38620 | 1.6675 | -1.5282 | Positive regulation of cellulose biosynthesis (Zhou et al., 2015; Yu et al., 2020) | Expressed in all seed regions and subregions (Belmonte et al., 2013) |
| *MYB29* | AT5G07690 | 1.9762 | -1.5464 | SCW formation (Cassan-Wang et al., 2013) | Expressed in all seed regions and subregions (Belmonte et al., 2013) |
| *MYB75* (*PAP1*) | AT1G56650 | -2.1258 | 1.5718 | Negative regulation of SCW formation (Bhargava et al., 2010, 2013) | Expressed in all seed regions and subregions (Belmonte et al., 2013) |
| *MYB85* | AT4G22680 | 1.9833 | -1.5311 | Positive regulation of SCW formation (Geng et al., 2020). | Relatively higher in mgMCE (Belmonte et al., 2013) |
| *WRKY12* | AT2G44745 | 1.5354 | -1.9567 | A auto-repressed negative regulator of SCW accumulation in pith parenchyma cells (Wang et al., 2010; Rao & Dixon, 2018) | Expressed in all seed regions and subregions (Belmonte et al., 2013) |
| *OFP1* | AT5G01840 | 1.7424 | -1.9912 | Deposition of SCW components and repression of GA synthesis (Li et al., 2011; Wang et al., 2007, 2016) | Expressed in all seed regions and subregions (Belmonte et al., 2013) |
| *OFP8* | AT5G19650 | 11.1504 | -19.8838 | Deposition of SCW components and repression of GA synthesis (Li et al., 2011; Wang et al., 2007, 2016) | Expressed in all seed regions and subregions (Belmonte et al., 2013) |
| *OFP15* | A2G36050 | 2.6104 | -1.5187 | Deposition of SCW components and repression of GA synthesis (Li et al., 2011; Wang et al., 2007, 2016) | Expressed in all seed regions and subregions (Belmonte et al., 2013) |
| *ANAC032* | AT1G77450 | -1.7133 | 1.5047 | Negatively regulates anthocyanin and SCW biosynthesis (Taylor-Teeples et al., 2015; Mahmood et al., 2016). | Moderately abundant in all regions of seed including endosperm but relatively higher in mgEP (Belmonte et al., 2013) |
| ABS5/T5L1 | AT1G68810 | 2.0171 | -1.8712 | SCW formation (Cassan-Wang et al., 2013) | Expressed in endosperm but relatively higher in CZSC (Belmonte et al., 2013) |
| *AIL6* | AT5G10510 | -1.5317 | 1.8487 | Regulates pectin and SCW biosynthesis (Taylor-Teeples et al., 2015; Krizek et al., 2016). | Moderately abundant in all regions of seed but more abundant in MCE (Belmonte et al., 2013) |
| *SKS6* | AT1G41830 | -2.3789 | 1.7621 | Negatively regulates cell-wall thickening (predictive) (Zhou, 2019). | Expressed in all seed regions but relatively higher in mgMCE and mgEP (Belmonte et al., 2013) |
| *AtEPR1* | AT2G27380 | -1.6795 | 1.5486 | Endosperm cell-wall softening and GA-induced activator of germination (Dubreucq et al., 2000; Bethke et al., 2007). | Abundant in mgMCE, mgPEN and mgCZE (Belmonte et al., 2013) |
| *RABA6a* | AT1G73640 | 3.4202 | -1.5420 | Trafficking of SCW components (Lycett, 2008; Oda & Fukuda, 2014; Sinclair et al., 2018) | Expressed in all seed regions and subregions (Belmonte et al., 2013) |
| *MAP65-2* | AT4G26760 | 1.7369 | -1.5384 | Deposition of SCW components (Mao et al., 2006; Zhong & Ye, 2015) | Moderately abundant in all regions of seed including endosperm (Belmonte et al., 2013) |
| *MAP65-3* | AT5G51600 | 1.5146 | -1.5356 | Deposition of SCW components (Müller et al., 2004; Mao et al., 2006; Zhong & Ye, 2015) | Moderately abundant in all regions of seed including endosperm (Belmonte et al., 2013) |
| *MAP65-4* | AT3G60840 | 3.6035 | -7.7949 | Deposition of SCW components (Mao et al., 2006; Fache et al. 2010; Zhong & Ye, 2015) | Less abundant in all regions of seed including endosperm (Belmonte et al., 2013) |
| *XYL1/TRG1* | AT1G68560 | 2.1959 | -1.5297 | Xyloglucan maturation, cell wall integrity and seed dormancy maintenance (Sechet et al., 2016; Shigeyama et al., 2016) | Expressed in endosperm but relatively higher in lcPEN (Belmonte et al., 2013) |
| *CEL1/AtGH9B1* | AT1G70710 | 2.0249 | -2.5111 | Positively regulates cellulose biosynthesis and wall thickening (Shani et al., 2006) | Expressed in all seed regions and subregions (Belmonte et al., 2013) |
| *COBL4* (*IRX6*) | AT5G15630 | 1.5220 | -2.3728 | Positively regulates cellulose biosynthesis and C partitioning (Taylor-Teeples et al., 2015). | Expressed in all seed regions and subregions (Belmonte et al., 2013) |
| *XTH11* | AT3G48580 | -3.0238 | 1.5369 | Cell-wall remodeling and loosening (Endo et al., 2012; De Caroli et al., 2021). | Moderately abundant in all regions of seed including endosperm but relatively higher in lc stage (Belmonte et al., 2013) |
| *XTH22*/*TCH4* | AT5G57560 | 2.0488 | -1.6686 | Contributes to cell-wall stiffening (Xu et al., 1995; Antosiewicz et al., 1997; Nishitani, 1997) | Moderately abundant in all regions of seed including endosperm (Belmonte et al., 2013) |
| *XTH25* | AT5G57550 | -3.3211 | 2.6785 | Cell-wall remodeling and loosening (Xu et al., 1996; Becnel et al., 2006; Jobert et al., 2021). | Less abundant in all regions of seed including endosperm but relatively higher in mgEP (Belmonte et al., 2013) |
| *GUX2* | AT4G33330 | 1.8581 | -1.5540 | Plays a role in SCW hemicellulose xylan backbone biosynthesis (Mortimer et al., 2010). | Moderately abundant in all regions of seed including endosperm but relatively higher in PEN (Belmonte et al., 2013) |
| *PME5* | AT5G47500 | 6.2053 | -1.7991 | Positively regulates cell-wall stiffening (Rockel et al., 2008; Phyo et al., 2017) | Expressed in all seed regions and subregions (Belmonte et al., 2013) |
| *PME16* | AT2G43050 | 4.0866 | -1.5789 | Homologue of PME35. May positively regulates cell-wall stiffening (Hongo et al., 2012). | Moderately abundant in all regions of seed including endosperm but more abundant in SC (Belmonte et al., 2013) |
| *PME35* | AT3G59010 | 1.5368 | -1.5300 | Positive factor of mechanical support for primary cell wall (Hongo et al., 2012). | Expressed in all seed regions but relatively higher in CZSC (Belmonte et al., 2013) |
| *PME36* | AT3G60730 | -1.5698 | 1.8634 | Has PME activity but no effect on germination (Jobert et al., 2022) | Expressed in all seed regions but relatively higher in mgEP and mgPEN (Belmonte et al., 2013) |
| *PMEI10* | AT1G62760 | -2.4616 | 1.7096 | Negatively regulates cell-wall stiffening (Rockel et al., 2008; Phyo et al., 2017) | Expressed in all seed regions and subregions (Belmonte et al., 2013) |
| *PDCB2* | AT5G08000 | 2.2721 | -2.0049 | Callose deposition in cell wall and cell-to-cell trafficking (Simpson et al., 2009) | Less abundant in all regions of seed but relatively higher in lcMCE (Belmonte et al., 2013) |
| *EXPA1* | AT1G69530 | 2.0339 | -1.5063 | Positively regulates cell wall stiffness (Samalova et al., 2020). | Expressed in all seed regions but relatively higher in mgPEN and mgEP (Belmonte et al., 2013) |
| *EXPA10* | AT1G26770 | 3.0316 | -3.4193 | Induces cell wall creep without weakening the wall (Cosgrove, 2015, 2018, 2022) | Moderately abundant in all regions of seed including endosperm (Belmonte et al., 2013) |
| *BGAL3* | AT4G36360 | -1.5730 | 1.9020 | Seed-specific endosperm-abundant β-galactosidase modifying cell wall through interaction with XyG (Moneo-Sánchez et al., 2019) | Moderately abundant in all regions of seed but more abundant in the endosperm at mg stage (Belmonte et al., 2013) |
| *BGAL5* | AT1G45130 | 1.5468 | -1.5344 | Increases accumulation of HG and thicken cell walls (Bhargava et al., 2010; Martin et al., 2011) | Moderately abundant in all regions of seed but more abundant in SC (Belmonte et al., 2013) |
| *AtBXL1* | AT5G49360 | 1.8866 | -1.5192 | Pectic arabinan side chain modification and SCW thickening (Goujon et al., 2003) | Moderately abundant in all regions of seed but more abundant in SC and CZSC (Belmonte et al., 2013) |
| *RGP3* | AT3G08900 | 2.8221 | -1.8056 | Contributes to endosperm cell wall synthesis through arabinose metabolism (Rautengarten et al., 2011) | Moderately abundant in all regions of seed but more abundant in lcMCE and lcPEN (Belmonte et al., 2013) |
| *AtMAN1* | AT1G02310 | -2.19779 | 1.65365 | Cell wall degradation through hydrolysis of hemicellulose mannan and positive regulation of seed germination (Iglesias-Fernandez and Matilla, 2009; Iglesias-Fernández et al., 2011). | Moderately abundant in mgMCE (Belmonte et al., 2013) |
| *PG45* | AT1G02790 | -4.4626 | -2.2636 | Contributes to pectin HG degradation and cell proliferation (Yang et al., 2021). | Less abundant in all regions of seed including endosperm but relatively higher in lc stage (Belmonte et al., 2013) |

***** **CZE**, Chalazal endosperm; **CZSC**, Chalazal seed coat; **EP**, Embryo proper; **g**, Globular; **h**, Heart; **lc**, Linear cotyledon; **MCE**, Micropylar endosperm; **mg**, Mature green; **PEN**, Peripheral endosperm; **pg**, Preglobular; **SUS**, Suspensor; **SC**, Distal seed coat (Belmonte et al., 2013).

**References:**

Belmonte, M. F., Kirkbride, R. C., Stone, S. L., et al., 2013. Comprehensive developmental profiles of gene activity in regions and subregions of the Arabidopsis seed. *Proceedings of the National Academy of Sciences* 110: E435-E444.

Cassan-Wang, H., Goué, N., Saidi, M. N., Legay, S., Sivadon, P., Goffner, D. and Grima-Pettenati, J. 2013. Identification of novel transcription factors regulating secondary cell wall formation in Arabidopsis. *Frontiers in Plant Science* 4: 189

Dubreucq, B., Berger, N., Vincent, E., Boisson, M., Pelletier, G., Caboche, M. and Lepiniec, L. 2000. The Arabidopsis AtEPR1 extensin‐like gene is specifically expressed in endosperm during seed germination. *The Plant Journal* 23: 643-652.

McGee, R., Dean, G. H., Wu, D., Zhang, Y., Mansfield, S. D. and Haughn, G. W. 2021. Pectin modification in seed coat mucilage by in vivo expression of rhamnogalacturonan-I-and homogalacturonan-degrading enzymes. *Plant and Cell Physiology* 62: 1912-1926.

Yang, Y., Anderson, C. T. and Cao, J. 2021. Polygalacturonase45 cleaves pectin and links cell proliferation and morphogenesis to leaf curvature in Arabidopsis thaliana. *The Plant Journal* 106: 1493-1508.

Zhou, Y., Liu, X., Engstrom, E. M., et al. 2015. Control of plant stem cell function by conserved interacting transcriptional regulators. *Nature* 517: 377-380.

**TABLE S8.** Selected genes with altered expression in mature seeds of *scl15-1* and Napin:SCL15 and involved in seed dormancy through ABA signalling as identified by RNA-seq analysis (FDR < 0.05).

| **Gene Annotation** | **Gene Name** | **Locus Identifier (AGI)** | **Fold change (*scl15-1* vs Col-0)** | **Fold Change (Napin:SCL15 vs Col-0)** | **Molecular Function** |
| --- | --- | --- | --- | --- | --- |
| *Nine-cis-epoxycarotenoid dioxygenase 2* | *NCED2* | AT4G18350 | -26.2970 | 1.5326 | ABA biosynthesis |
| *NINE-cis-EPOXYCAROTENOID DIOXYGENASE 4* | *NCED4* | AT4G19170 | 2.7876 | -2.5054 | ABA biosynthesis |
| *Abscisic acid 8'-hydroxylase 1* | *CYP707A1* | AT4G19230 | -1.5223 | 1.6899 | ABA catabolism |
| *Abscisic acid 8'-hydroxylase 2* | *CYP707A2* | AT2G29090 | -1.7790 | 1.5237 | ABA catabolism |
| *PYR1-like12* | *PYL12* | AT5G45870 | -3.8453 | 3.3381 | ABA receptor |
| *PYR1-like13* | *PYL13* | AT4G18620 | -3.1862 | 2.9195 | ABA receptor |
| *G-PROTEIN COUPLED RECEPTOR 2* | *GCR2* | AT1G52920 | -5.8991 | 3.2469 | G-protein signaling |
| *GCR2-like1* | *GCL1* | AT5G65280 | -2.6277 | 1.5873 | G-protein signaling |
| *GCR2-like2* | *GCL2* | AT2G20770 | -2.1343 | 2.0311 | G-protein signaling |
| *ABA-hypersensitive germination 1* | *AHG1* | AT5G51760 | -3.9762 | 2.3346 | ABA signaling |
| *highly ABA-induced PP2C gene 3* | *HAI3* | AT2G29380 | -4.3334 | 2.8894 | ABA signaling |
| *Protein phosphatase 2C28* | *PP2C28* | AT2G34740 | -54.9191 | 2.4493 | ABA signaling |
| *Protein phosphatase 2C39* | *PP2C39* | AT3G15260 | -1.6534 | 1.8259 | ABA signaling |
| *Protein phosphatase 2C52* | *PP2C52* | AT4G03415 | 1.6105 | -1.8913 | G-protein signaling |
| *Protein phosphatase 2C79* | *PP2C79* | AT5G66080 | -2.2878 | 3.1182 | ABA signaling |
| *SEED DORMANCY FOUR-LIKE 1* | *SFL1* | AT1G27461 | -2.6744 | 1.9759 | Seed dormancy |
| *SEED DORMANCY FOUR-LIKE 3* | *SFL3* | AT5G63350 | -2.5520 | 2.5558 | Seed dormancy |
| *SEED DORMANCY FOUR-LIKE 4* | *SFL4* | AT5G50360 | -1.6645 | 1.9786 | Seed dormancy |
| *Delay of germination 1* | *DOG1* | AT5G45830 | -1.6403 | 1.9977 | Seed dormancy |
| *REDUCED DORMANCY 5* | *RDO5* | AT4G11040 | -1.5187 | 2.2474 | Seed dormancy |
| *DOG1-LIKE3* | *DOGL3* | AT4G18690 | -4.4675 | 2.8583 | Seed dormancy |
| *ETHYLENE RESPONSE FACTOR12* | *ERF12* | AT1G28360 | -1.5412 | 1.7876 | ABA signaling |
| *enhancer of aba co-receptor 1* | *EAR1* | AT5G22090 | -2.2367 | 1.5161 | ABA signaling |
| *myb domain protein 96* | *MYB96* | AT5G62470 | 2.5442 | -2.5476 | ABA signaling |
| *NIN-like protein 8* | *NLP8* | AT2G43500 | -1.5472 | 1.5378 | Nitrate signaling |
| *plant U-box 18* | *PUB18* | AT1G10560 | 1.9874 | -2.1880 | ABA signaling |
| *plant U-box 23* | *PUB23* | AT2G35930 | 3.1784 | -3.6638 | ABA signaling |
| *SNF1-related protein kinase 2.5* | *SnRK2.5* | AT5G63650 | 1.5446 | -1.5345 | ABA signaling |
| *SNF1-related protein kinase 2.8* | *SnRK2.8* | AT1G78290 | 2.4154 | -1.7516 | ABA signaling |
| *RELATED TO ABI3/VP1* | *RAV1* | AT1G13260 | 1.5520 | -1.5087 | ABA & G-protein signaling |
| *RAB GTPASE HOMOLOG A6A* | *RABA6A* | AT1G73640 | 3.4202 | 1.5420 | ABA signaling |
| *Mg-*chelatase*H*subunit | *ABAR/CHLH/GUN5* | AT5G13630 | 1.6045 | -1.8665 | ABA signaling |
| *CHLI subunit of magnesium chelatase* | *CHLI* | AT4G18480 | 1.5416 | -1-5409 | ABA signaling |
| *Dormancy/auxin associated family protein* | *AT1G56220* | AT1G56220 | 1.5644 | -1.5447 | Seed dormancy |
| *LEAFY COTYLEDON 1* | *LEC1* | AT1G21970 | 3.3730 | -1.5287 | Seed development |
| *LEC1-LIKE* | *L1L* | AT5G47670 | 1.7480 | -1.5788 | Seed development |
| *EARLY LIGHT-INDUCED PROTEIN1* | *ELIP1* | AT3G22840 | -5.1114 | 1.6347 | ABA signaling |
| *EARLY LIGHT-INDUCED PROTEIN2* | *ELIP2* | AT4G14690 | 1.7416 | -1.6556 | ABA signaling |
| *RARE-COLD-INDUCIBLE 2B* | *RCI2B* | AT3G05890 | 2.3951 | -5.3507 | Cold signaling |
| *C-repeat/DRE binding factor 1* | *CBF1/DREB1B* | AT4G25490 | 4.5377 | -16.2938 | Cold signaling |
| *C-repeat/DRE binding factor 2* | *CBF2/DREB1C* | AT4G25470 | 5.2077 | -20.5260 | Cold signaling |
| *ETHYLENE RESPONSE FACTOR7* | *ERF7* | At3g20310 | -1.8246 | 2.4785 | Ethylene signaling |
| *ETHYLENE RESPONSE FACTOR12* | *ERF12* | AT1G28360 | -1.5412 | 1.7876 | Ethylene signaling |

**TABLE S9.** Selected genes with altered expression in developing seeds of the *scl15-1* and *Napin:SCL15* and involved in seed dormancy and germination through auxin signaling as identified by RNA-seq analysis (FDR < 0.05)

| **Gene Name** | **Locus Identifier (AGI)** | **Fold change (*scl15-1* vs Col-0)** | **Fold Change (Napin:SCL15 vs Col-0)** | **Molecular Function** | **Reference** |
| --- | --- | --- | --- | --- | --- |
| *TAA1* | AT1G70560 | -1.5153 | 1.8673 | Auxin biosynthesis for the conversion of L-Trp to indole-3-pyruvic acid (IPyA) | Stepanova et al., 2008; |
| *CYP79B3* | AT2G22330 | 2.0115 | -4.3519 | Auxin biosynthesis for the conversion of L-Trp to indole acetaldoxime (IAOx) | [Su et al., 20](https://www.ncbi.nlm.nih.gov/pmc/articles/PMC8282758/#R50)11 |
| *CYP71A13* | AT2G30770 | 2.7884 | -1.1800 | Auxin biosynthesis for the conversion of IAOx to indole-3-acetonitrile (IAN) | [Nafisi et al., 200](https://www.ncbi.nlm.nih.gov/pmc/articles/PMC8282758/#R50)7 |
| *NIT3* | AT3G44320 | -2.1423 | -1.5335 | Auxin biosynthesis for the conversion of indole-3-acetonitrile (IAN) to indole-3-acetic acid (IAA) | **Kutz et al., 2002** |
| *IAMH1* | AT4G37550 | 1.6559 | -1.5084 | Auxin biosynthesis for the conversion of indole-3-acetamide (IAM) to IAA | Gao et al., 2020 |
| *AMI1* | AT1G08980 | 1.5467 | -1.580 | Auxin biosynthesis for the conversion of indole-3-acetamide (IAM) to IAA | Sánchez-Parra et al., 2014 |
| *UGT84B1* | AT2G23260 | 4.0991 | -1.7696 | Auxin catabolism for the formation of IAA-glucose (IAA-glc) and 2-oxoindole-3-acetic acid (oxIAA-glc) | Aoi et al., 2020 |
| *GNC/GATA21* | AT5G56860 | 1.8939 | -2.1805 | Circadian-regulated, involved in auxin signaling and positive for dormancy | Richter et al., 2013 |
| *SAUR12* | AT2G21220 | 7.1931 | -2.1467 | Clade I Small Auxin-Upregulated RNA (SAUR) induced by IAA but repressed by abscisic acid (ABA) | Hagen and Guilfoyle, 2002; van Mourik et al., 2017 |
| *SAUR16* | AT4G38860 | 7.0704 | -1.5784 | Clade I SAUR induced by IAA but repressed by abscisic acid (ABA) and involved in light response | Hagen and Guilfoyle, 2002; Dong et al., 2019; |
| *SAUR32* | AT2G46690 | 2.4512 | -2.4797 | Positive regulator of seed dormancy. Clade III SAUR induced by ABA but repressed by IAA | Hagen and Guilfoyle, 2002; van Mourik et al., 2017; He et al., 2021 |
| *SAUR50* | AT4G34760 | 2.1393 | -1.5400 | Clade I SAUR induced by IAA but repressed by abscisic acid (ABA) | Hagen and Guilfoyle, 2002; Dong et al., 2019; |
| *ARR6* | AT5G62920 | 1.5492 | -2.0492 | Type-A Responsive Regulator negatively regulating cytokinin signaling and involved in cell wall modification | Bacete et al., 2020 |
| *IAA2* | AT3G23030 | 1.6351 | -1.5466 | Canonical AUX/IAA protein involved in TIR1-dependent auxin pathway | Liscum and Reed, 2002; Peer, 2013; Luo et al., 2018 |
| *IAA7* | AT3G23050 | 2.3689 | -1.2080 | Canonical AUX/IAA protein involved in TIR1-dependent auxin pathway | Liscum and Reed, 2002; Cao et al., 2019 |
| *IAA14* | AT4G14550 | 3.6330 | -1.5796 | Canonical AUX/IAA protein involved in TIR1-dependent auxin pathway | Liscum and Reed, 2002; Peer, 2013; Luo et al., 2018 |
| *IAA16* | AT3G04730 | 1.5409 | -1.8220 | Canonical AUX/IAA protein involved in TIR1-dependent auxin pathway | Liscum and Reed, 2002; Cao et al., 2019 |
| *IAA17* | AT1G04250 | 2.6384 | -1.4521 | Canonical AUX/IAA protein involved in TIR1-dependent auxin pathway | Liscum and Reed, 2002; Peer, 2013; Luo et al., 2018 |
| *IAA32* | AT2G01200 | -1.5746 | 1.6104 | Noncanonical AUX/IAA protein involved in TMK1-dependent auxin pathway | Liscum and Reed, 2002; Cao et al., 2019 |
| *IAA34* | AT1G15050 | -5.6518 | 2.8669 | Noncanonical AUX/IAA protein involved in TMK1-dependent auxin pathway | Liscum and Reed, 2002; Cao et al., 2019 |

**References**

Bacete, L., Mélida, H., López, G., et al. 2020. Arabidopsis response regulator 6 (ARR6) modulates plant cell-wall composition and disease resistance. *Molecular Plant-Microbe Interactions* 33: 767-780.

Dong, J., Sun, N., Yang, J., et al. 2019. The transcription factors TCP4 and PIF3 antagonistically regulate organ-specific light induction of SAUR genes to modulate cotyledon opening during de-etiolation in Arabidopsis. *The Plant Cell* 31: 1155-1170.

Gao, Y., Dai, X., Aoi, Y., et al. 2020. Two homologous INDOLE-3-ACETAMIDE (IAM) HYDROLASE genes are required for the auxin effects of IAM in Arabidopsis. *Journal of Genetics and Genomics* 47: 157-165.

Hagen, G. and Guilfoyle, T. 2002. Auxin-responsive gene expression: genes, promoters and regulatory factors. *Plant Molecular Biology* 49: 373-385.

He, Y., Liu, Y., Li, M., et al. 2021. The Arabidopsis SMALL AUXIN UP RNA32 protein regulates ABA-mediated responses to drought stress. *Frontiers in Plant Science* 12: 625493.

Kutz, A., Müller, A., Hennig, P., Kaiser, W. M., Piotrowski, M. and Weiler, E. W. 2002. A role for nitrilase 3 in the regulation of root morphology in sulphur‐starving Arabidopsis thaliana. *The Plant Journal* 30: 95-106.

Liscum, E., and Reed, J. 2002. Genetics of Aux/IAA and ARF action in plant growth and development. *Plant Molecular Biology* 49: 387-400.

Luo, J., Zhou, J.-J. and Zhang, J.-Z. 2018. Aux/IAA gene family in plants: molecular structure, regulation, and function. *International Journal of Molecular Sciences* 19: 259.

Nafisi, M., Goregaoker, S., Botanga, C. J., Glawischnig, E., Olsen, C. E., Halkier, B. A. and Glazebrook, J. 2007. Arabidopsis cytochrome P450 monooxygenase 71A13 catalyzes the conversion of indole-3-acetaldoxime in camalexin synthesis. *The Plant Cell* 19: 2039-2052.

Peer, W. A., Cheng, Y. and Murphy, A. S. 2013. Evidence of oxidative attenuation of auxin signalling. *Journal of Experimental Botany* 64: 2629-2639.

Sánchez-Parra, B., Frerigmann, H., Pérez Alonso, M.-M., Carrasco Loba, V., Jost, R., Hentrich, M. and Pollmann, S. (2014) Characterization of four bifunctional plant IAM/PAM-amidohydrolases capable of contributing to auxin biosynthesis. *Plants* 3: 324-347.

Su, T., Xu, J., Li, Y., et al. 2011. Glutathione-indole-3-acetonitrile is required for camalexin biosynthesis in Arabidopsis thaliana. *The Plant Cell* 23: 364-380.

van Mourik, H., van Dijk, A. D., Stortenbeker, N., Angenent, G. C. and Bemer, M. 2017. Divergent regulation of Arabidopsis SAUR genes: a focus on the SAUR10-clade. *BMC Plant Biology* 17: 1-14.

**TABLE S10.** Selected genes functioning at the core clock network and their associated factors with altered expression in maturing seeds of the *scl15-1* and Napin:SCL15 as identified by RNA-seq analysis (FDR < 0.05).

| **Gene Name** | **Locus Identifier (AGI)** | **Fold change (*scl15-1* vs Col-0)** | **Fold Change (Napin:SCL15 vs Col-0)** | **Time of Activity** | **Molecular Function** |
| --- | --- | --- | --- | --- | --- |
| *CCA1* | AT2G46830 | 1.7632 | -2.3496 | Dawn | Positively regulates the period length of circadian rhythm. Primarily represses genes with evening element (EE). Positively regulates seed dormancy (Penfield & Hall [2009](https://onlinelibrary.wiley.com/doi/full/10.1111/jipb.13001?casa_token=ZngmsDmbCG0AAAAA%3AaPTcMJa3o90GLdRQE_kkQB5coXLPHauichG1u_A5jiqDjWxCOiw4yNyr-__9XVepOk3Rr6sy1zN1lw#jipb13001-bib-0101); Nohales & Kay, 2016) |
| *LHY* | AT1G01060 | 1.9748 | -4.8840 | Dawn | Positively regulates the period length of circadian rhythm. Partially redundant with CCA1 to regulate other clock genes and positively regulates seed dormancy (Penfield & Hall [2009](https://onlinelibrary.wiley.com/doi/full/10.1111/jipb.13001?casa_token=ZngmsDmbCG0AAAAA%3AaPTcMJa3o90GLdRQE_kkQB5coXLPHauichG1u_A5jiqDjWxCOiw4yNyr-__9XVepOk3Rr6sy1zN1lw#jipb13001-bib-0101); Nohales & Kay, 2016) |
| *TOC1* (*PRR1*) | AT5G61380 | -1.9578 | 1.5843 | Evening | Forming transcriptional repression feedback loops with other circadian clock genes (Huang et al., 2012; Gendron et al., 2012; Nohales and Kay, 2016) |
| *PRR3* | AT5G60100 | -2.7964 | 1.5095 | Evening | Modulates TOC1 stability in the vasculature (Para et al., 2007) |
| *GI* | AT1G22770 | -1.8888 | 1.5024 | Evening | Plays a critical roles in circadian timekeeping, temperature compensation and multiple  developmental and metabolic processes (Gould et al., 2006; Dalchau et al., 2011; Mishra and Panigrahi, 2015) |
| *RVE1* | AT5G17300 | 1.8499 | -1.9669 | Dawn | Promotes seed dormancy through interaction with clock genes and GA signaling (Jiang et al., 2016; Yang et al., 2020) |
| *RVE4* (*LCL1*) | AT5G02840 | 1.7462 | -1.9522 | Dawn | Promotes expression of clock genes with evening element (partially redundant with RVE6 and RVE8) (Hsu et al., 2013) |
| *RVE7* | AT1G18330 | 2.0563 | -2.4899 | Midday | Promotes seed dormancy and reduces germination through interaction with clock genes (Liu et al., 2021b) |
| *ELF4* | AT2G40080 | -3.3912 | 1.5934 | Evening | Forms Evening Complex (EC) and represses expression of PRR and output genes (Nusinow et al., 2011; Huang & Nusinow, 2016) |
| *LUX* (*PCL1*) | AT3G46640 | -2.1206 | 1.5496 | Evening | Forms EC to repress expression of PRR and output genes and inhibits seed dormancy via repressing DOG1 (Helfer et al., 2011; Zha et al., 2020) |
| *phyE* | AT4G18130 | -1.5474 | 1.7538 | Morning | Promote seed germination via sensing seed sensitivity to the temperature and the ratio of red to far-red light altered during maturation (Goosey et al., 1997; Dechaine et al., 2009) |
| *phyD* | AT4G16250 | -2.0959 | 1.7798 | Evening and night | Positively controls germination via sensing seed sensitivity to the temperature and the ratio of red to far-red light altered during maturation (Goosey et al., 1997; Dechaine et al., 2009) |
| *PIF5* (*PIL6*) | AT3G59060 | 1.5044 | -1.5147 | Dawn | Circadian-regulated negative regulator of photomorphogenesis (Leivar et al., 2008) |
| *PIF6* (*PIL2*) | AT3G62090 | -3.3594 | 1.9858 | Afternoon/Evening | Decreases seed dormancy in response to light strength experienced by seed during maturation (Penfield et al., 2010). |
| *LNK2* | AT3G54500 | 1.5128 | -1.5383 | Morning | Negatively regulates the period length of circadian rhythm. Transcriptional coactivator of afternoon genes. Plays a role in red light input to the clock (Rugnone et al., 2013; Xie et al., 2014). |
| *PKS2* | AT1G14280 | 1.8644 | -8.4308 | Dawn | Plays a role for normal phototropic response (Kami et al., 2014). |
| *NOX* (*BOA*) | AT5G59570 | -2.4168 | 1.5011 | Evening | Transcriptional activator of its own repressor CCA1 and regulates output genes (Dai et al., 2011; Helfer et al., 2011) |
| *GRP7* | AT2G21660 | -2.5409 | 1.6504 | Evening | Negatively auto-regulated clock gene; accelerates seed germination under low-temperature conditions (Schmal et al., 2013) |
| *GRP8* | AT4G39260 | -1.9385 | 1.5469 | Evening | Negatively auto-regulated clock gene repressed by morning-phased genes LHY/CCA1 and cross-regulated with GRP7 (Schmal et al., 2013) |
| *bZIP63* | AT5G28770 | 1.9159 | -1.5928 | Dawn | Regulates a response of the circadian oscillator to Sugars; negatively regulates [seed germination](https://www.sciencedirect.com/topics/agricultural-and-biological-sciences/seed-germination) upon osmotic stress (Veerabagu et al., 2014; Frank et al., 2018). |
| *GCN* (*GATA21*) | AT5G56860 | 1.8939 | -2.1805 | Night | Regulated by circadian and GA, repressed by PIFs, and negatively regulates germination (Richter et al., 2013) |
| *JMJD5* (*JMJ30*) | AT3G20810 | -3.0548 | 2.8395 | Evening | Circadian-regulated histone demethylase repressed by CCA1 and LHY (Jones et al., 2019). |
| *ERD7* | AT2G17840 | -2.0717 | 1.5419 | Evening | High light, circadian and abiotic stress-regulated protein (Mizuno and Yamashino, 2008) |
| *COR27* | AT5G42900 | -2.4898 | 2.4709 | Evening | Nighttime repressor repressed by CCA1 and integrating circadian clock and plant cold stress responses (Li et al., 2020) |
| *FKF1* (ADO3/WRKY63) | AT1G68050 | -2.3770 | 1.8747 | Evening | Circadian-regulated protein interacting with GI; Involved in the regulation of plant responses to ABA and abiotic stress (Li et al., 2013) |
| *BBX2* (*COL1*) | AT5G15850 | 1.8561 | -2.2091 | Dawn | Positively regulates the period length of circadian rhythm as overexpression of *BBX2* can accelerate circadian clock and shorten the period (Ledger et al., 2001). |
| *BBX3* (*COL2*) | AT3G02380 | 3.3327 | -2.8922 | Dawn | Positively regulates the period length of circadian rhythm (Ledger et al., 2001). |
| *BBX6* (*COL5*) | AT5G57660 | 1.5435 | -1.7622 | Dawn/ Morning | Circadian-regulated protein expressed in vascular tissues and regulated by GI (Hassidim et al., 2009) |
| *BBX7* (*COL9*) | AT3G07650 | -2.4183 | 1.5821 | Evening | Positively regulates blue light-dependent freezing tolerance and abiotic stress (Li et al., 2021) |
| *BBX8* (*COL10*) | AT5G48250 | -1.5362 | 1.5487 | Evening | Positively regulates blue light-dependent freezing tolerance. Negatively regulated by CCA1 (Kamioka et al., 2016; Li et al., 2021) |
| *BBX13* (*COL15*) | AT1G28050 | -2.4927 | 1.7978 | Evening | Negatively regulated by CCA1 (Kamioka et al., 2016) |
| *BBX16* (*COL7*) | AT1G73870 | 3.1601 | -2.6726 | Dawn | Circadian-regulated negative regulator of photomorphogenesis (Veciana et al., 2022) |
| *BBX19* | AT4G38960 | 2.3420 | -2.4365 | Dawn | Positively regulates the period length of circadian rhythm and seed dormancy. Negatively regulates morning-phased clock genes and photomorphogenesis (Bai et al., 2019; Yuan et al., 2021) |
| *BBX21* (*STH2*) | AT1G75540 | -1.5232 | 1.5611 | Night | Circadian-regulated positive regulator of photomorphogenesis; positively regulates germination in ABA (Xu et al., 2014) |
| *BBX22* (*LZF1*/*STH3*) | AT1G78600 | -1.7919 | 1.5270 | Evening | Circadian-regulated positive regulator of photomorphogenesis, Regulates cell wall remodeling genes (Datta et al., 2007; Chang et al., 2011) |
| *BBX25* | AT2G31380 | 1.5985 | -3.0573 | Dawn | Circadian-regulated negative regulator of photomorphogenesis *via* downregulating BBX22 (Gangappa et al., 2013) |
| *SOM* | AT1G03790 | -3.3995 | 2.6505 | Night | Circadian-regulated high temperature-activated negative regulator of seed germination at high temperature (Lim et al., 2013). |
| *PCH1* | AT2G16365 | -1.6932 | 1.5130 | Evening | phyD and phyE-associated positive regulator of plant photomorphogenesis and integrator of light and temperature for the control of photomorphogenesis (Huang et al., 2019). |

**References**

Frank, A., Matiolli, C. C., Viana, A. J., et al. 2018. Circadian entrainment in Arabidopsis by the sugar-responsive transcription factor bZIP63. *Current Biology* 28: 2597-2606. e2596.

Gangappa, S. N. and Botto, J. F. 2014. The BBX family of plant transcription factors. *Trends in Plant Science* 19: 460-470.

Gangappa, S. N., Crocco, C. D., Johansson, H., Datta, S., Hettiarachchi, C., Holm, M. and Botto, J. F. 2013. The Arabidopsis B-BOX protein BBX25 interacts with HY5, negatively regulating BBX22 expression to suppress seedling photomorphogenesis. *The Plant Cell* 25: 1243-1257.

Hassidim, M., Harir, Y., Yakir, E., Kron, I. and Green, R. M. 2009. Over-expression of CONSTANS-LIKE 5 can induce flowering in short-day grown Arabidopsis. *Planta* 230: 481-491.

Huang, H., McLoughlin, K. E., Sorkin, M. L., Burgie, E. S., Bindbeutel, R. K., Vierstra, R. D. and Nusinow, D. A. 2019. PCH1 regulates light, temperature, and circadian signaling as a structural component of phytochrome B-photobodies in Arabidopsis. *Proceedings of the National Academy of Sciences* 116: 8603-8608.

Jones, M. A., Morohashi, K., Grotewold, E. and Harmer, S. L. 2019. Arabidopsis JMJD5/JMJ30 acts independently of LUX ARRHYTHMO within the plant circadian clock to enable temperature compensation. *Frontiers in Plant Science* 10: 427192.

Kamioka, M., Takao, S., Suzuki, T., Taki, K., Higashiyama, T., Kinoshita, T. and Nakamichi, N. 2016. Direct Repression of Evening Genes by CIRCADIAN CLOCK-ASSOCIATED1 in the Arabidopsis Circadian Clock. *The Plant Cell* 28: 696-711.

Ledger, S., Strayer, C., Ashton, F., Kay, S. A. and Putterill, J. 2001. Analysis of the function of two circadian‐regulated CONSTANS‐LIKE genes. *The Plant Journal* 26: 15-22.

Li, F., Zhang, X., Hu, R., Wu, F., Ma, J., Meng, Y. and Fu, Y. 2013 Identification and molecular characterization of FKF1 and GI homologous genes in soybean. *PloS One* 8: e79036.

Li, X., Liu, C., Zhao, Z., Ma, D., Zhang, J., Yang, Y., Liu, Y. and Liu, H. 2020. COR27 and COR28 are novel regulators of the COP1–HY5 regulatory hub and photomorphogenesis in Arabidopsis. *The Plant Cell* 32: 3139-3154.

Li, Y., Shi, Y., Li, M., Fu, D., Wu, S., Li, J., Gong, Z., Liu, H. and Yang, S. 2021. The CRY2–COP1–HY5–BBX7/8 module regulates blue light-dependent cold acclimation in Arabidopsis*. The Plant Cell* 33: 3555-3573.

Lim, S., Park, J., Lee, N., et al. 2013. ABA-INSENSITIVE3, ABA-INSENSITIVE5, and DELLAs interact to activate the expression of SOMNUS and other high-temperature-inducible genes in imbibed seeds in Arabidopsis. *The Plant Cell* 25: 4863-4878.

Mizuno, T. and Yamashino, T. 2008. Comparative transcriptome of diurnally oscillating genes and hormone-responsive genes in Arabidopsis thaliana: insight into circadian clock-controlled daily responses to common ambient stresses in plants. *Plant and Cell Physiology* 49: 481-487.

Ren, H., Park, M..Y., Spartz, A..K., Wong, J. H. and Gray, W. M. 2018. A subset of plasma membrane-localized PP2C. D phosphatases negatively regulate SAUR-mediated cell expansion in Arabidopsis. *PLoS Genetics* 14: e1007455.

Veciana, N., Martín, G., Leivar, P. and Monte, E. 2022. BBX16 mediates the repression of seedling photomorphogenesis downstream of the GUN1/GLK1 module during retrograde signalling. *New Phytologist* 234: 93-106.

Xu, D., Li, J., Gangappa, S. N., et al. 2014. Convergence of light and ABA signaling on the ABI5 promoter. *PLoS Genetics* 10: e1004197.

Yuan, L., Yu, Y., Liu, M., et al. 2021. BBX19 fine-tunes the circadian rhythm by interacting with PSEUDO-RESPONSE REGULATOR proteins to facilitate their repressive effect on morning-phased clock genes. *The Plant Cell* 33: 2602-2617.
