# Supplementary material for: SCL15 Regulates the Release of Seed Dormancy in Arabidopsis thaliana by Integrating the Circadian Clock, Hormonal Signals and Cell Wall Remodelling
Source: Physiol Plant. 2025 Sep 8;177(5):e70467. doi: 10.1111/ppl.70467 (PMC12415678; doi:10.1111/ppl.70467)
Supplement: Supplementary file 5 — Data S2: ppl70467‐sup‐0005‐Figures.docx. Figure S1: qRT‐PCR analysis of SCL15 expression in leaves of 4‐week old seedlings (left) or siliques 14 days post‐anthesis (right) of wild type A. thaliana Col‐0, scl15‐1, Napin:SCL15 and 35S:SCL15 plants. SCL15 RNA levels in the wild type Col‐0 were designated as one‐fold. Figure S2: Principal component analysis (PCA) of global gene expression data in Arabidopsis seeds of wild‐type Col‐0 (WT), scl15‐1 mutant and Napin:SCL15 overexpression lines. Three biological replicates for each line are indicated as rep1, rep2 and rep3. Figure S3: Validation of RNAseq data with real‐time qRT‐PCR. Genes that are downregulated in scl15‐1 and upregulated in the Napin:SCL15 lines are selected for expression analysis. Gene IDs: RAB18, AT5G66400; EM1, AT3G51810; EM6, AT2G40170; LEA18, AT2G35300; M10, AT2G41280; LEA4‐5, AT5G06760; XERO1, AT3G50980; IAA34, AT1G15050; ABI5, AT2G36270; HAI3, AT2G29380; PIF6, AT3G62090; HSP26.5, AT1G52560; HSP70‐5, AT1G16030; HSP17.4A, AT3G46230; HSP70‐8, AT2G32120; HSP17.6C, AT1G53540; PIMT2, AT5G50240; HVA22B, AT5G62490; MSH3, AT4G25540; MSD2, AT3G56350; COR27, AT5G42900; DREB2E, AT2G38340; DREB2G, AT5G18450; MYB90, AT1G66390; MYB21, AT3G27810; ABR1, AT5G64750; DOG1, AT5G45830; ATECP31, AT3G22500; ATRAB28, AT1G03120; OLEO4, AT3G27660; OLEO1, AT4G25140. Figure S4: The pattern of expression across different physiological states for selected CW‐related genes in Arabidopsis seeds. (A) Expression of wall genes that are upregulated in scl15‐1 and downregulated in Napin:SCL15. Note that these genes showed higher levels of expression in the imbibed, after‐ripened (AR) state without germination (DL) compared with the imbibed and germinated AR state (LIG) or any of the dormant states, indicating a role in dormancy maintenance. (B) Expression patterns of wall‐related genes that are downregulated in scl15‐1 and upregulated in Napin:SCL15. Note that these genes displayed higher levels of expression during germination inductio [file PPL-177-e70467-s001.docx]

**SUPPLEMENTAL FIGURES**

**SCL15 regulates the release of seed dormancy in *Arabidopsis thaliana* by integrating the circadian clock, hormonal signals, and cell wall remodeling**

Ming-Jun Gao^1,*^, Qi Chen^2^, Cathy Coutu^1^, Fuyou Fu^1^, Bianyun Yu^1^, Xiang Li^1^, Z Jeffrey Chen^3^, and Dwayne Hegedus^1,*^

^1^Agriculture and Agri-Food Canada, Saskatoon Research and Development Centre, 107 Science Place, Saskatoon, S7N 0X2, SK, Canada

^2^State Key Laboratory of Tea Plant Biology and Utilization, Anhui Agricultural University, Hefei, Anhui, China

^3^Department of Molecular Biosciences, The University of Texas at Austin, Austin, TX 78712, USA

***Corresponding authors:** Dwayne Hegedus, [Dwayne.hegedus@agr.gc.ca](mailto:Dwayne.hegedus@agr.gc.ca); Ming-Jun Gao, [ming-jun.gao@agr.gc.ca](mailto:ming-jun.gao@agr.gc.ca)


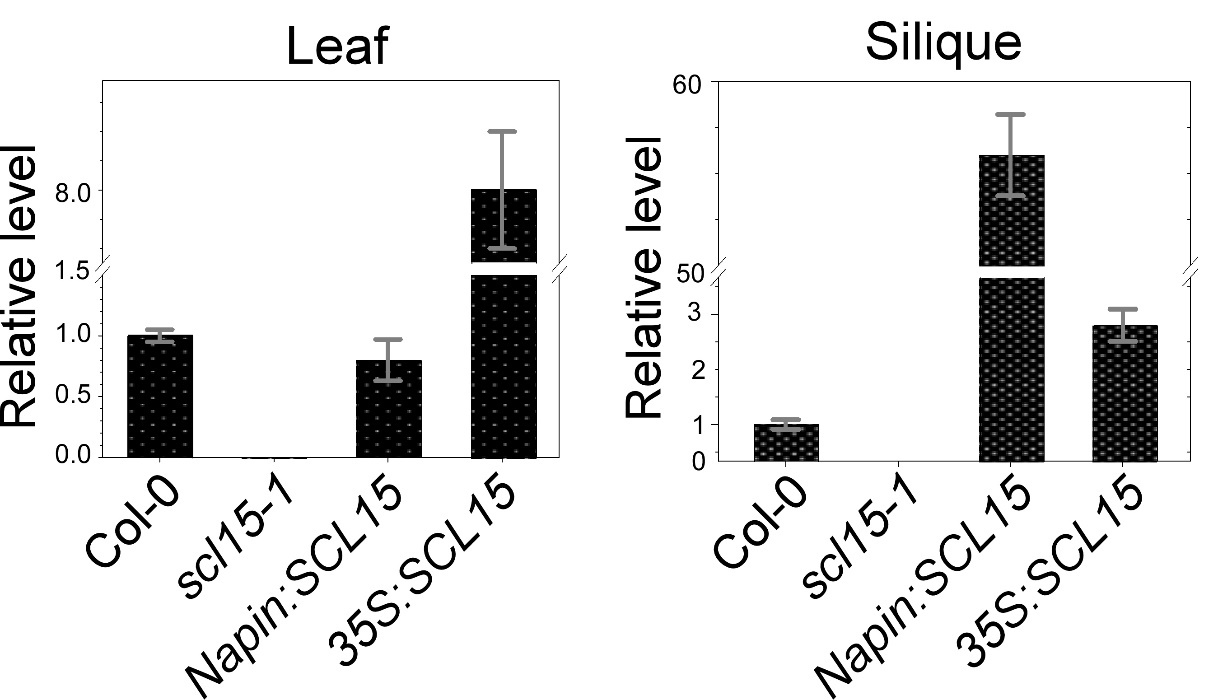


**Figure S1.** qRT-PCR analysis of *SCL15* expression in leaves of 4-week old seedlings (left) or siliques 14 days post-anthesis (right) of wild type *A. thaliana* Col-0, *scl15-1*, Napin:SCL15 and 35S:SCL15 plants. SCL15 RNA levels in the wild type Col-0 were designated as one-fold.


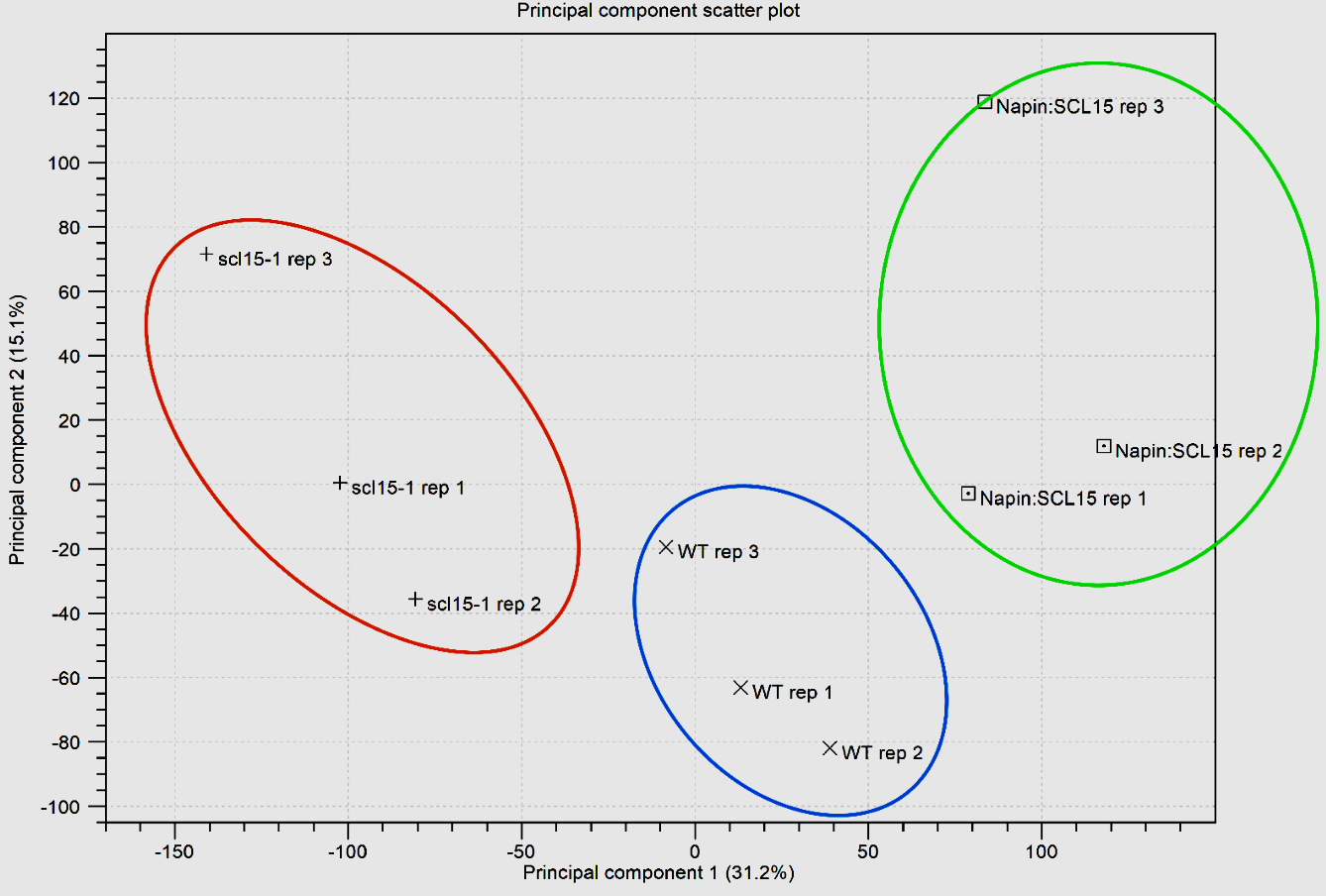


**Figure S2.** Principal component analysis (PCA) of global gene expression data in Arabidopsis seeds of wild-type Col-0 (WT), *scl15-1* mutant and Napin:SCL15 overexpression lines. Three biological replicates for each line are indicated as rep1, rep2 and rep3.


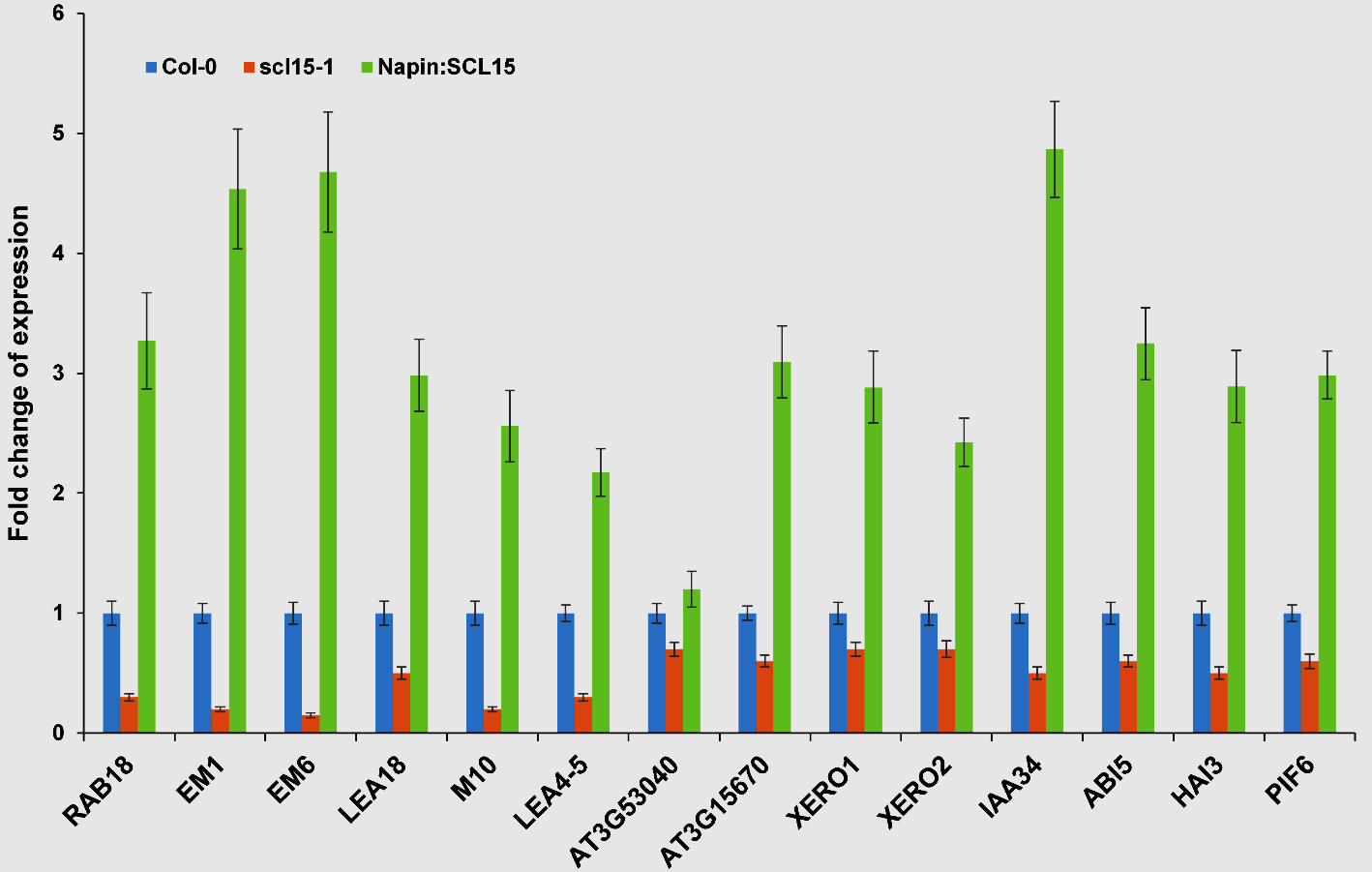


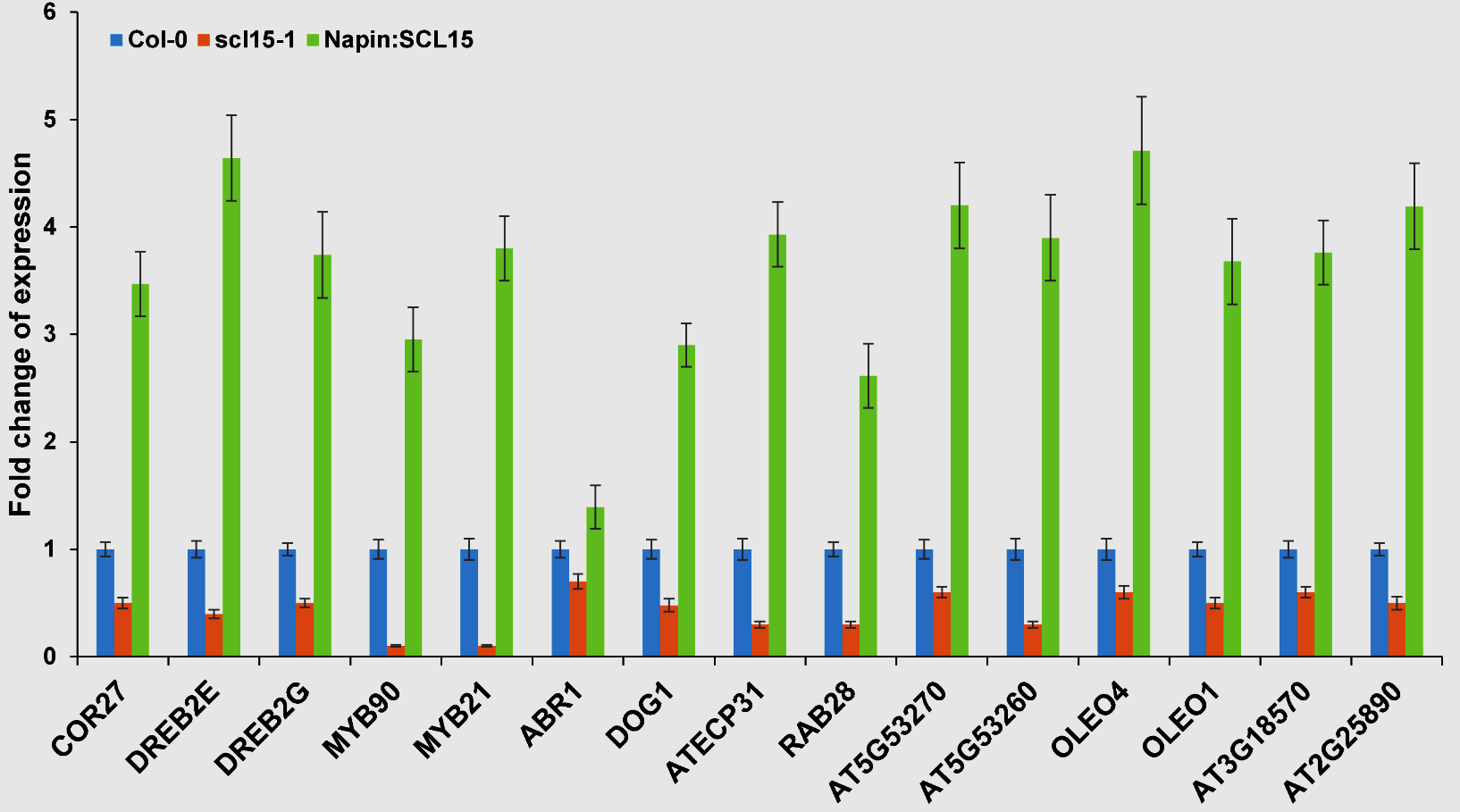


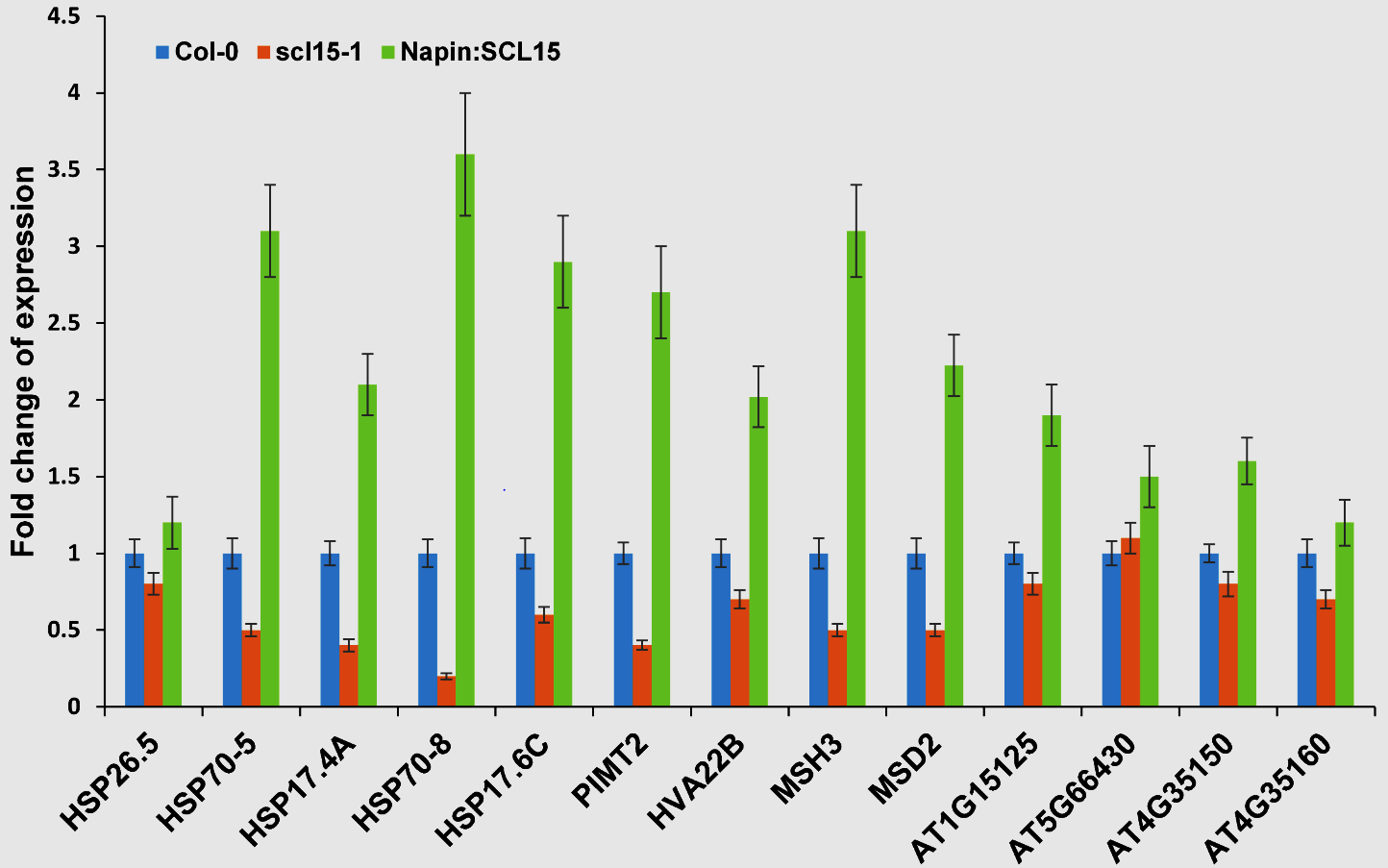


**Figure S3.** Validation of RNAseq data with real-time qRT-PCR. Genes that are downregulated in *scl15-1* and upregulated in the Napin:SCL15 lines are selected for expression analysis. Gene IDs: RAB18, AT5G66400; EM1, AT3G51810; EM6, AT2G40170; LEA18, AT2G35300; M10, AT2G41280; LEA4-5, AT5G06760; XERO1, AT3G50980; IAA34, AT1G15050; ABI5, AT2G36270; HAI3, AT2G29380; PIF6, AT3G62090; HSP26.5, AT1G52560; HSP70-5, AT1G16030; HSP17.4A, AT3G46230; HSP70-8, AT2G32120; HSP17.6C, AT1G53540; PIMT2, AT5G50240; HVA22B, AT5G62490; MSH3, AT4G25540; MSD2, AT3G56350; COR27, AT5G42900; DREB2E, AT2G38340; DREB2G, AT5G18450; MYB90, AT1G66390; MYB21, AT3G27810; ABR1, AT5G64750; DOG1, AT5G45830; ATECP31, AT3G22500; ATRAB28, AT1G03120; OLEO4, AT3G27660; OLEO1, AT4G25140.


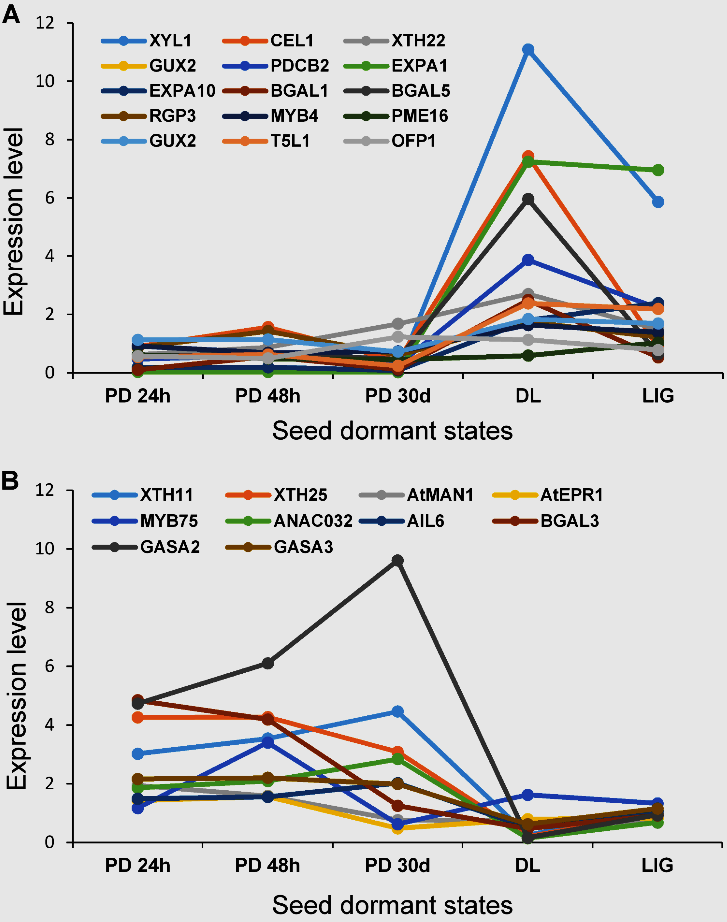


**Figure S4.** The pattern of expression across different physiological states for selected CW-related genes in Arabidopsis seeds. (**A**) Expression of wall genes that are upregulated in *scl15-1* and downregulated in Napin:SCL15. Note that these genes showed higher levels of expression in the imbibed, after-ripened (AR) state without germination (DL) compared with the imbibed and germinated AR state (LIG) or any of the dormant states, indicating a role in dormancy maintenance. (**B**) Expression patterns of wall-related genes that are downregulated in *scl15-1* and upregulated in Napin:SCL15. Note that these genes displayed higher levels of expression during germination induction in imbibed dormant states compared with imbibed AR states, indicating a positive role in the initiation of germination and dormancy release. For the treatment of dormant imbibed state, PD 24h, primary dormant seeds were imbibed for 24h (will not complete germination). PD 48h, primary dormant seeds were imbibed for 24h (will not complete germination). PD 30d, primary dormant seeds were imbibed for 30 days in the dark, a dormant imbibed state. For the treatment of imbibed after-ripened state, DL, dry seeds were after-ripened for 120 days and then imbibed for 24h (will germinate if placed in the light). LIG, dry seeds were after-ripened for 120 days and then imbibed for 20h in the dark, and then place for 4h under red light to terminate dormancy and induce germination (will complete germination) (Cadman et al., 2006). Data are derived from the *Arabidopsis* eFP Browser (<http://bar.utoronto.ca/efp/cgi-bin/efpWeb.cgi>).


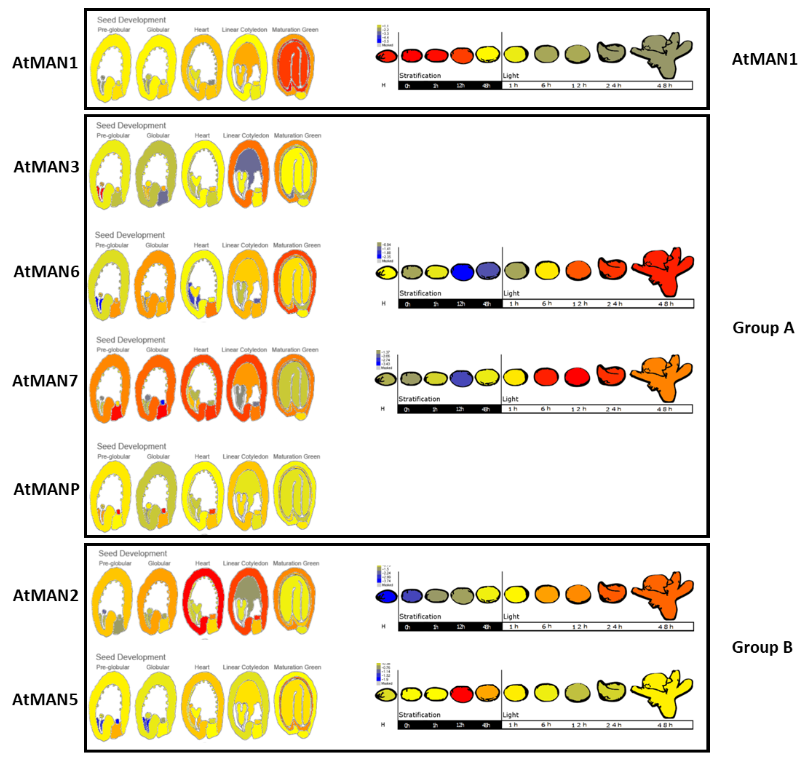


**Figure S5.** Expression of endo-β-mannanases (MANs) in developing seeds and during germination showing the unique expression patterns for MAN1/AtMAN1. Data are derived from the Arabidopsis eFP Browser. Noted that compared with the other seven members of the *A. thaliana* MAN protein family, *MAN1/AtMAN5-1* was the most highly expressed MAN gene in the endosperm and its expression was highest in the ME during stratification and initiation of seed germination, suggesting that MAN1 plays a role in the modulation of seed dormancy maintenance and dormancy release.


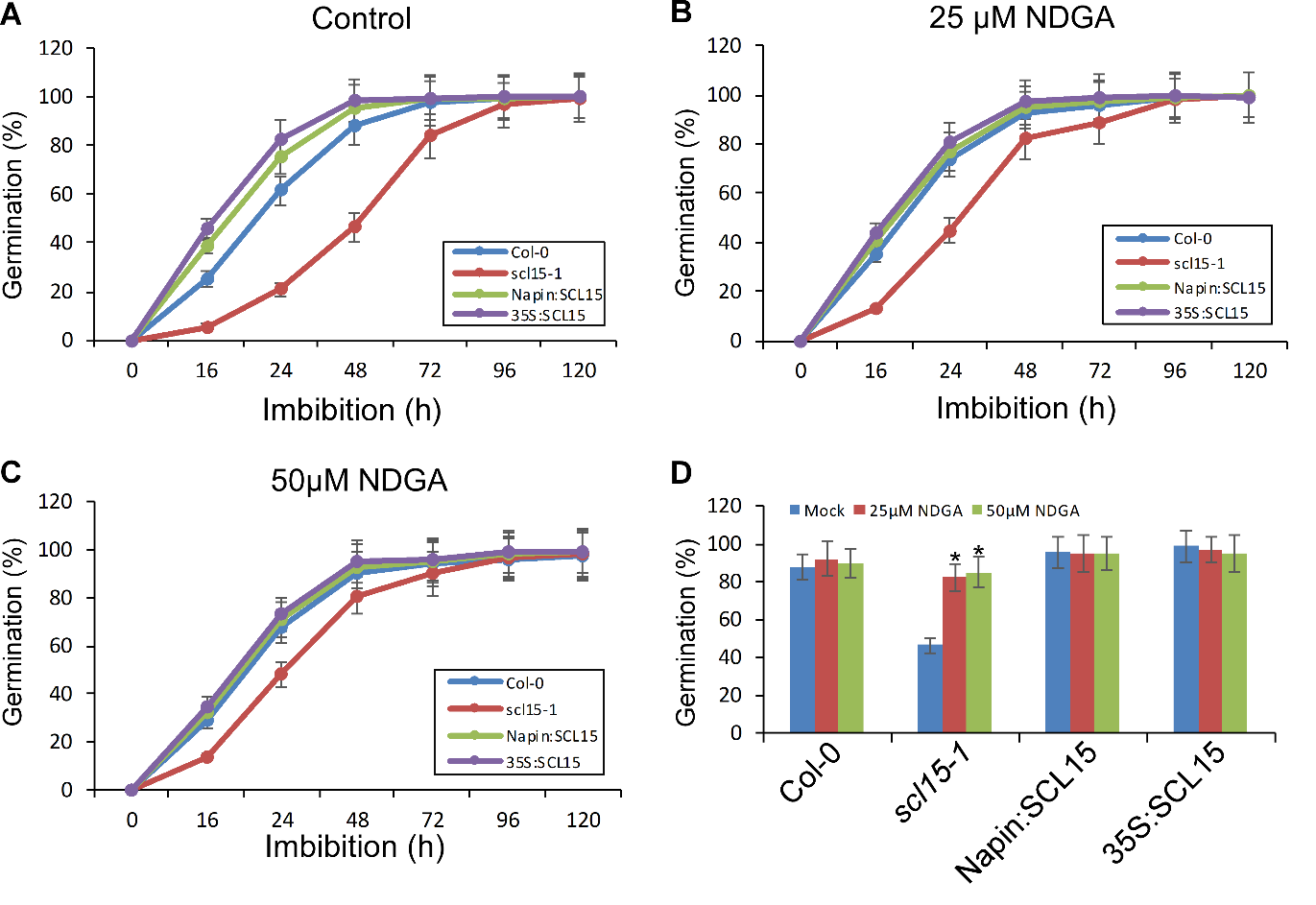


**Figure S6**. Seed germination of mutant *scl15-1* and *SCL15*-overexpression lines Napin:SCL15 and 35S:SCL15 and their response to ABA biosynthesis inhibitor NDGA. (A) to (C) Germination of wild type Col-0, *scl15-1*, Napin:SCL15 and 35S:SCL15 in response to NDGA treatment. Stratified seeds were sown onto ½ MS agar medium supplemented with 0 (control) (A), 25 (B) or 50 µm (C) NDGA. (D) Enhancement of germination speed of *scl15-1* in response to NDGA after imbibition for 48 h. Percentages of seed germination are means (±SD) from four biological replicates. Asterisks in (D) indicate a statistically significant difference between control and 25 or 50 µm NDGA treatment (**p*<0.05; Student's t test).


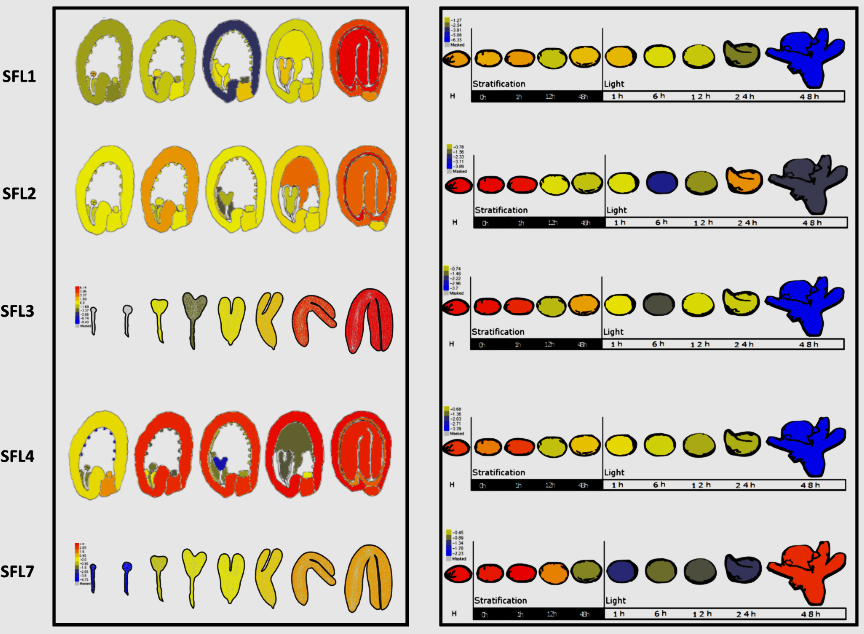


**Figure S7.** Expression profiles of genes encoding SEED DORMANCY FOUR-LIKE (SFL) family proteins in developing seeds and during germination. Data are derived from the Arabidopsis eFP Browser (<http://bar.utoronto.ca/efp/cgi-bin/efpWeb.cgi>).


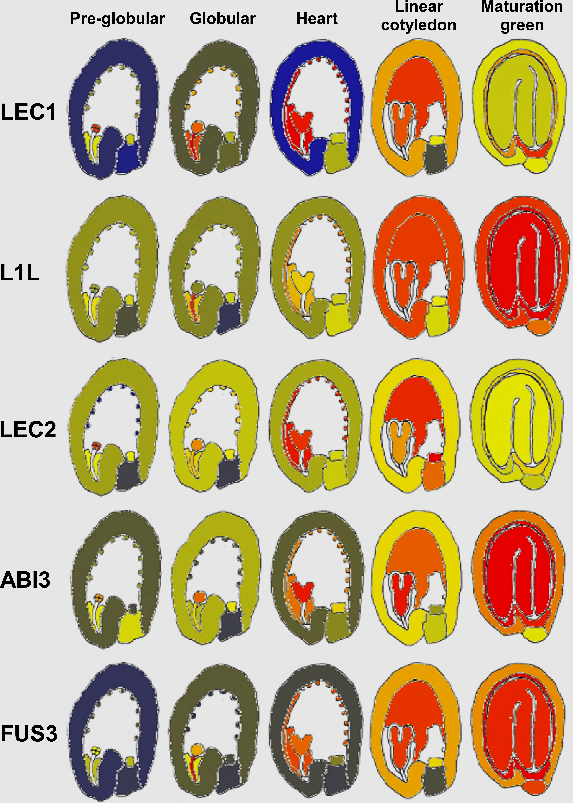


**Figure S8.** Expression patterns of *LAFL* genes (*LEC1*, *LEC2*, *ABI3*, and *FUS3*) and *L1L* in developing seeds showing specific transcript accumulation of *LEC1* in the endosperm of mature seed. Data are derived from the *Arabidopsis* eFP Browser (<http://bar.utoronto.ca/efp/cgi-bin/efpWeb.cgi>).


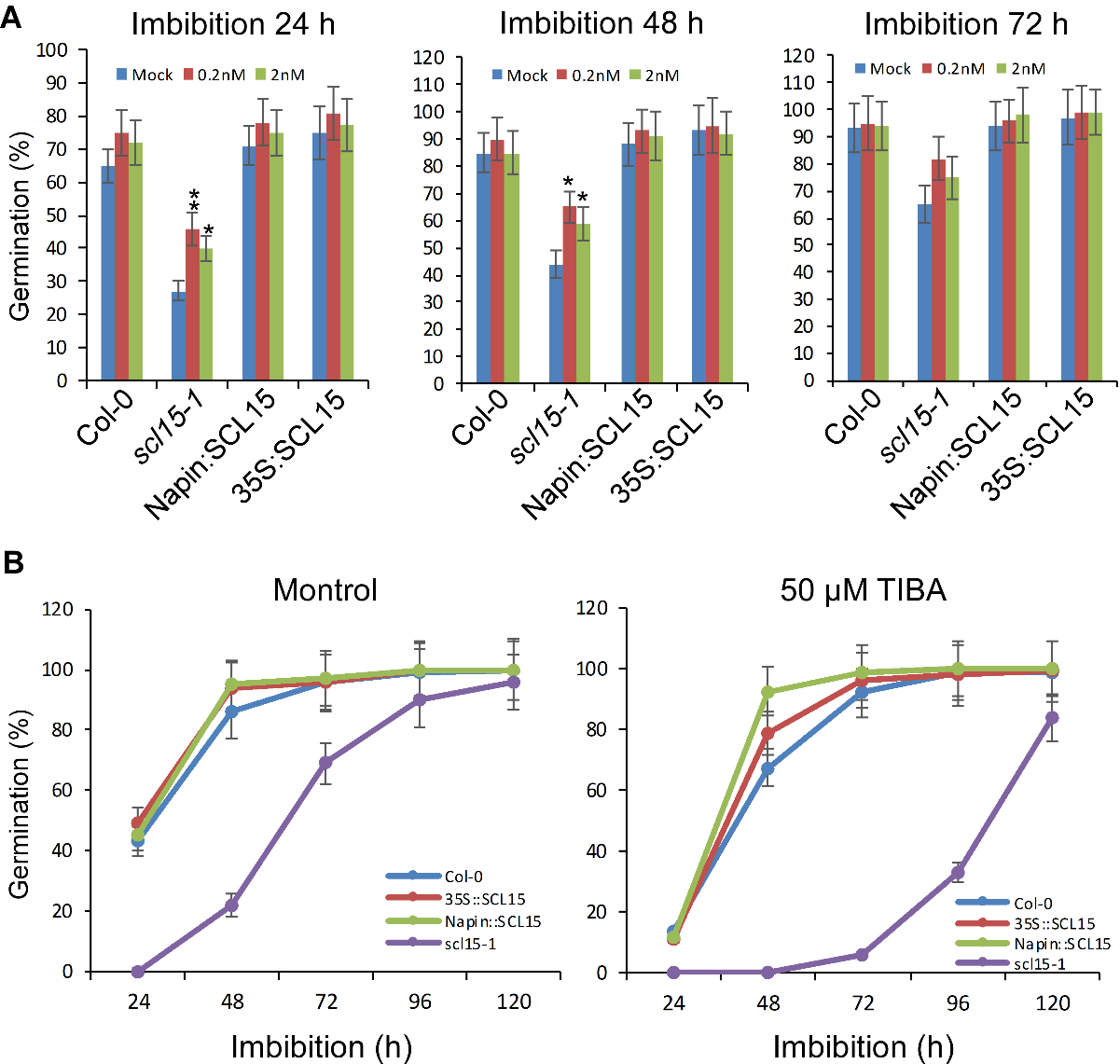


**Figure S9.** Germination of *scl15-1*, Napin:SCL15 and 35S:SCL15 seeds in response to 2,4-D and the auxin biosynthetic inhibitor TIBA. (A) Germination of wild type Col-0, *scl15-1*, Napin:SCL15 and 35S:SCL15 in response to 2,4-D treatment after imbibition for 24 h, 48 h or 72 h. Stratified seeds were sown onto ½ MS agar medium supplemented with 0, 0.2 or 2 nM 2,4-D. Percentages of seed germination are means (±SD) of four biological replicates. Asterisks indicates a statistically significant difference between the control and 2,4-D treatment (*p*<0.05; Student's t test). (B) Inhibition of germination of *scl15-1* in response to TIBA. Seeds were sown on ½ MS medium supplemented with 0 or 50 μM TIBA. Percentages of seed germination are means (±SD) from four biological replicates. Statistically significant differences between the control and 50 µM TIBA treatment were determined using a Student's t test (*p*<0.01).


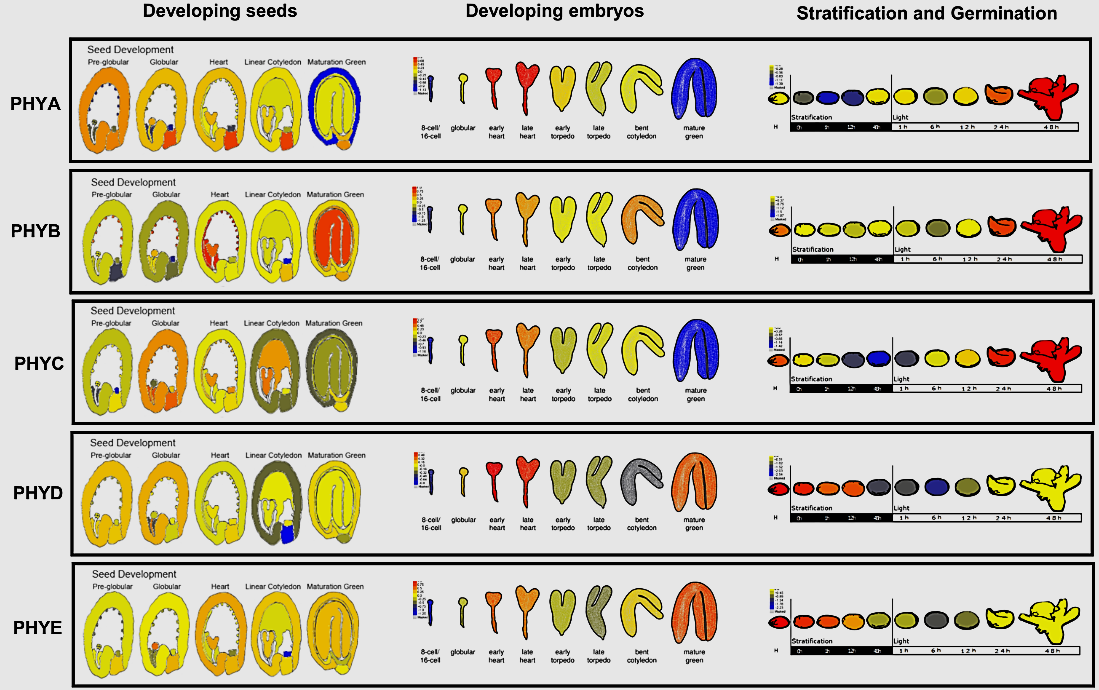


**Figure S10**. Expression of phytochrome (PHY) *PhyA, PhyB*, *PhyD* and *PhyE* in developing seeds, embryos and during germination. Data are derived from the Arabidopsis eFP Browser (<http://bar.utoronto.ca/efp/cgi-bin/efpWeb.cgi>).


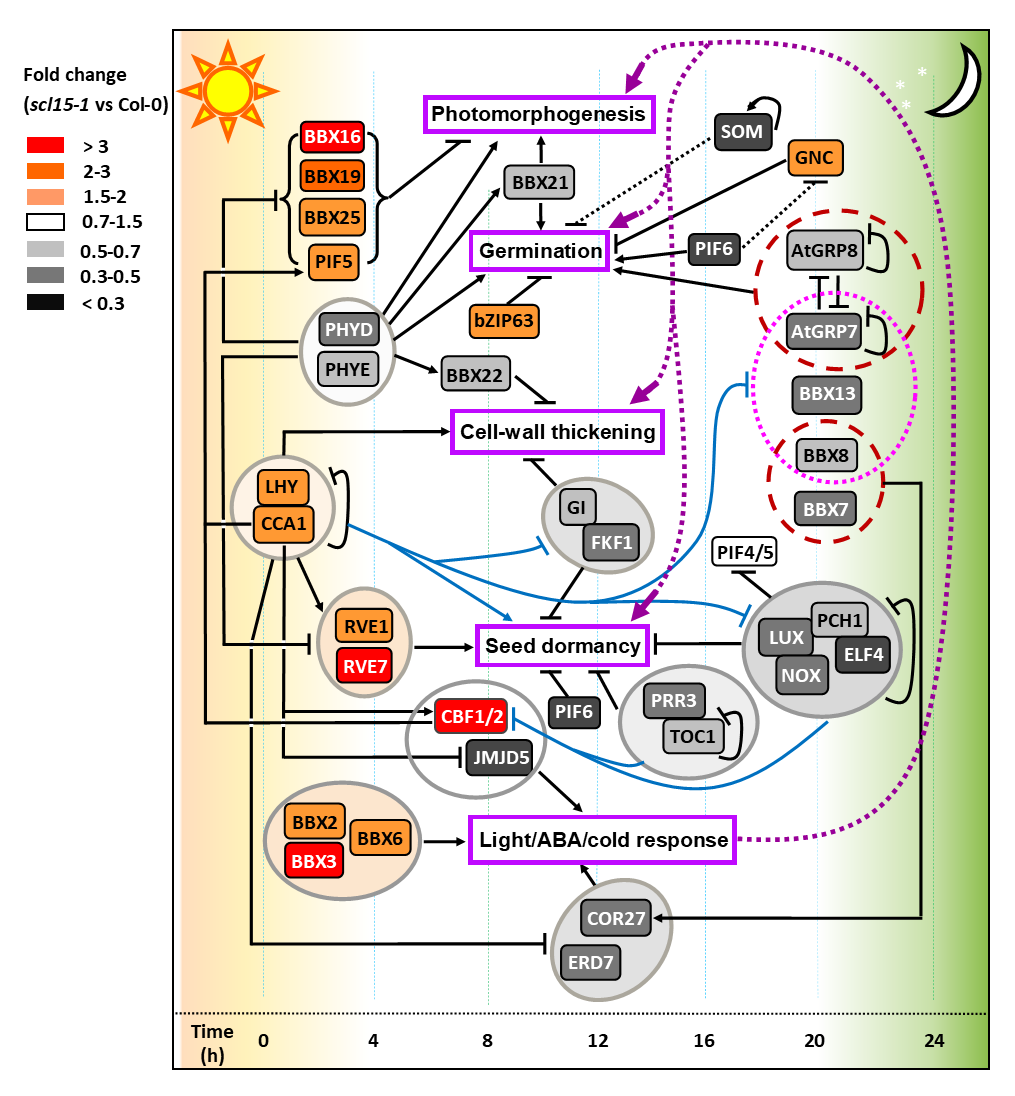


**Figure S11.** Changes in expression of circadian-regulated genes in maturing *scl15-1* seeds. BBX, B-BOX DOMAIN PROTEIN; bZIP63; BASIC LEUCINE ZIPPER63; COR27, cold regulated gene 27; ERD7, EARLY-RESPONSIVE TO DEHYDRATION 7; GNC, GATA, NITRATE-INDUCIBLE, CARBON METABOLISM INVOLVED; GRP, glycine-rich RNA-binding protein; JMJD5, JUMONJI DOMAIN CONTAINING 5; PHYD, phytochrome D; PHYE, phytochrome D; PIF, PHYTOCHROME INTERACTING FACTOR 3-LIKE; SOM, SOMNUS; Other abbreviations are in Figure **10**.


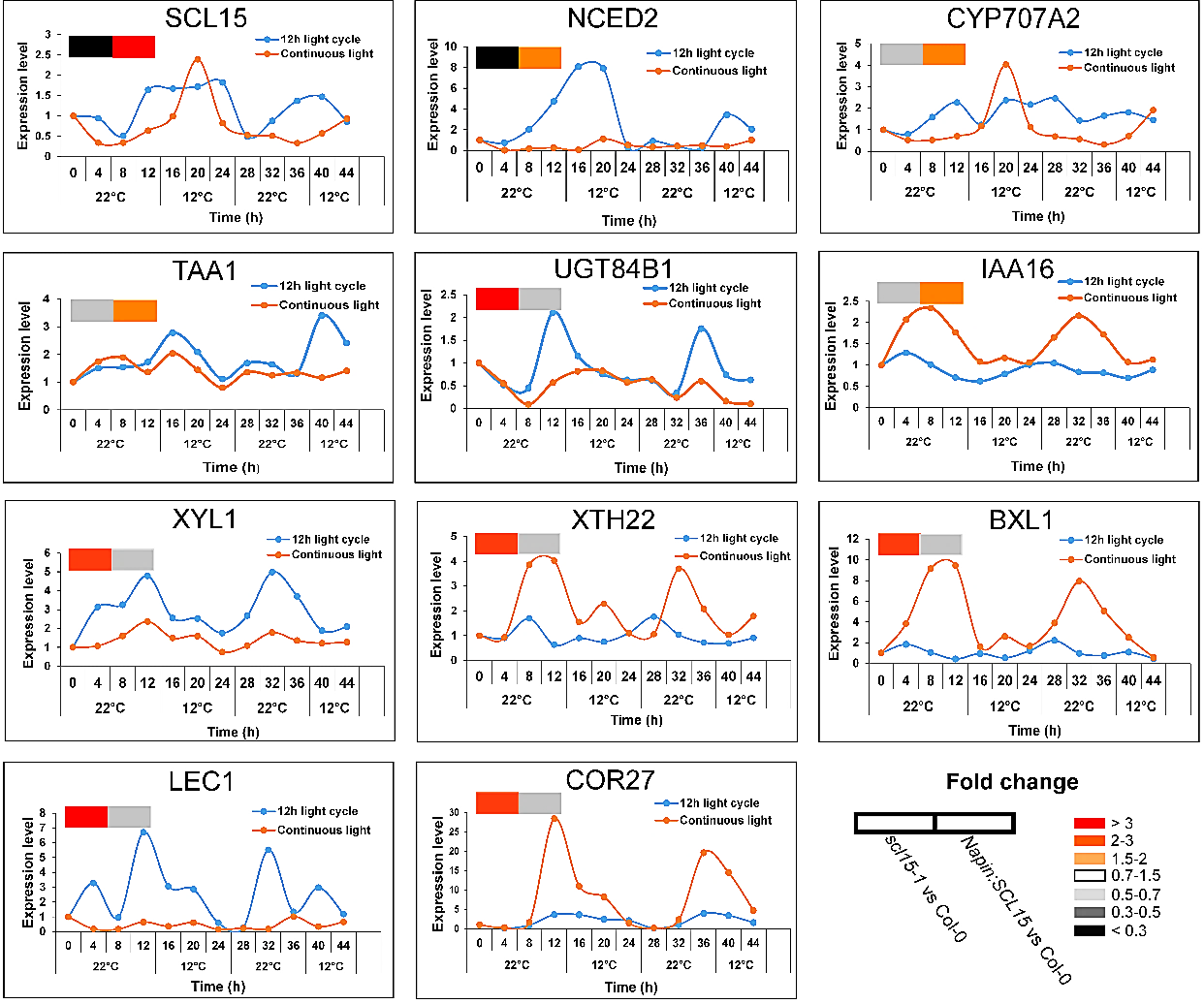


**Figure S12.** Rhythmic expression of *SCL15* and selected genes that are regulated by SCL15 and involved in ABA and auxin signalling or cell wall remodelling. Changes in expression of circadian-regulated genes in maturing *scl15-1* seeds are derived from the *Arabidopsis* eFP Browser (<http://bar.utoronto.ca/efp/cgi-bin/efpWeb.cgi>).


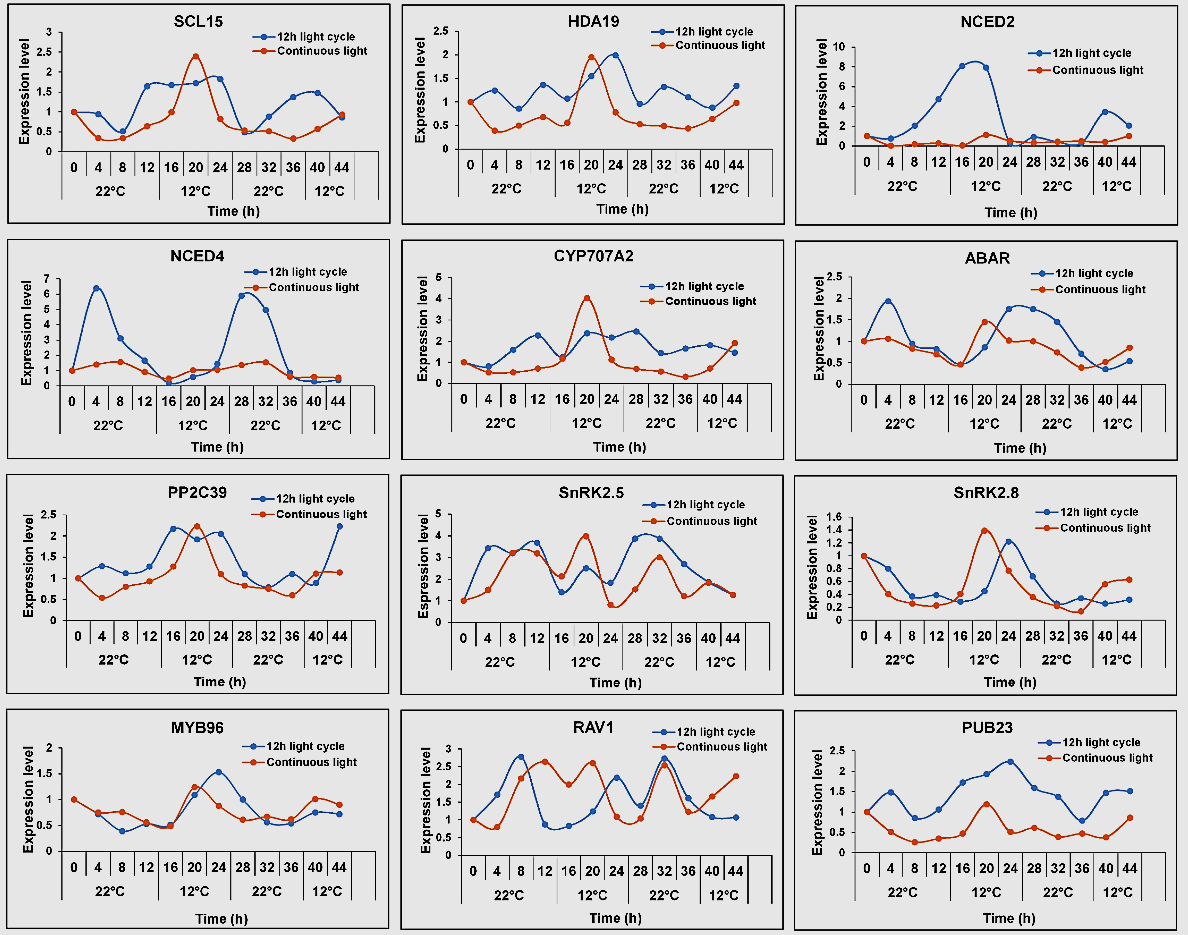


**Figure S13.** Rhythmic expression of genes that are regulated by SCL15 and involved in ABA biosynthesis, metabolism and signaling. Data are derived from the *Arabidopsis* eFP Browser (<http://bar.utoronto.ca/efp/cgi-bin/efpWeb.cgi>).


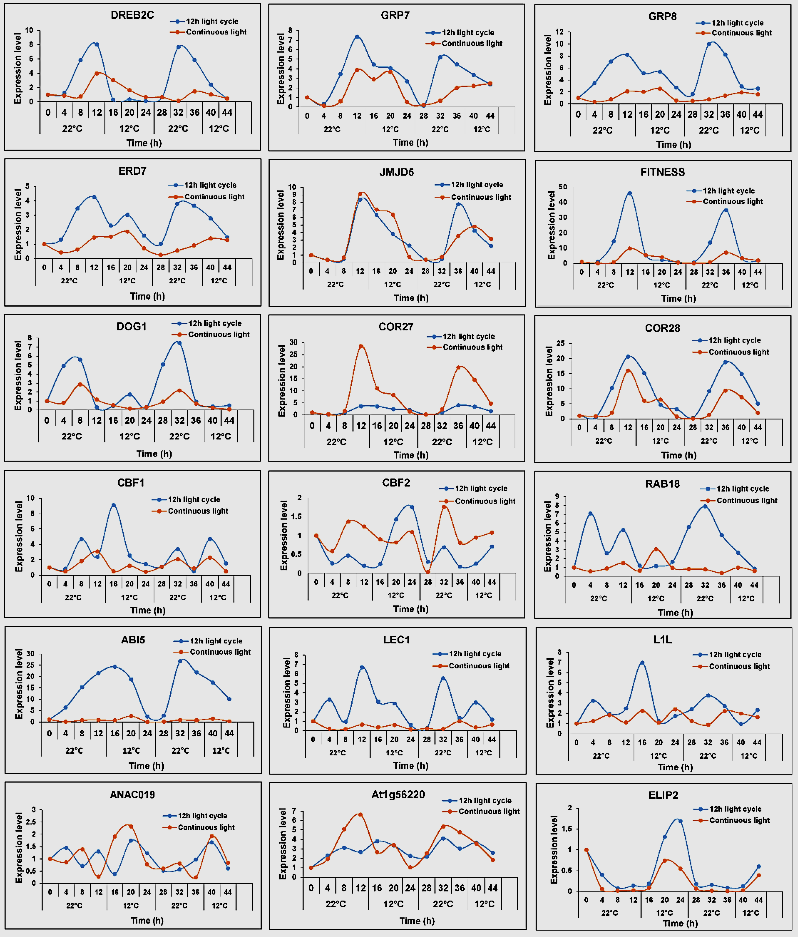


**Figure S14.** Rhythmic expression of genes that are regulated by SCL15 and responsive to ABA. Data are derived from the *Arabidopsis* eFP Browser (<http://bar.utoronto.ca/efp/cgi-bin/efpWeb.cgi>).


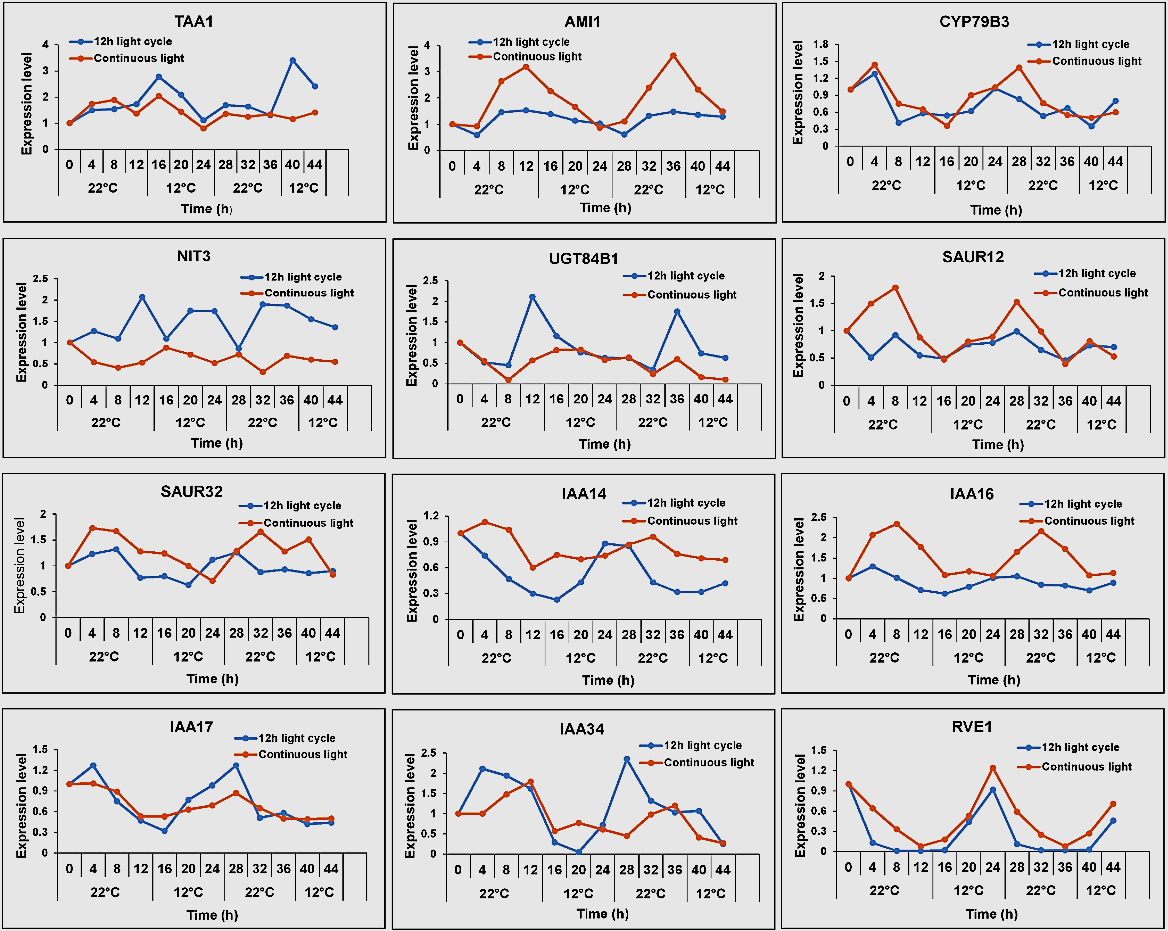


**Figure S15.** Rhythmic expression of genes that are regulated by SCL15 and involved in auxin metabolism and signaling. Data are derived from the *Arabidopsis* eFP Browser (<http://bar.utoronto.ca/efp/cgi-bin/efpWeb.cgi>).


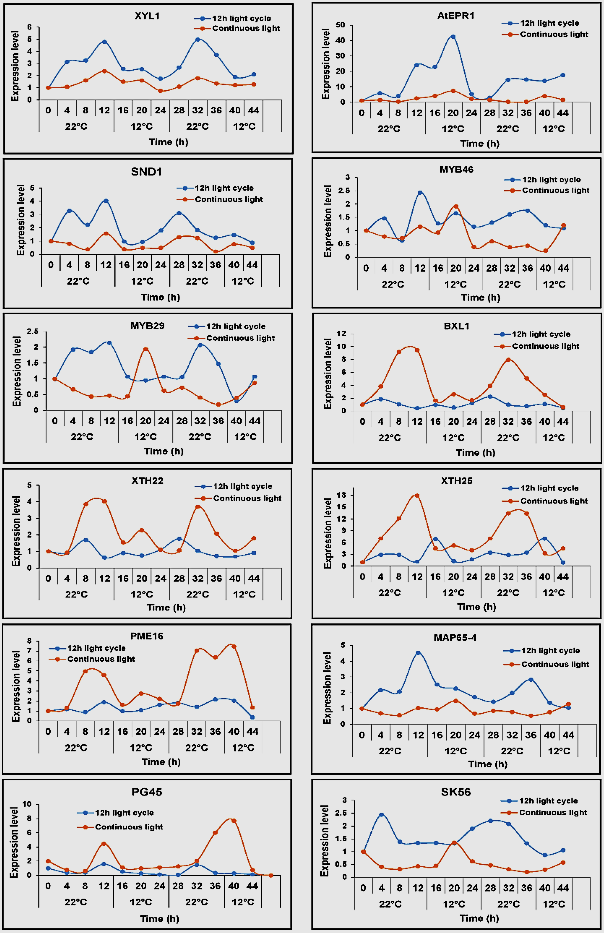


**Figure S16.** Rhythmic expression of genes that are regulated by SCL15 and involved in the regulation, biosynthesis and modification of cell wall.
